# Supplementary material for: Accommodating individual travel history and unsampled diversity in Bayesian phylogeographic inference of SARS-CoV-2
Source: Nat Commun. 2020 Oct 9;11:5110. doi: 10.1038/s41467-020-18877-9 (PMC7547076; doi:10.1038/s41467-020-18877-9)
Supplement: Supplementary file 1 — Supplementary Information [file 41467_2020_18877_MOESM1_ESM.pdf]

Supplementary Information for:

Accommodating individual travel history and unsampled diversity in Bayesian phylogeographic inference of SARS-CoV-2.

Authors: Philippe Lemey[1]*, Samuel Hong[1], Verity Hill[2], Guy Baele[1], Chiara Poletto[3], Vittoria Colizza[3], Áine O'Toole[2], John T. McCrone[2], Kristian G. Andersen[4], Michael Worobey[5], Martha I. Nelson[6], Andrew Rambaut[2] and Marc A. Suchard[7,8,9]*

Affiliations:

[1] KU Leuven Department of Microbiology, Immunology and Transplantation, Rega Institute, Laboratory of Clinical and Evolutionary Virology, Leuven, Belgium.

[2] Centre for Immunology, Infection and Evolution, University of Edinburgh, King's Buildings, Edinburgh, EH9 3FL, UK.

[3] INSERM, Sorbonne Université, Institut Pierre Louis d'Epidémiologie et de Santé Publique IPLESP, F75012 Paris, France.

[4] Department of Immunology and Microbiology, Scripps Research, La Jolla, CA 92037, USA.

[5] Department of Ecology and Evolutionary Biology, University of Arizona, Tucson, AZ 85721, USA.

[6] Division of International Epidemiology and Population Studies, Fogarty International Center, National Institutes of Health, Bethesda, Maryland 20892, USA.

[7] Department of Biomathematics, David Geffen School of Medicine, University of California Los Angeles, Los Angeles, CA 90095, USA.

[8] Department of Biostatistics, Fielding School of Public Health, University of California Los Angeles, Los Angeles, CA 90095, USA

[9] Department of Human Genetics, David Geffen School of Medicine, University of California Los Angeles, Los Angeles, CA 90095, USA

*Correspondence to: philippe.lemey@kuleuven.be, msuchard@ucla.edu

Supplementary Text 1: Bayesian Evaluation of Temporal Signal (BETS)

As part of the BETS analysis to assess temporal signal in the data, we estimate the performance of the strict clock and the relaxed clock with an underlying lognormal distribution, with and without making use of the sequence sampling times. We make use of generalized stepping-stone samling while accommodating phylogenetic uncertainty [1] to estimate log marginal likelihoods for these four models in BEAST 1.10 [2], running an initial Markov chain of 25 million iterations followed by 100 power posteriors of 1 million iterations each.

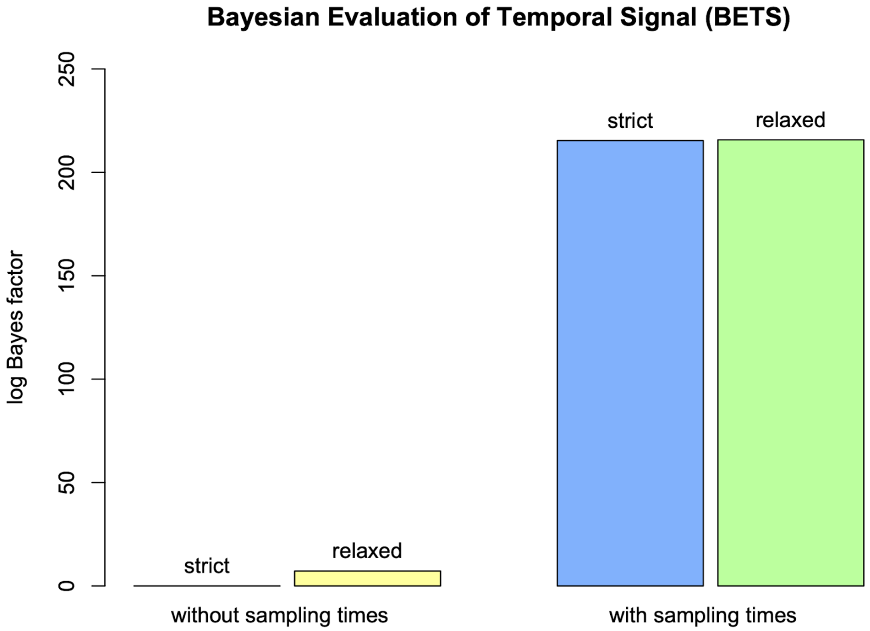

Figure S1: BETS reveals very strong evidence (log Bayes factors > 215) in favor of the models including sampling times, indicative of strong temporal signal in the data. The relaxed clock offers a marginal improvement of 0.3 log units in fit over the strict clock when using sampling times.

Figure S1 summarizes the results of the BETS analysis, which were repeated across multiple independent replicates and with increasing computational settings. When including the sampling times in the analysis of the data, model fit increases significantly and yields very

strong evidence (log Bayes factors > 215) in favor of using the sampling time information [3], with strict and relaxed clock offering near-identical performance (log Bayes factor of 0.3 in favor of the latter).

Supplementary Text 2: Empirical example illustrating the concept of including unsampled diversity.

We used a limited data set of 56 Zika virus (ZIKV) genomes sampled across Southeast Asia, the South Pacific islands and the Americas to demonstrate the concept of incorporating unsampled diversity in phylogeographic reconstructions (Table S1). Several studies have provided evidence that Zika Virus spread from Southeast Asia into French Polynesia (FP) and from there to both continental America as well as to other South Pacific islands [4–9]. This resulted in a single lineage comprising viral strains circulating in the South Pacific islands and Latin America, referred to as $ZIKV_{SP\text{-}AM}$. A Bayesian phylogeographic reconstruction based on the 56 genome data set confirms this pattern (Fig. S2A), clearly indicating the basal nature of the FP viruses in the $ZIKV_{SP\text{-}AM}$ lineage and a migration event from there to Brazil.

We focus on the key role of FP in the seeding of continental America and replace all FP genomes by unsampled taxa (Fig. S2B). In this analysis, the sampling times for the unsampled FP taxa were integrated over a normal prior distribution parameterized by estimates of the 2013-2014 FP epidemic curve [10] and the between-country diffusion process is parameterized using a GLM that considers air transportation distances as a potential covariate [11,12]. In summarizing the reconstruction, we remove the highly volatile unsampled taxa but we evaluate how they affect the posterior reconstruction of spatiotemporal spread. Our analysis indicates that even without any actual FP genomes, the 12 unsampled taxa ensure that FP is reconstructed as the location of origin for the continental America clade, and other South Pacific island viruses, with moderate support, (Fig. 2B). Including only a single FP genome in a standard reconstruction does not support FP at the origin of the continental America clade; in this case, the highest

support is associated with a direct transmission from Asia into Brazil (Fig. 2C). However, including 11 additional unsampled FP taxa with this genome in a GLM analysis reconstitutes the expected pattern of FP representing a stepping stone of ZIKV spread between Asia and Brazil (Fig. 2D).

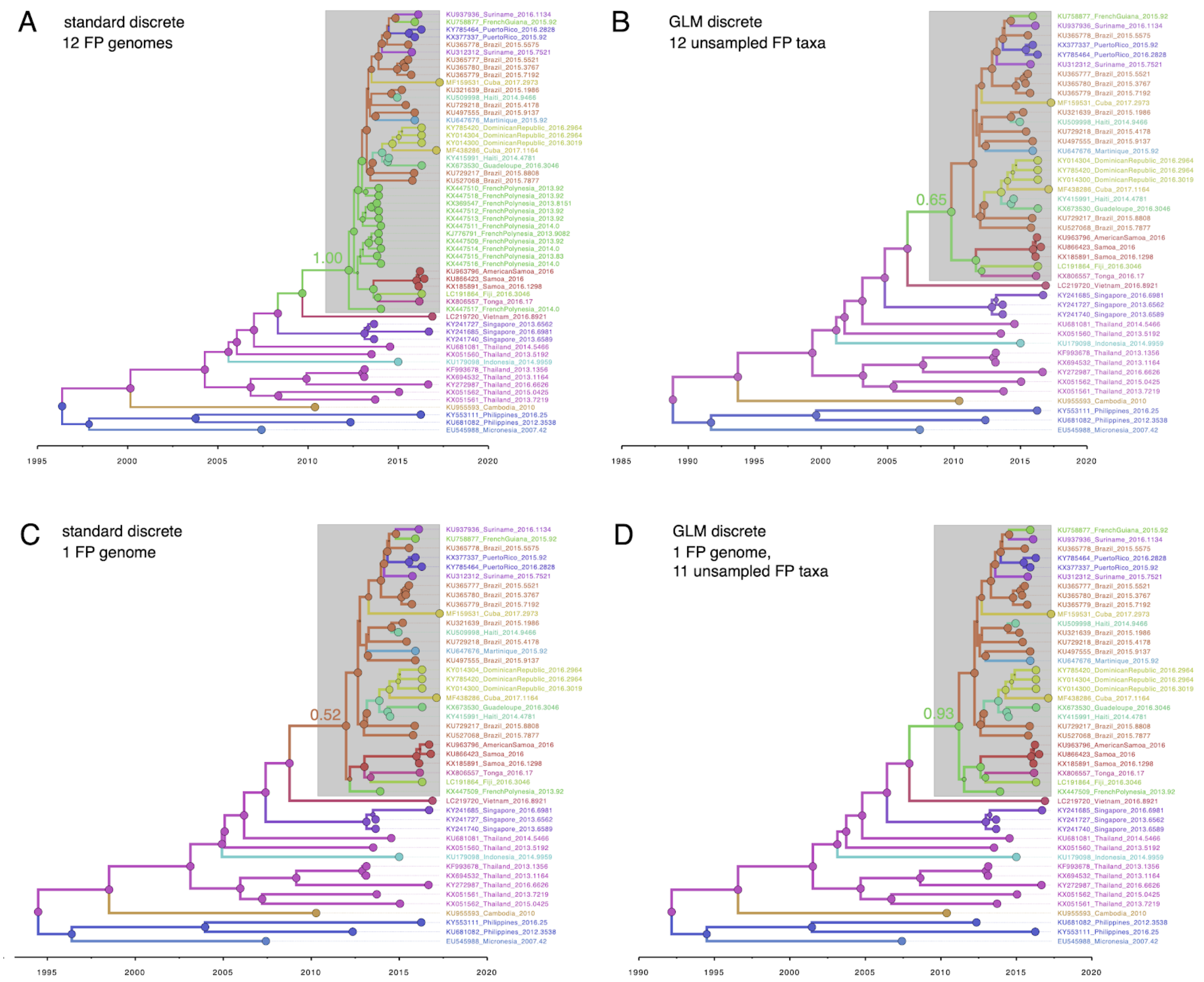

Figure S2: The impact of incorporating unsampled diversity on reconstructing phylogeographic spread. We compare a standard phylogeographic reconstruction for 56 ZIKV genomes including 12 FP genomes (A) to a GLM-diffusion phylogeographic reconstruction with air transportation with the 12 FP genomes replaced by unsampled taxa (B), a standard phylogeographic reconstruction using only a single FP genome (C), and to a GLM-diffusion analysis where the single FP genome is complemented with 11 unsampled taxa (D). The coloring represents the country of origin for the sampled/unsampled taxa as well as the country estimate for the internal node locations. The internal node circle sizes are proportional to posterior clade

support values. The values at the ancestral node for the $ZIKV_{SP-AM}$ lineage, highlighted in grey, represent the posterior location support at this node.

Supplementary Text 3: Phylogeographic predictor estimation including sample sizes

We fit a phylogeographic generalized linear model (GLM) that considers origin & destination sample size predictors in addition to air travel, geographic distance and asymmetry out of Hubei. These estimates indicate a considerably reduced contribution of sample sizes to the transitions rates in travel-aware inferences. The sample size at origin locations reduces from maximum posterior inclusion probability (>.999) to a low posterior inclusion probability (0.222). The destination sample size predictor remains associated with a maximum posterior inclusion probability, but has a lower Log conditional effect size in the travel aware inference. It is interesting to also note that the support for a flux out of Hubei and its log conditional effect size are only moderate when using sampling location only with sample size predictors. Thanks to the many genomes associated with a travel history from Hubei, the flux yields a high support and a very high effect size irrespective of the inclusion of sample sizes in the travel-aware inference.

Table 1: Inferred generalized linear model (GLM) of discrete location transitions when using sampling location only and sampling location augment with travel history. We report posterior inclusion probabilities and posterior mean (95% highest probability density intervals) log conditional effect sizes for air travel, geographic distance, asymmetry out of Hubei, and origin & destination sample size.

Predictor	Sampling location only		Sampling location and travel history	
	Inclusion probability	Log conditional effect size	Inclusion probability	Log conditional effect size
Geographic distance	0.168	0.059 (-0.033,0.159)	0.087	0.049 (-0.040,0.135)
Air Travel	0.995	0.760 (0.364,1.138)	0.985	0.486 (0.234,0.731)

Asymmetry out of Hubei	0.646	0.541 (-0.043,1.116)	>.999	2.154 (1.466,2.648)
Origin sample size	>.999	1.465 (0.933,2.188)	0.222	0.174 (-0.134,0.523)
Destination sample size	>.999	0.766 (0.560,0.975)	>.999	0.527 (0.527,0.865)

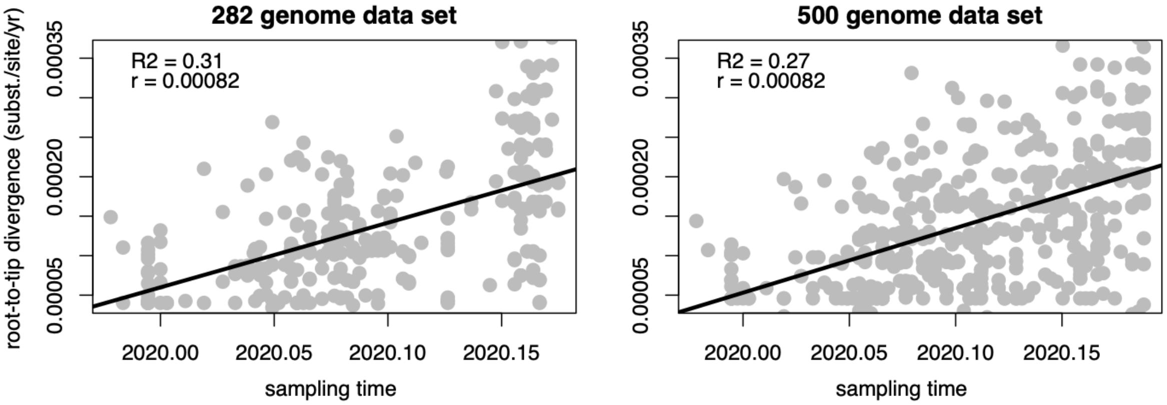

Figure S3: Root-to-tip divergence as a function of sampling time for the 282 and 500 genome data set. The top left values represent the R-squared and the slope (substitution rate) of the regression.

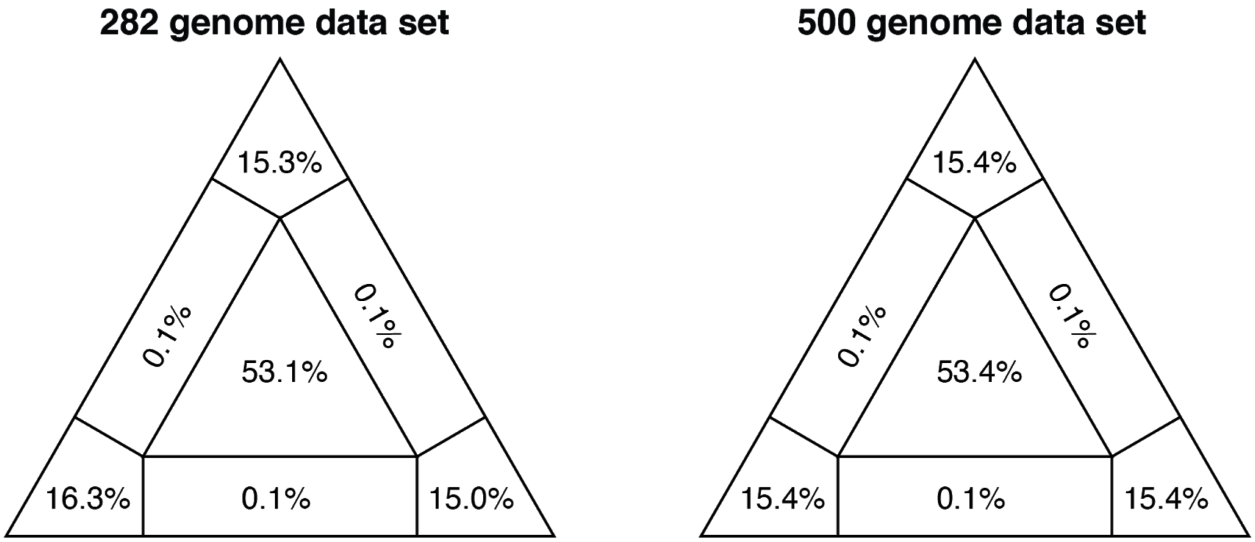

Figure S4: Likelihood mapping results for the 282 and 500 genome data set. The large percentage of quartets in the center of the simplex (>50%) reflects that a large amount of quartet trees exhibit a star-like topology and are therefore poorly resolved.

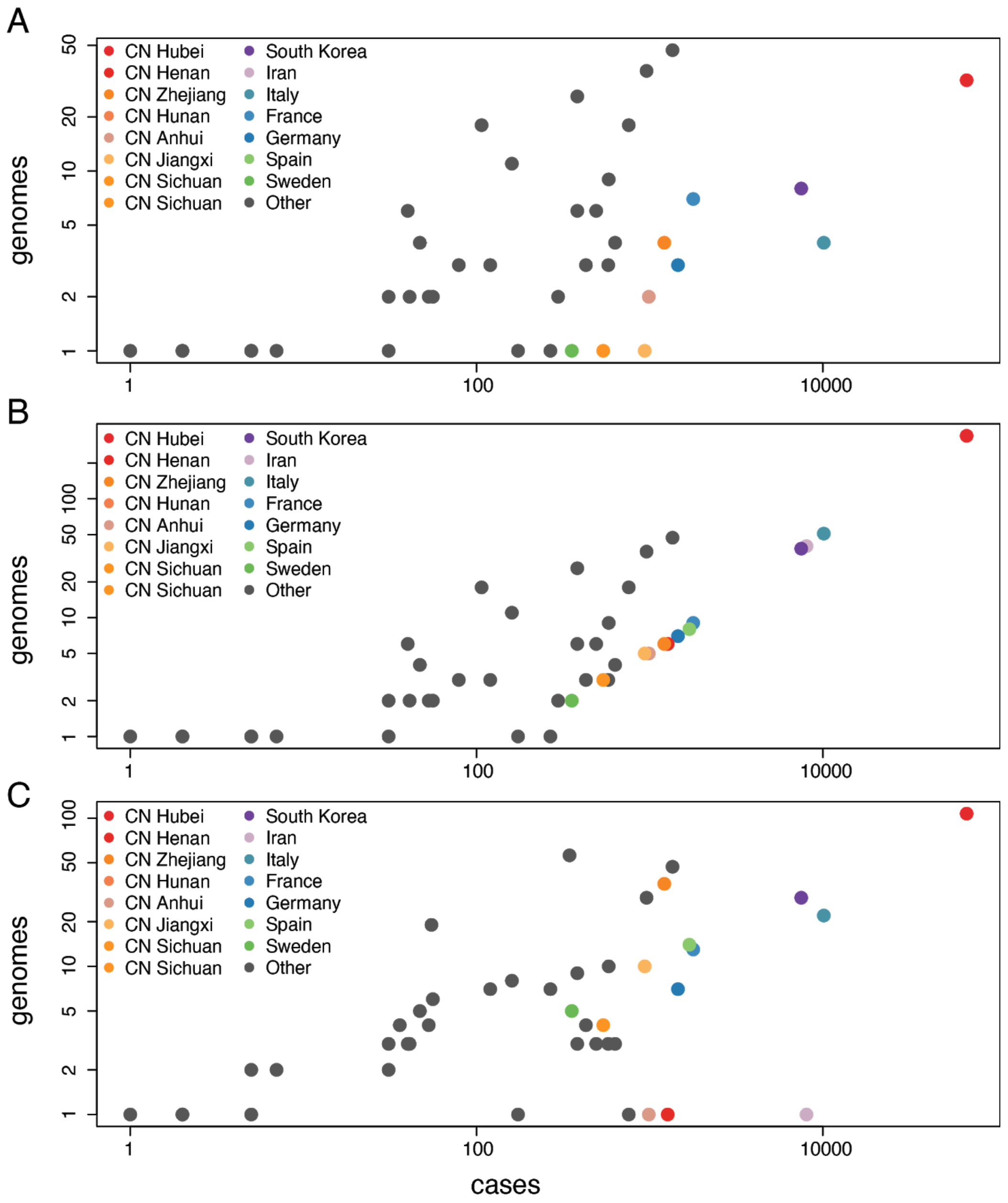

Figure S5: SARS-CoV-2 genomes and cases counts. (A) scatter plot of the number of available genomes against the number of cases on March 10 2020 on a log-log scale for the 282 genome data set. (B) Same as (A) but with additional unsampled taxa for 14 locations represented by colored data points. (C) Same as (A) for the 500 genome data set. The same colors are used for the data points that are considered to be under-sampled in (A) and (B).

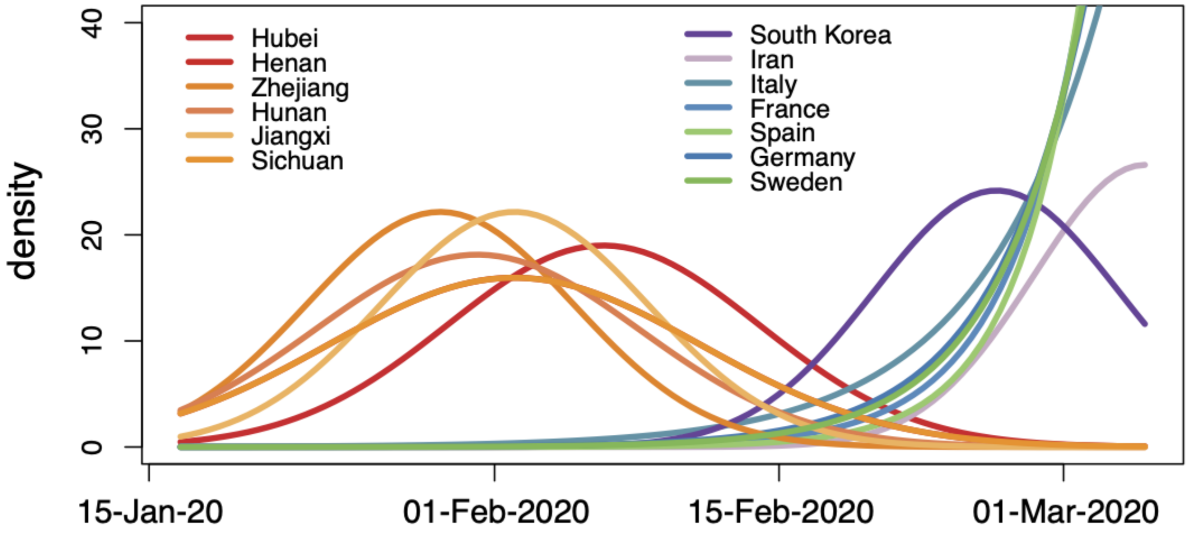

Figure S6: Prior probability distributions for the ages of the taxa representing unsampled diversity for different locations. The shapes of these probability distributions are based on estimated numbers of prevalent infections over time. The same normal prior probability distribution applies to taxa from Sichuan and Henan.

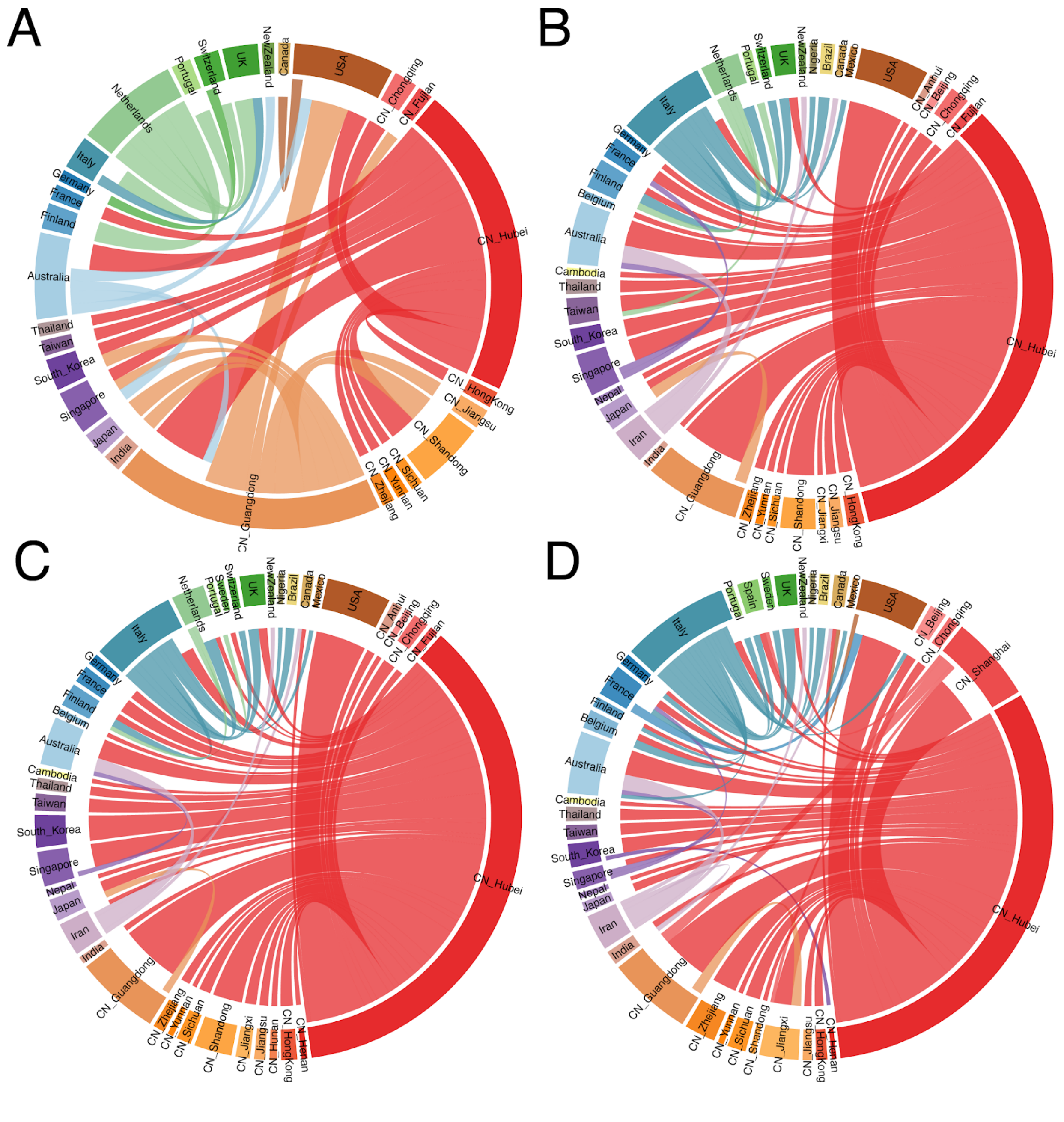

Figure S7. Circular migration flow plots summarizing Markov jump estimates for the analyses of the 282 genome data set using (A) sampling location only, (B) sampling location and travel history and (C) sampling location and travel history with unsampled diversity, and (D) for the analysis of the 500 genome data set using sampling location and travel history. The plots show the relative number of transitions between locations. The direction of the transitions are encoded both by the origin colour and by the gap between link and circle segment at the

destination (migration into a location is associated with a larger gap than migration out of a location).

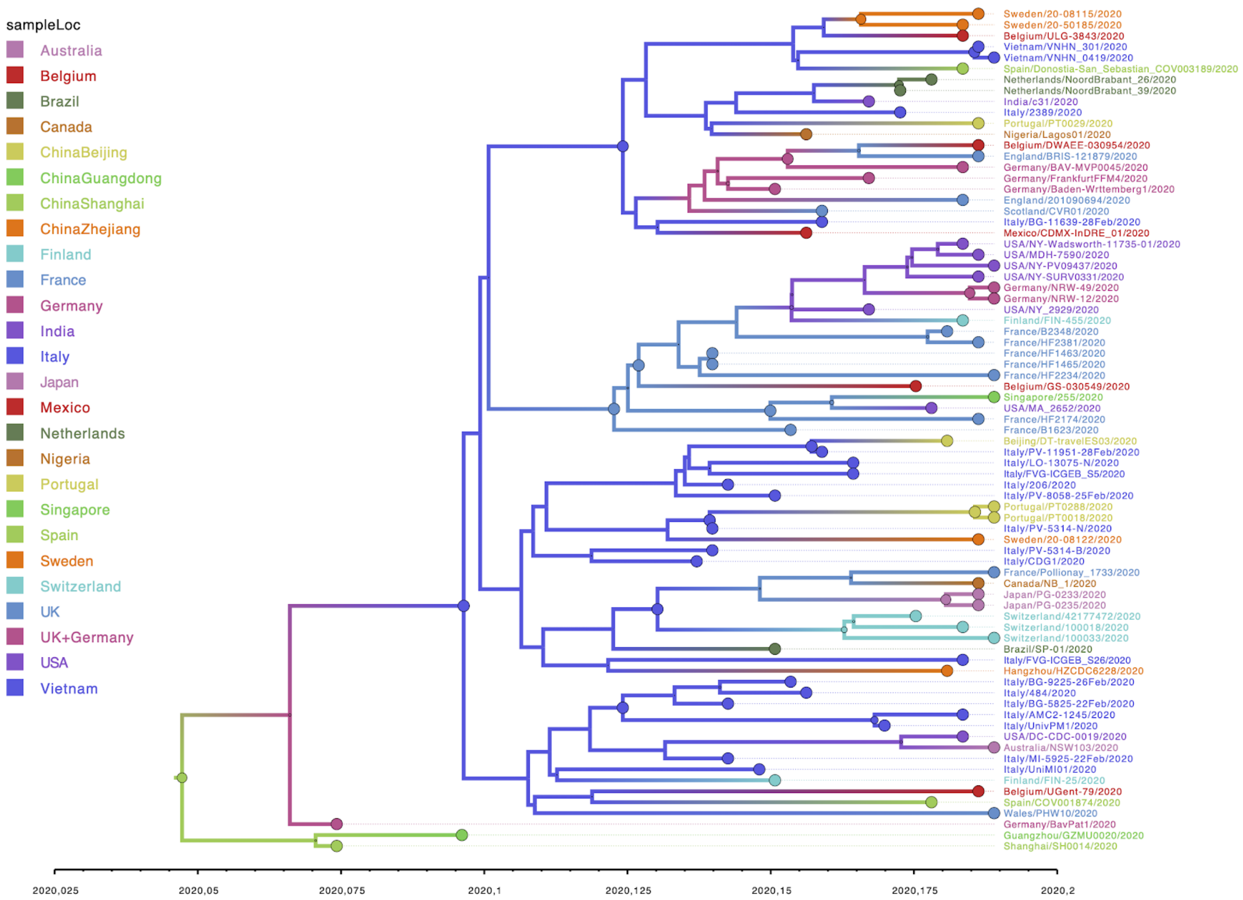

Figure S8. Maximum clade credibility subtree inferred for 500 genome data set. The subtree focuses on a European clade and closely related strains such as a virus from the first outbreak in Europe (Germany, BatPat). Genome sequences sampled at the tips of the phylogeny are represented by circles and strain names shaded according to location of sampling. Internal node circles, representing posterior clade support values, and branches are shaded similarly by inferred location.

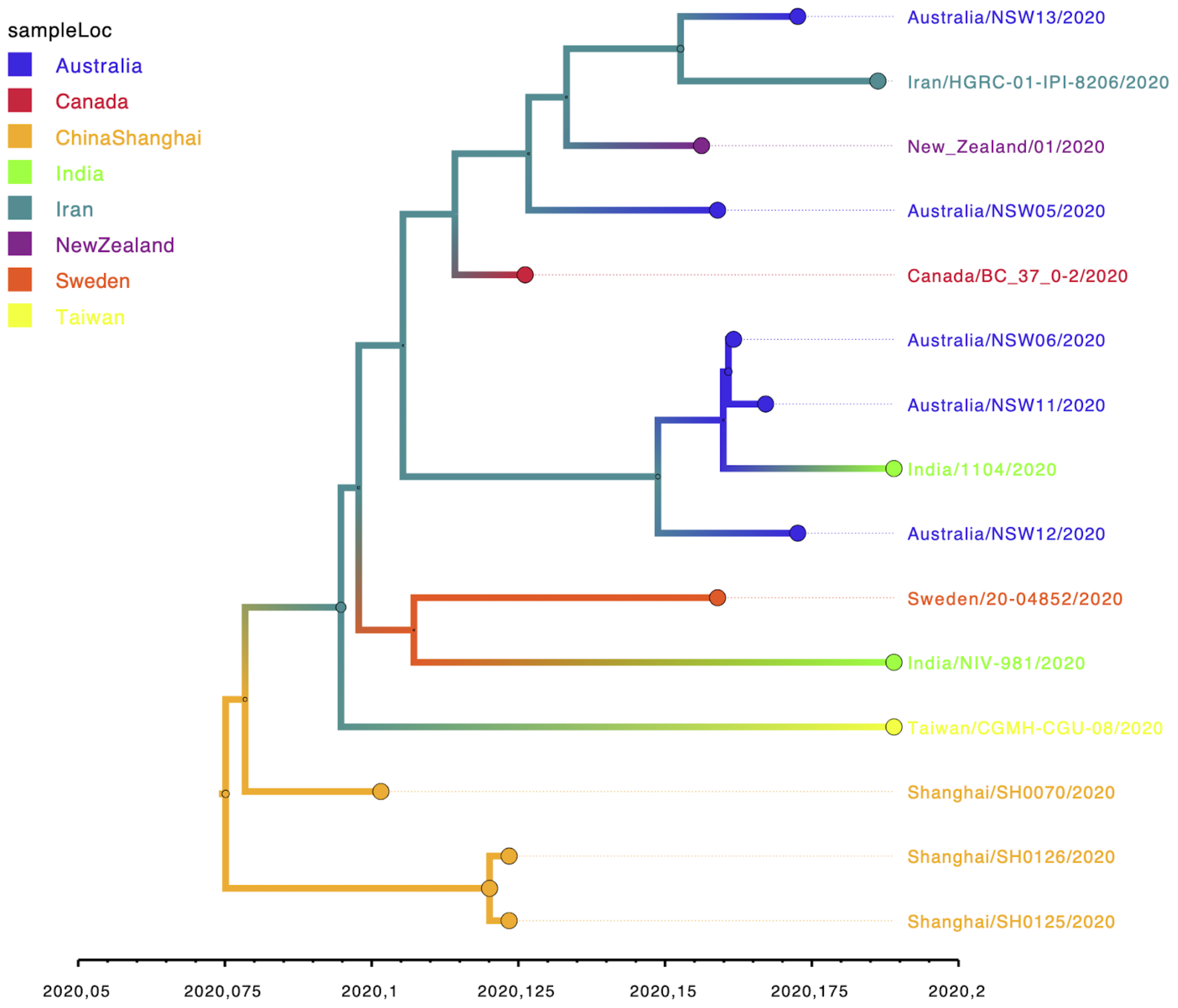

Figure S9. Maximum clade credibility subtree inferred for 500 genome data set. The subtree focuses on viruses from Australia (lineage B.4), New Zealand and Canada, many of which were sampled from travellers returning from Iran, and closely related viruses from different locations including one from Iran. Genome sequences sampled at the tips of the phylogeny are represented by circles and strain names shaded according to location of sampling. Internal node circles, representing posterior clade support values, and branches are shaded similarly by inferred location.

Table S2. Accession numbers, sampling country and sampling time for 56 ZIKV genomes.

Accession	Country	Sampling time
KU963796	American Samoa	2016
KU321639	Brazil	2015.1986
KU365777	Brazil	2015.5521
KU365778	Brazil	2015.5575
KU365779	Brazil	2015.7192
KU365780	Brazil	2015.3767
KU497555	Brazil	2015.9137
KU729217	Brazil	2015.8808
KU729218	Brazil	2015.4178
KU527068	Brazil	2015.7877
KU955593	Cambodia	2010
MF438286	Cuba	2017.1164
MF159531	Cuba	2017.2973
KY014300	Dominican Republic	2016.3019
KY014304	Dominican Republic	2016.2964
KY785420	Dominican Republic	2016.2964
LC191864	Fiji	2016.3046
KU758877	French Guiana	2015.92
KJ776791	French Polynesia	2013.9082
KX369547	French Polynesia	2013.8151
KX447509	French Polynesia	2013.92
KX447510	French Polynesia	2013.92
KX447511	French Polynesia	2014.0

KX447512	French Polynesia	2013.92
KX447513	French Polynesia	2013.92
KX447514	French Polynesia	2014.0
KX447515	French Polynesia	2013.83
KX447516	French Polynesia	2014.0
KX447517	French Polynesia	2014.0
KX447518	French Polynesia	2013.92
KX673530	Guadeloupe	2016.3046
KU509998	Haiti	2014.9466
KY415991	Haiti	2014.4781
KU179098	Indonesia	2014.9959
KU647676	Martinique	2015.92
EU545988	Micronesia	2007.42
KU681082	Philippines	2012.3538
KY553111	Philippines	2016.25
KX377337	PuertoRico	2015.92
KY785464	PuertoRico	2016.2828
KU866423	Samoa	2016
KX185891	Samoa	2016.1298
KY241685	Singapore	2016.6981
KY241727	Singapore	2013.6562
KY241740	Singapore	2013.6589
KU312312	Suriname	2015.7521
KU937936	Suriname	2016.1134
KU681081	Thailand	2014.5466

KX051560	Thailand	2013.5192
KX051561	Thailand	2013.7219
KX051562	Thailand	2015.0425
KX694532	Thailand	2013.1164
KY272987	Thailand	2016.6626
KF993678	Thailand	2013.1356
KX806557	Tonga	2016.17

Table S3. Travel history data associated with 64 of the 284 genomes.

GISAID accession number	sampling location	sampling date	travel location	Travel date (days before collection date)
EPI_ISL_403932	CN Guangdong	2020-01-14	CN Hubei	16
EPI_ISL_403936	CN Guangdong	2020-01-17	CN Hubei	6
EPI_ISL_403937	CN Guangdong	2020-01-18	CN Hubei	6
EPI_ISL_403962	Thailand	2020-01-08	CN Hubei	1*
EPI_ISL_403963	Thailand	2020-01-13	CN Hubei	1*
EPI_ISL_404227	CN Zhejiang	2020-01-16	CN Hubei	NA
EPI_ISL_404228	CN Zhejiang	2020-01-17	CN Hubei	NA
EPI_ISL_404253	USA	2020-01-21	CN Hubei	12
EPI_ISL_404895	USA	2020-01-19	CN Hubei	9
EPI_ISL_406034	USA	2020-01-23	CN Hubei	NA
EPI_ISL_406036	USA	2020-01-22	CN Hubei	NA
EPI_ISL_406223	USA	2020-01-22	CN Hubei	NA
EPI_ISL_406597	France	2020-01-23	CN Hubei	NA
EPI_ISL_406844	Australia	2020-01-25	CN Hubei	6
EPI_ISL_406862	Germany	2020-01-28	CN Hubei (2)	9 + x (2)
EPI_ISL_406970	CN Zhejiang	2020-01-20	CN Hubei	NA
EPI_ISL_406973	Singapore	2020-01-23	CN Hubei	NA
EPI_ISL_407079	Finland	2020-01-29	CN Hubei	6
EPI_ISL_407313	CN Zhejiang	2020-01-19	CN Hubei	NA
EPI_ISL_407893	Australia	2020-01-24	CN Hubei	6

EPI_ISL_407894	Australia	2020-01-28	CN Hubei	1
EPI_ISL_407896	Australia	2020-01-30	CN Hubei	3
EPI_ISL_407976	Belgium	2020-02-03	CN Hubei	2
EPI_ISL_408008	USA	2020-01-29	CN Hubei	NA
EPI_ISL_408010	USA	2020-01-29	CN Hubei	6
EPI_ISL_408489	Taiwan	2020-01-31	CN Hubei	11
EPI_ISL_408668	Vietnam	2020-01-24	CN Hubei	7
EPI_ISL_408670	USA	2020-01-31	CN Beijing	NA
EPI_ISL_408976	Australia	2020-01-22	CN Hubei	2
EPI_ISL_408977	Australia	2020-01-25	CN Hubei	2
EPI_ISL_409067	USA	2020-01-29	CN Hubei	1
EPI_ISL_410218	Taiwan	2020-02-05	CN Hubei	NA
EPI_ISL_410301	Nepal	2020-01-13	CN Hubei	1*
EPI_ISL_410486	France	2020-02-08	Singapore (2)	15 (2)
EPI_ISL_410545	Italy	2020-01-29	CN Hubei	6
EPI_ISL_410717	Australia	2020-02-05	CN Hubei	9
EPI_ISL_410718	Australia	2020-02-05	CN Hubei	9
EPI_ISL_411902	Cambodia	2020-01-27	CN Hubei	4
EPI_ISL_411929	South Korea	2020-01	CN Hubei	NA
EPI_ISL_411951	Sweden	2020-02-07	CN Hubei	14
EPI_ISL_411954	USA	2020-02-06	CN Hubei	1
EPI_ISL_411955	USA	2020-02-10	CN Hubei	5
EPI_ISL_411956	USA	2020-02-11	CN Hubei	6
EPI_ISL_412116	UK	2020-02-09	China	2
EPI_ISL_412912	Germany	2020-02-25	Italy	NA

EPI_ISL_412964	Brazil	2020-02-25	Italy	4
EPI_ISL_412965	Canada	2020-02-16	Iran	7
EPI_ISL_412971	Finland	2020-02-25	Italy	2
EPI_ISL_412972	Mexico	2020-02-27	Italy	5
EPI_ISL_412975	Australia	2020-02-28	Iran	6
EPI_ISL_413014	Canada	2020-01-25	CN Hubei	7
EPI_ISL_413015	Canada	2020-01-23	CN Hubei	NA
EPI_ISL_413016	Brazil	2020-02-28	Italy	1
EPI_ISL_413213	Australia	2020-02-29	Iran	6
EPI_ISL_413221	UK	2020-03-02	Italy	7
EPI_ISL_413490	New Zealand	2020-02-27	Iran	1
EPI_ISL_413522	India	2020-01-27	China	NA
EPI_ISL_413523	India	2020-01-27	China	10
EPI_ISL_413550	Nigeria	2020-02-27	Italy	2
EPI_ISL_413594	Australia	2020-02-28	Singapore	1*
EPI_ISL_413596	Australia	2020-02-28	SE Asia	NA
EPI_ISL_413597	Australia	2020-03-02	Iran	3
EPI_ISL_413598	Australia	2020-03-04	Iran	3
EPI_ISL_413599	Australia	2020-03-04	Iran	NA

* when the collection date was the same as the travel date, a minimum of 1 day prior to the collection date was used as the date of travel.
(2) the travel location and date was used for the person having infected the sampled person. For EPI_ISL_406862, x was set to 5 days.

Table S4. Acknowledgements table for the 282 genome data set used in this study

Accession ID	Virus name	Location	Collection date	Originating lab	Submitting lab	Authors
**EPI_ISL_402119**	hCoV-19/Wuhan/IVDC-HB-01/2019	Asia / China / Hubei / Wuhan	2019-12-30	National Institute for Viral Disease Control and Prevention, China CDC	National Institute for Viral Disease Control and Prevention, China CDC	Wenjie Tan，Xiang Zhao，Wenling Wang，Xuejun Ma，Yongzhong Jiang，Roujian Lu，Ji Wang，Weimin Zhou，Peihua Niu，Peipei Liu，Faxian Zhan，Weifeng Shi，Baoying Huang，Jun Liu，Li Zhao，Yao Meng，Xiaozhou He，Fei Ye，Na Zhu，Yang Li，Jing Chen，Wenbo Xu，George F. Gao，Guizhen Wu
**EPI_ISL_402120**	hCoV-19/Wuhan/IVDC-HB-04/2020	Asia / China / Hubei / Wuhan	2020-01-01	National Institute for Viral Disease Control and Prevention, China CDC	National Institute for Viral Disease Control and Prevention, China CDC	Wenjie Tan，Xiang Zhao，Wenling Wang，Xuejun Ma，Yongzhong Jiang，Roujian Lu，Ji Wang，Weimin Zhou，Peihua Niu，Peipei Liu，Faxian Zhan，Weifeng Shi，Baoying Huang，Jun Liu，Li Zhao，Yao Meng，Xiaozhou He，Fei Ye，Na Zhu，Yang Li，Jing Chen，Wenbo Xu，George F. Gao，Guizhen Wu
**EPI_ISL_402121**	hCoV-19/Wuhan/IVDC-HB-05/2019	Asia / China / Hubei / Wuhan	2019-12-30	National Institute for Viral Disease Control and Prevention, China CDC	National Institute for Viral Disease Control and Prevention, China CDC	Wenjie Tan，Xuejun Ma，Xiang Zhao，Wenling Wang，Yongzhong Jiang，Roujian Lu，Ji Wang，Peihua Niu，Weimin Zhou，Faxian Zhan，Weifeng Shi，Baoying Huang，Jun Liu，Li Zhao，Yao Meng，Fei Ye，Na Zhu，Xiaozhou He，Peipei Liu，Yang Li，Jing Chen，Wenbo Xu，George F. Gao，Guizhen Wu
**EPI_ISL_402123**	hCoV-19/Wuhan/IPBCAMS-WH-01/2019	Asia / China / Hubei / Wuhan	2019-12-24	Institute of Pathogen Biology, Chinese Academy of Medical Sciences & Peking Union Medical College	Institute of Pathogen Biology, Chinese Academy of Medical Sciences & Peking Union Medical College	Lili Ren, Jianwei Wang, Qi Jin, Zichun Xiang, Zhiqiang Wu, Chao Wu, Yiwei Liu
**EPI_ISL_402124**	hCoV-19/Wuhan/WIV04/2019	Asia / China / Hubei / Wuhan	2019-12-30	Wuhan Jinyintan Hospital	Wuhan Institute of Virology, Chinese Academy of Sciences	Peng Zhou, Xing-Lou Yang, Ding-Yu Zhang, Lei Zhang, Yan Zhu, Hao-Rui Si, Zhengli Shi
**EPI_ISL_402125**	hCoV-19/Wuhan-Hu-1/2019	Asia / China	2019-12-31	unknown	National Institute for Communicable Disease Control and Prevention (ICDC) Chinese Center for Disease Control and Prevention (China CDC)	Zhang,Y.-Z., Wu,F., Chen,Y.-M., Pei,Y.-Y., Xu,L., Wang,W., Zhao,S., Yu,B., Hu,Y., Tao,Z.-W., Song,Z.-G., Tian,J.-H., Zhang,Y.-L., Liu,Y., Zheng,J.-J., Dai,F.-H., Wang,Q.-M., She,J.-L. and Zhu,T.-Y.
**EPI_ISL_402127**	hCoV-19/Wuhan/WIV02/2019	Asia / China / Hubei / Wuhan	2019-12-30	Wuhan Jinyintan Hospital	Wuhan Institute of Virology, Chinese Academy of Sciences	Peng Zhou, Xing-Lou Yang, Ding-Yu Zhang, Lei Zhang, Yan Zhu, Hao-Rui Si, Zhengli Shi
**EPI_ISL_402128**	hCoV-19/Wuhan/WIV05/2019	Asia / China / Hubei / Wuhan	2019-12-30	Wuhan Jinyintan Hospital	Wuhan Institute of Virology, Chinese Academy of Sciences	Peng Zhou, Xing-Lou Yang, Ding-Yu Zhang, Lei Zhang, Yan Zhu, Hao-Rui Si, Zhengli Shi
**EPI_ISL_402129**	hCoV-19/Wuhan/WIV06/2019	Asia / China / Hubei / Wuhan	2019-12-30	Wuhan Jinyintan Hospital	Wuhan Institute of Virology, Chinese Academy of Sciences	Peng Zhou, Xing-Lou Yang, Ding-Yu Zhang, Lei Zhang, Yan Zhu, Hao-Rui Si, Zhengli Shi
**EPI_ISL_402130**	hCoV-19/Wuhan/WIV07/2019	Asia / China / Hubei / Wuhan	2019-12-30	Wuhan Jinyintan Hospital	Wuhan Institute of Virology, Chinese Academy of Sciences	Peng Zhou, Xing-Lou Yang, Ding-Yu Zhang, Lei Zhang, Yan Zhu, Hao-Rui Si, Zhengli Shi
**EPI_ISL_402132**	hCoV-19/Wuhan/HBCDC-HB-01/2019	Asia / China / Hubei / Wuhan	2019-12-30	Wuhan Jinyintan Hospital	Hubei Provincial Center for Disease Control and Prevention	Bin Fang, Xiang Li, Xiao Yu, Linlin Liu, Bo Yang, Faxian Zhan, Guojun Ye, Xixiang Huo, Junqiang Xu, Bo Yu, Kun Cai, Jing Li, Yongzhong Jiang.
**EPI_ISL_403928**	hCoV-19/Wuhan/IPBCAMS-WH-05/2020	Asia / China / Hubei / Wuhan	2020-01-01	Institute of Pathogen Biology, Chinese Academy	Institute of Pathogen Biology, Chinese Academy of Medical	Lili Ren, Jianwei Wang, Qi Jin, Zichun Xiang, Zhiqiang Wu, Chao Wu, Yiwei Liu

				of Medical Sciences & Peking Union Medical College	Sciences & Peking Union Medical College	
**EPI_ISL_403929**	hCoV-19/Wuhan/IPBCAMS-WH-04/2019	Asia / China / Hubei / Wuhan	2019-12-30	Institute of Pathogen Biology, Chinese Academy of Medical Sciences & Peking Union Medical College	Institute of Pathogen Biology, Chinese Academy of Medical Sciences & Peking Union Medical College	Lili Ren, Jianwei Wang, Qi Jin, Zichun Xiang, Zhiqiang Wu, Chao Wu, Yiwei Liu
**EPI_ISL_403930**	hCoV-19/Wuhan/IPBCAMS-WH-03/2019	Asia / China / Hubei / Wuhan	2019-12-30	Institute of Pathogen Biology, Chinese Academy of Medical Sciences & Peking Union Medical College	Institute of Pathogen Biology, Chinese Academy of Medical Sciences & Peking Union Medical College	Lili Ren, Jianwei Wang, Qi Jin, Zichun Xiang, Zhiqiang Wu, Chao Wu, Yiwei Liu
**EPI_ISL_403931**	hCoV-19/Wuhan/IPBCAMS-WH-02/2019	Asia / China / Hubei / Wuhan	2019-12-30	Institute of Pathogen Biology, Chinese Academy of Medical Sciences & Peking Union Medical College	Institute of Pathogen Biology, Chinese Academy of Medical Sciences & Peking Union Medical College	Lili Ren, Jianwei Wang, Qi Jin, Zichun Xiang, Zhiqiang Wu, Chao Wu, Yiwei Liu
**EPI_ISL_403932**	hCoV-19/Guangdong/20SF012/2020	Asia / China / Guangdong / Shenzhen	2020-01-14	Guangdong Provincial Center for Diseases Control and Prevention; Guangdong Provincial Public Health	Department of Microbiology, Guangdong Provincial Center for Diseases Control and Prevention	Min Kang, Jie Wu, Jing Lu, Tao Liu, Baisheng Li, Shujiang Mei, Feng Ruan, Lifeng Lin, Changwen Ke, Haojie Zhong, Yingtao Zhang, Lirong Zou, Xuguang Chen, Qi Zhu, Jianpeng Xiao, Jianxiang Geng, Zhe Liu, Jianxiong Hu, Weilin Zeng, Xing Li, Yuhuang Liao, Xiujuan Tang, Songjian Xiao, Ying Wang, Yingchao Song, Xue Zhuang, Lijun Liang, Guanhao He, Huihong Deng, Tie Song, Jianfeng He, Wenjun Ma
**EPI_ISL_403933**	hCoV-19/Guangdong/20SF013/2020	Asia / China / Guangdong / Shenzhen	2020-01-15	Guangdong Provincial Center for Diseases Control and Prevention; Guangdong Provincial Public Health	Department of Microbiology, Guangdong Provincial Center for Diseases Control and Prevention	Min Kang, Jie Wu, Jing Lu, Tao Liu, Baisheng Li, Shujiang Mei, Feng Ruan, Lifeng Lin, Changwen Ke, Haojie Zhong, Yingtao Zhang, Lirong Zou, Xuguang Chen, Qi Zhu, Jianpeng Xiao, Jianxiang Geng, Zhe Liu, Jianxiong Hu, Weilin Zeng, Xing Li, Yuhuang Liao, Xiujuan Tang, Songjian Xiao, Ying Wang, Yingchao Song, Xue Zhuang, Lijun Liang, Guanhao He, Huihong Deng, Tie Song, Jianfeng He, Wenjun Ma
**EPI_ISL_403934**	hCoV-19/Guangdong/20SF014/2020	Asia / China / Guangdong / Shenzhen	2020-01-15	Guangdong Provincial Center for Diseases Control and Prevention; Guangdong Provincial Public Health	Department of Microbiology, Guangdong Provincial Center for Diseases Control and Prevention	Min Kang, Jie Wu, Jing Lu, Tao Liu, Baisheng Li, Shujiang Mei, Feng Ruan, Lifeng Lin, Changwen Ke, Haojie Zhong, Yingtao Zhang, Lirong Zou, Xuguang Chen, Qi Zhu, Jianpeng Xiao, Jianxiang Geng, Zhe Liu, Jianxiong Hu, Weilin Zeng, Xing Li, Yuhuang Liao, Xiujuan Tang, Songjian Xiao, Ying Wang, Yingchao Song, Xue Zhuang, Lijun Liang, Guanhao He, Huihong Deng, Tie Song, Jianfeng He, Wenjun Ma
**EPI_ISL_403935**	hCoV-19/Guangdong/20SF025/2020	Asia / China / Guangdong / Shenzhen	2020-01-15	Guangdong Provincial Center for Diseases Control and Prevention; Guangdong Provincial Public Health	Department of Microbiology, Guangdong Provincial Center for Diseases Control and Prevention	Min Kang, Jie Wu, Jing Lu, Tao Liu, Baisheng Li, Shujiang Mei, Feng Ruan, Lifeng Lin, Changwen Ke, Haojie Zhong, Yingtao Zhang, Lirong Zou, Xuguang Chen, Qi Zhu, Jianpeng Xiao, Jianxiang Geng, Zhe Liu, Jianxiong Hu, Weilin Zeng, Xing Li, Yuhuang Liao, Xiujuan Tang, Songjian Xiao, Ying Wang, Yingchao Song, Xue Zhuang, Lijun Liang, Guanhao He, Huihong Deng, Tie Song, Jianfeng He, Wenjun Ma
**EPI_ISL_403936**	hCoV-19/Guangdong/20SF028/2020	Asia / China / Guangdong / Zhuhai	2020-01-17	Guangdong Provincial Center for Diseases Control and	Department of Microbiology, Guangdong Provincial Center for Diseases	Min Kang, Jie Wu, Jing Lu, Tao Liu, Baisheng Li, Shujiang Mei, Feng Ruan, Lifeng Lin, Changwen Ke, Haojie Zhong, Yingtao Zhang, Lirong Zou, Xuguang Chen, Qi Zhu, Jianpeng Xiao, Jianxiang

				Prevention; Guangdong Provincial Public Health	Control and Prevention	Geng, Zhe Liu, Jianxiong Hu, Weilin Zeng, Xing Li, Yuhuang Liao, Xiujuan Tang, Songjian Xiao, Ying Wang, Yingchao Song, Xue Zhuang, Lijun Liang, Guanhao He, Huihong Deng, Tie Song, Jianfeng He, Wenjun Ma
**EPI_ISL_403937**	hCoV-19/Guangdong/20SF040/2020	Asia / China / Guangdong / Zhuhai	2020-01-18	Guangdong Provincial Center for Diseases Control and Prevention; Guangdong Provincial Public Health	Department of Microbiology, Guangdong Provincial Center for Diseases Control and Prevention	Min Kang, Jie Wu, Jing Lu, Tao Liu, Baisheng Li, Shujiang Mei, Feng Ruan, Lifeng Lin, Changwen Ke, Haojie Zhong, Yingtao Zhang, Lirong Zou, Xuguang Chen, Qi Zhu, Jianpeng Xiao, Jianxiang Geng, Zhe Liu, Jianxiong Hu, Weilin Zeng, Xing Li, Yuhuang Liao, Xiujuan Tang, Songjian Xiao, Ying Wang, Yingchao Song, Xue Zhuang, Lijun Liang, Guanhao He, Huihong Deng, Tie Song, Jianfeng He, Wenjun Ma
**EPI_ISL_403962**	hCoV-19/Thailand/61/2020	Asia / Thailand / Nonthaburi	2020-01-08	Bamrasnaradura Hospital	1. Department of Medical Sciences, Ministry of Public Health, Thailand 2. Thai Red Cross Emerging Infectious Diseases - Health Science Centre 3. Department of Disease Control, Ministry of Public Health, Thailand	Pilailuk,Okada; Siripaporn,Phuygun; Thanutsapa,Thanadachakul; Supaporn,Wacharapluesadee; Sittiporn,Parnmen; Warawan,Wongboot; Sunthareeya,Waicharoen; Rome,Buathong; Malinee,Chittaganpitch; Nanthawan,Mekha
**EPI_ISL_403963**	hCoV-19/Thailand/74/2020	Asia / Thailand / Nonthaburi	2020-01-13	Bamrasnaradura Hospital	1. Department of Medical Sciences, Ministry of Public Health, Thailand 2. Thai Red Cross Emerging Infectious Diseases - Health Science Centre 3. Department of Disease Control, Ministry of Public Health, Thailand	Pilailuk,Okada; Siripaporn,Phuygun; Thanutsapa,Thanadachakul; Supaporn,Wacharapluesadee; Sittiporn,Parnmen; Warawan,Wongboot; Sunthareeya,Waicharoen; Rome,Buathong; Malinee,Chittaganpitch; Nanthawan,Mekha
**EPI_ISL_404227**	hCoV-19/Zhejiang/WZ-01/2020	Asia / China / Zhejiang	2020-01-16	Zhejiang Provincial Center for Disease Control and Prevention	Department of Microbiology, Zhejiang Provincial Center for Disease Control and Prevention	Yin Chen, Yanjun Zhang, Haiyan Mao, Junhang Pan, Xiuyu Lou, Yiyu Lu, Juying Yan, Hanping Zhu, Jian Gao, Yan Feng, Yi Sun, Hao Yan, Zhen Li, Yisheng Sun, Liming Gong, Qiong Ge, Wen Shi, Xinying Wang, Wenwu Yao, Zhangnv Yang, Fang Xu, Chen Chen, Enfu Chen, Zhen Wang, Zhiping Chen, Jianmin Jiang, Chonggao Hu
**EPI_ISL_404228**	hCoV-19/Zhejiang/WZ-02/2020	Asia / China / Zhejiang	2020-01-17	Zhejiang Provincial Center for Disease Control and Prevention	Department of Microbiology, Zhejiang Provincial Center for Disease Control and Prevention	Yanjun Zhang, Yin Chen, Haiyan Mao, Junhang Pan, Xiuyu Lou, Yiyu Lu, Juying Yan, Hanping Zhu, Jian Gao, Yan Feng, Yi Sun, Hao Yan, Zhen Li, Yisheng Sun, Liming Gong, Qiong Ge, Wen Shi, Xinying Wang, Wenwu Yao, Zhangnv Yang, Fang Xu, Chen Chen, Enfu Chen, Zhen Wang, Zhiping Chen, Jianmin Jiang, Chonggao Hu
**EPI_ISL_404253**	hCoV-19/USA/IL1/2020	North America / USA / Illinois / Chicago	2020-01-21	IL Department of Public Health Chicago Laboratory	Pathogen Discovery, Respiratory Viruses Branch, Division of Viral Diseases, Centers for Dieases Control and Prevention	Ying Tao, Krista Queen, Clinton R. Paden, Jing Zhang, Yan Li, Anna Uehara, Xiaoyan Lu, Brian Lynch, Senthil Kumar K. Sakthivel, Brett L. Whitaker, Shifaq Kamili, Lijuan Wang, Janna' R. Murray, Susan I. Gerber, Stephen Lindstrom, Suxiang Tong
**EPI_ISL_404895**	hCoV-19/USA/WA1/2020	North America / USA / Washington / Snohomish County	2020-01-19	Providence Regional Medical Center	Division of Viral Diseases, Centers for Disease Control and Prevention	Queen,K., Tao,Y., Li,Y., Paden,C.R., Lu,X., Zhang,J., Gerber,S.I., Lindstrom,S., Tong,S.

**EPI_ISL_405839**	hCoV-19/Shenzhen/HKU-SZ-005/2020	Asia / China / Guangdong / Shenzhen	2020-01-11	The University of Hong Kong - Shenzhen Hospital	Li Ka Shing Faculty of Medicine, The University of Hong Kong	Chan,J.F.-W., Yuan,S., Kok,K.H., To,K.K.-W., Chu,H., Yang,J., Xing,F., Liu,J., Yip,C.C.-Y., Poon,R.W.-S., Tsai,H.W., Lo,S.K.-F., Chan,K.H., Poon,V.K.-M., Chan,W.M., Ip,J.D., Cai,J.P., Cheng,V.C.-C., Chen,H., Hui,C.K.-M. and Yuen,K.Y.
**EPI_ISL_406031**	hCoV-19/Taiwan/2/2020	Asia / Taiwan / Kaohsiung	2020-01-23	Centers for Disease Control, R.O.C. (Taiwan)	Centers for Disease Control, R.O.C. (Taiwan)	Ji-Rong Yang, Yu-Chi Lin, Jung-Jung Mu, Ming-Tsan Liu, Shu-Ying Li
**EPI_ISL_406034**	hCoV-19/USA/CA1/2020	North America / USA / California / Los Angeles	2020-01-23	California Department of Public Health	Pathogen Discovery, Respiratory Viruses Branch, Division of Viral Diseases, Centers for Dieases Control and Prevention	Anna Uehara, Krista Queen, Ying Tao, Yan Li, Clinton R. Paden, Jing Zhang, Xiaoyan Lu, Brian Lynch, Senthil Kumar K. Sakthivel, Brett L. Whitaker, Shifaq Kamili, Lijuan Wang, Janna' R. Murray, Susan I. Gerber, Stephen Lindstrom, Suxiang Tong
**EPI_ISL_406036**	hCoV-19/USA/CA2/2020	North America / USA / California / Orange County	2020-01-22	California Department of Public Health	Pathogen Discovery, Respiratory Viruses Branch, Division of Viral Diseases, Centers for Dieases Control and Prevention	Anna Uehara, Krista Queen, Ying Tao, Yan Li, Clinton R. Paden, Jing Zhang, Xiaoyan Lu, Brian Lynch, Senthil Kumar K. Sakthivel, Brett L. Whitaker, Shifaq Kamili, Lijuan Wang, Janna' R. Murray, Susan I. Gerber, Stephen Lindstrom, Suxiang Tong
**EPI_ISL_406223**	hCoV-19/USA/AZ1/2020	North America / USA / Arizona / Phoenix	2020-01-22	Arizona Department of Health Services	Pathogen Discovery, Respiratory Viruses Branch, Division of Viral Diseases, Centers for Disease Control and Prevention	Ying Tao, Clinton R. Paden, Krista Queen, Anna Uehara, Yan Li, Jing Zhang, Xiaoyan Lu, Brian Lynch, Senthil Kumar K. Sakthivel, Brett L. Whitaker, Shifaq Kamili, Lijuan Wang, Janna' R. Murray, Susan I. Gerber, Stephen Lindstrom, Suxiang Tong
**EPI_ISL_406531**	hCoV-19/Guangdong/20SF174/2020	Asia / China / Guangdong / Zhuhai	2020-01-22	Guangdong Provincial Center for Diseases Contorl and Prevention; Guangdong Provincial Public Health	Guangdong Provincial Center for Disease Control and Prevention	Min Kang, Jie Wu, Jing Lu, Tao Liu, Baisheng Li, Shujiang Mei, Feng Ruan, Lifeng Lin, Changwen Ke, Haojie Zhong, Yingtao Zhang, Lirong Zou, Xuguang Chen, Qi Zhu, Jianpeng Xiao, Jianxiang Geng, Zhe Liu, Jianxiong Hu, Weilin Zeng, Xing Li, Yuhuang Liao, Xiujuan Tang, Songjian Xiao, Ying Wang, Yingchao Song, Xue Zhuang, Lijun Liang, Guanhao He, Huihong Deng, Tie Song, Jianfeng He, Wenjun Ma
**EPI_ISL_406533**	hCoV-19/Guangzhou/20SF206/2020	Asia / China / Guangdong / Guangzhou	2020-01-22	Guangdong Provincial Center for Diseases Contorl and Prevention; Guangdong Provincial Public Health	Guangdong Provincial Center for Diseases Control and Prevention	Min Kang, Jie Wu, Jing Lu, Tao Liu, Baisheng Li, Shujiang Mei, Feng Ruan, Lifeng Lin, Changwen Ke, Haojie Zhong, Yingtao Zhang, Lirong Zou, Xuguang Chen, Qi Zhu, Jianpeng Xiao, Jianxiang Geng, Zhe Liu, Jianxiong Hu, Weilin Zeng, Xing Li, Yuhuang Liao, Xiujuan Tang, Songjian Xiao, Ying Wang, Yingchao Song, Xue Zhuang, Lijun Liang, Guanhao He, Huihong Deng, Tie Song, Jianfeng He, Wenjun Ma
**EPI_ISL_406534**	hCoV-19/Foshan/20SF207/2020	Asia / China / Guangdong / Foshan	2020-01-22	Guangdong Provincial Center for Diseases Control and Prevention; Guangdong Provincial Public Health	Guangdong Provincial Center for Diseases Control and Prevention	Min Kang, Jie Wu, Jing Lu, Tao Liu, Baisheng Li, Shujiang Mei, Feng Ruan, Lifeng Lin, Changwen Ke, Haojie Zhong, Yingtao Zhang, Lirong Zou, Xuguang Chen, Qi Zhu, Jianpeng Xiao, Jianxiang Geng, Zhe Liu, Jianxiong Hu, Weilin Zeng, Xing Li, Yuhuang Liao, Xiujuan Tang, Songjian Xiao, Ying Wang, Yingchao Song, Xue Zhuang, Lijun Liang, Guanhao He, Huihong Deng, Tie Song, Jianfeng He, Wenjun Ma
**EPI_ISL_406535**	hCoV-19/Foshan/20SF210/2020	Asia / China / Guangdong / Foshan	2020-01-22	Guangdong Provincial Center for Diseases Control and Prevention; Guangdong Provincial Public Health	Guangdong Provincial Center for Diseases Control and Prevention	Min Kang, Jie Wu, Jing Lu, Tao Liu, Baisheng Li, Shujiang Mei, Feng Ruan, Lifeng Lin, Changwen Ke, Haojie Zhong, Yingtao Zhang, Lirong Zou, Xuguang Chen, Qi Zhu, Jianpeng Xiao, Jianxiang Geng, Zhe Liu, Jianxiong Hu, Weilin Zeng, Xing Li, Yuhuang Liao, Xiujuan Tang, Songjian Xiao, Ying Wang, Yingchao Song, Xue Zhuang, Lijun Liang, Guanhao He, Huihong Deng, Tie Song, Jianfeng He, Wenjun Ma

**EPI_ISL_406536**	hCoV-19/Foshan/20SF211/2020	Asia / China / Guangdong / Foshan	2020-01-22	Guangdong Provincial Center for Diseases Control and Prevention; Guangdong Provincial Public Health	Guangdong Provincial Center for Diseases Control and Prevention	Min Kang, Jie Wu, Jing Lu, Tao Liu, Baisheng Li, Shujiang Mei, Feng Ruan, Lifeng Lin, Changwen Ke, Haojie Zhong, Yingtao Zhang, Lirong Zou, Xuguang Chen, Qi Zhu, Jianpeng Xiao, Jianxiang Geng, Zhe Liu, Jianxiong Hu, Weilin Zeng, Xing Li, Yuhuang Liao, Xiujuan Tang, Songjian Xiao, Ying Wang, Yingchao Song, Xue Zhuang, Lijun Liang, Guanhao He, Huihong Deng, Tie Song, Jianfeng He, Wenjun Ma
**EPI_ISL_406538**	hCoV-19/Guangdong/20SF201/2020	Asia / China / Guangdong	2020-01-23	Guangdong Provincial Center for Diseases Control and Prevention;Guangdong Provincial Institute of Public Health	Guangdong Provincial Center for Diseases Control and Prevention	Min Kang, Jie Wu, Jing Lu, Tao Liu, Baisheng Li, Shujiang Mei, Feng Ruan, Lifeng Lin, Changwen Ke, Haojie Zhong, Yingtao Zhang, Lirong Zou, Xuguang Chen, Qi Zhu, Jianpeng Xiao, Jianxiang Geng, Zhe Liu, Jianxiong Hu, Weilin Zeng, Xing Li, Yuhuang Liao, Xiujuan Tang, Songjian Xiao, Ying Wang, Yingchao Song, Xue Zhuang, Lijun Liang, Guanhao He, Huihong Deng, Tie Song, Jianfeng He, Wenjun Ma
**EPI_ISL_406593**	hCoV-19/Shenzhen/SZTH-002/2020	Asia / China / Guangdong / Shenzhen	2020-01-13	Shenzhen Key Laboratory of Pathogen and Immunity, National Clinical Research Center for Infectious Disease, Shenzhen Third People's Hospital	Shenzhen Key Laboratory of Pathogen and Immunity, National Clinical Research Center for Infectious Disease, Shenzhen Third People's Hospital	Yang Yang, Chenguang Shen, Li Xing, Zhixiang Xu, Haixia Zheng, Yingxia Liu
**EPI_ISL_406594**	hCoV-19/Shenzhen/SZTH-003/2020	Asia / China / Guangdong / Shenzhen	2020-01-16	Shenzhen Key Laboratory of Pathogen and Immunity, National Clinical Research Center for Infectious Disease, Shenzhen Third People's Hospital	Shenzhen Key Laboratory of Pathogen and Immunity, National Clinical Research Center for Infectious Disease, Shenzhen Third People's Hospital	Yang Yang, Chenguang Shen, Li Xing, Zhixiang Xu, Haixia Zheng, Yingxia Liu
**EPI_ISL_406596**	hCoV-19/France/IDF0372/2020	Europe / France / Ile-de-France / Paris	2020-01-23	Department of Infectious and Tropical Diseases, Bichat Claude Bernard Hospital, Paris	National Reference Center for Viruses of Respiratory Infections, Institut Pasteur, Paris	Mélanie Albert, Marion Barbet, Sylvie Behillil, Méline Bizard, Angela Brisebarre, Flora Donati, Vincent Enouf, Maud Vanpeene, Sylvie van der Werf, Yazdan Yazdanpanah, Xavier Lescure.
**EPI_ISL_406597**	hCoV-19/France/IDF0373/2020	Europe / France / Ile-de-France / Paris	2020-01-23	Department of Infectious and Tropical Diseases, Bichat Claude Bernard Hospital, Paris	National Reference Center for Viruses of Respiratory Infections, Institut Pasteur, Paris	Mélanie Albert, Marion Barbet, Sylvie Behillil, Méline Bizard, Angela Brisebarre, Flora Donati, Vincent Enouf, Maud Vanpeene, Sylvie van der Werf, Yazdan Yazdanpanah, Xavier Lescure.
**EPI_ISL_406716**	hCoV-19/Wuhan/WHU01/2020	Asia / China / Hubei / Wuhan	2020-01-02	unknown	State Key Laboratory of Virology, Wuhan University	Chen,L., Liu,W., Zhang,Q., Xu,K., Ye,G., Wu,W., Sun,Z., Liu,F., Wu,K., Mei,Y., Zhang,W., Chen,Y., Li,Y., Shi,M., Lan,K. and Liu,Y.
**EPI_ISL_406717**	hCoV-19/Wuhan/WHU02/2020	Asia / China / Hubei / Wuhan	2020-01-02	unknown	State Key Laboratory of Virology, Wuhan University	Chen,L., Liu,W., Zhang,Q., Xu,K., Ye,G., Wu,W., Sun,Z., Liu,F., Wu,K., Mei,Y., Zhang,W., Chen,Y., Li,Y., Shi,M., Lan,K. and Liu,Y.
**EPI_ISL_406798**	hCoV-19/Wuhan/WH01/2019	Asia / China / Hubei / Wuhan	2019-12-26	General Hospital of Central Theater Command of People's Liberation Army of China	BGI & Institute of Microbiology, Chinese Academy of Sciences & Shandong First Medical University & Shandong Academy of Medical Sciences & General Hospital of Central Theater	Weijun Chen, Yuhai Bi, Weifeng Shi and Zhenhong Hu

					Command of People's Liberation Army of China	
**EPI_ISL_ 406800**	hCoV-19/Wuhan/WH03/2020	Asia / China / Hubei / Wuhan	2020-01-01	General Hospital of Central Theater Command of People's Liberation Army of China	BGI & Institute of Microbiology, Chinese Academy of Sciences & Shandong First Medical University & Shandong Academy of Medical Sciences & General Hospital of Central Theater Command of People's Liberation Army of China	Weijun Chen, Yuhai Bi, Weifeng Shi and Zhenhong Hu
**EPI_ISL_ 406801**	hCoV-19/Wuhan/WH04/2020	Asia / China / Hubei / Wuhan	2020-01-05	General Hospital of Central Theater Command of People's Liberation Army of China	BGI & Institute of Microbiology, Chinese Academy of Sciences & Shandong First Medical University & Shandong Academy of Medical Sciences & General Hospital of Central Theater Command of People's Liberation Army of China	Weijun Chen, Yuhai Bi, Weifeng Shi and Zhenhong Hu
**EPI_ISL_ 406844**	hCoV-19/Australia/VIC01/2020	Oceania / Australia / Victoria / Clayton	2020-01-25	Monash Medical Centre	Collaboration between the University of Melbourne at The Peter Doherty Institute for Infection and Immunity, and the Victorian Infectious Disease Reference Laboratory	Caly,L., Seemann,T., Schultz,M., Druce,J. and Taiaroa,G
**EPI_ISL_ 406862**	hCoV-19/Germany/BavPat1/2020	Europe / Germany / Bavaria / Munich	2020-01-28	Charité Universitätsmedizin Berlin, Institute of Virology; Institut für Mikrobiologie der Bundeswehr, Munich	Charité Universitätsmedizin Berlin, Institute of Virology	Victor M Corman, Julia Schneider, Talitha Veith, Barbara Mühlemann, Markus Antwerpen, Christian Drosten, Roman Wölfel
**EPI_ISL_ 406970**	hCoV-19/Hangzhou/HZ-1/2020	Asia / China / Zhejiang / Hangzhou	2020-01-20	Hangzhou Center for Disease and Control Microbiology Lab	Hangzhou Center for Disease and Control Microbiology Lab	Yu Hua, Wang Haoqiu, Li Jun, Yu Xinfeng
**EPI_ISL_ 406973**	hCoV-19/Singapore/1/2020	Asia / Singapore	2020-01-23	Singapore General Hospital	National Public Health Laboratory	Mak, TM; Octavia S; Chavatte JM; Zhou, ZY; Cui, L; Lin, RTP
**EPI_ISL_ 407071**	hCoV-19/England/01/2020	Europe / United Kingdom / England	2020-01-29	Respiratory Virus Unit, Microbiology Services Colindale, Public Health England	Respiratory Virus Unit, Microbiology Services Colindale, Public Health England	Monica Galiano, Shahjahan Miah, Richard Myers, Angie Lackenby, Omolola Akinbami, Tiina Talts, Leena Bhaw, Kirstin Edwards, Jonathan Hubb, Joanna Ellis, Maria Zambon
**EPI_ISL_ 407073**	hCoV-19/England/02/2020	Europe / United Kingdom / England	2020-01-29	Respiratory Virus Unit, Microbiology Services Colindale, Public Health England	Respiratory Virus Unit, Microbiology Services Colindale, Public Health England	Monica Galiano, Shahjahan Miah, Richard Myers, Angie Lackenby, Omolola Akinbami, Tiina Talts, Leena Bhaw, Kirstin Edwards, Jonathan Hubb, Joanna Ellis, Maria Zambon.

**EPI_ISL_407079**	hCoV-19/Finland/1/2020	Europe / Finland / Lapland	2020-01-29	Lapland Central Hospital	Department of Virology, University of Helsinki and Helsinki University Hospital, Helsinki, Finland	Teemu Smura, Suvi Kuivanen, Hannimari Kallio-Kokko, Olli Vapalahti
**EPI_ISL_407084**	hCoV-19/Japan/AI-I-004/2020	Asia / Japan / Aichi	2020-01-25	Department of Virology III, National Institute of Infectious Diseases	Pathogen Genomics Center, National Institute of Infectious Diseases	Tsuyoshi Sekizuka, Shutoku Matsuyama, Naganori Nao, Kazuya Shirato, Shinji Watanabe, Makoto Takeda, Makoto Kuroda
**EPI_ISL_407193**	hCoV-19/South Korea/KCDC03/2020	Asia / South Korea / Gyeonggi-do	2020-01-25	Korea Centers for Disease Control & Prevention (KCDC) Center for Laboratory Control of Infectious Diseases Division of Viral Diseases	Korea Centers for Disease Control & Prevention (KCDC) Center for Laboratory Control of Infectious Diseases Division of Viral Diseases	Jeong-Min Kim, Yoon-Seok Chung, Namjoo Lee, Mi-Seon Kim, SangHee Woo, Hye-Joon Jo, Sehee Park, Heui Man Kim, Myung Guk Han
**EPI_ISL_407313**	hCoV-19/Hangzhou/HZCDC0001/2020	Asia / China / Zhejiang / Hangzhou	2020-01-19	Hangzhou Center for Disease Control and Prevention	Hangzhou Center for Disease Control and Prevention	Jun Li, Haoqiu Wang, Hua Yu, Lingfeng Mao, Xinfen Yu, Zhou Sun, Qingxin Kong, Xin Qian, Shuchang Chen, Xuchu Wang
**EPI_ISL_407893**	hCoV-19/Australia/NSW01/2020	Oceania / Australia / New South Wales / Sydney	2020-01-24	Centre for Infectious Diseases and Microbiology Laboratory Services	NSW Health Pathology - Institute of Clinical Pathology and Medical Research; Westmead Hospital; University of Sydney	Eden J-S, Carter I, Rahman H, Holmes EC, Rockett R, O'Sullivan MV, Sintchenko V, Chen SC, Maddocks S, Kok J and Dwyer DE for the 2019-nCoV Study Group
**EPI_ISL_407894**	hCoV-19/Australia/QLD01/2020	Oceania / Australia / Queensland / Gold Coast	2020-01-28	Pathology Queensland	Public Health Virology Laboratory	Ben Huang, Alyssa Pyke, Amanda De Jong, Andrew Van Den Hurk, Carmel Taylor, David Warrilow, Doris Genge, Elisabeth Gamez, Glen Hewitson, Ian Maxwell Mackay, Inga Sultana, Jamie McMahon, Jean Barcelon, Judy Northill, Mitchell Finger, Natalie Simpson, Neelima Nair, Peter Burtonclay, Peter Moore, Sarah Wheatley, Sean Moody, Sonja Hall-Mendelin, Timothy Gardam, and Frederick Moore.
**EPI_ISL_407896**	hCoV-19/Australia/QLD02/2020	Oceania / Australia / Queensland / Gold Coast	2020-01-30	Pathology Queensland	Public Health Virology Laboratory	Ben Huang, Alyssa Pyke, Amanda De Jong, Andrew Van Den Hurk, Carmel Taylor, David Warrilow, Doris Genge, Elisabeth Gamez, Glen Hewitson, Ian Maxwell Mackay, Inga Sultana, Jamie McMahon, Jean Barcelon, Judy Northill, Mitchell Finger, Natalie Simpson, Neelima Nair, Peter Burtonclay, Peter Moore, Sarah Wheatley, Sean Moody, Sonja Hall-Mendelin, Timothy Gardam, and Frederick Moore.
**EPI_ISL_407976**	hCoV-19/Belgium/GHB-03021/2020	Europe / Belgium / Leuven	2020-02-03	KU Leuven, Clinical and Epidemiological Virology	KU Leuven, Clinical and Epidemiological Virology	Bert Vanmechelen, Elke Wollants, Annabel Rector, Els Keyaerts, Lies Laenen, Marc Van Ranst, and Piet Maes
**EPI_ISL_407987**	hCoV-19/Singapore/2/2020	Asia / Singapore	2020-01-25	Singapore General Hospital	Programme in Emerging Infectious Diseases, Duke-NUS Medical School	Danielle E Anderson, Martin Linster, Yan Zhuang, Jayanthi Jayakumar, Kian Sing Chan, Lynette LE Oon, Jenny GH Low, Yvonne CF Su, Linfa Wang, Gavin JD Smith
**EPI_ISL_407988**	hCoV-19/Singapore/3/2020	Asia / Singapore	2020-02-01	National Centre for Infectious Diseases	Programme in Emerging Infectious Diseases, Duke-NUS Medical School	Danielle E Anderson, Martin Linster, Yan Zhuang, Jayanthi Jayakumar, David CB Lye, Yee Sin Leo, Barnaby E Young, Yvonne CF Su, Linfa Wang, Gavin JD Smith
**EPI_ISL_408008**	hCoV-19/USA/CA3/2020	North America / USA / California	2020-01-29	California Department of Health	Pathogen Discovery, Respiratory Viruses Branch, Division of Viral Diseases, Centers	Krista Queen, Jing Zhang, Yan Li, Ying Tao, Anna Uehara, Clinton Paden, Xiaoyan Lu, Brian Lynch, Senthil Kumar K. Sakthivel, Brett L. Whitaker,

					for Disease Control and Prevention	Shifaq Kamili, Lijuan Wang, Janna' R. Murray, Susan I. Gerber, Stephen Lindstrom, Suxiang Tong
**EPI_ISL_ 408009**	hCoV-19/USA/ CA4/2020	North America / USA / California	2020-01-29	California Department of Health	Pathogen Discovery, Respiratory Viruses Branch, Division of Viral Diseases, Centers for Dieases Control and Prevention	Krista Queen, Jing Zhang, Yan Li, Ying Tao, Anna Uehara, Clinton Paden, Xiaoyan Lu, Brian Lynch, Senthil Kumar K. Sakthivel, Brett L. Whitaker, Shifaq Kamili, Lijuan Wang, Janna' R. Murray, Susan I. Gerber, Stephen Lindstrom, Suxiang Tong
**EPI_ISL_ 408010**	hCoV-19/USA/ CA5/2020	North America / USA / California	2020-01-29	California Department of Health	Pathogen Discovery, Respiratory Viruses Branch, Division of Viral Diseases, Centers for Dieases Control and Prevention	Ying Tao, Krista Queen, Jing Zhang, Yan Li, Anna Uehara, Clinton Paden, Xiaoyan Lu, Brian Lynch, Senthil Kumar K. Sakthivel, Brett L. Whitaker, Shifaq Kamili, Lijuan Wang, Janna' R. Murray, Susan I. Gerber, Stephen Lindstrom, Suxiang Tong
**EPI_ISL_ 408430**	hCoV-19/Franc e/IDF0515/202 0	Europe / France / Ile-de-Franc e / Paris	2020-01-29	Department of Infectious and Tropical Diseases, Bichat Claude Bernard Hospital, Paris	National Reference Center for Viruses of Respiratory Infections, Institut Pasteur, Paris	Mélanie Albert, Marion Barbet, Sylvie Behillil, Méline Bizard, Angela Brisebarre, Flora Donati, Vincent Enouf, Maud Vanpeene, Sylvie van der Werf, Yazdan Yazdanpanah, Xavier Lescure
**EPI_ISL_ 408431**	hCoV-19/Franc e/IDF0626/202 0	Europe / France / Ile-de-Franc e / Paris	2020-01-29	Sorbonne Université, Inserm et Assistance Publique-Hôpitau x de Paris (Pitié Salpétrière)	National Reference Center for Viruses of Respiratory Infections, Institut Pasteur, Paris	Mélanie Albert, Marion Barbet, Sylvie Behillil, Méline Bizard, Angela Brisebarre, Flora Donati, Vincent Enouf, Maud Vanpeene, Sylvie van der Werf, Sonia Burrel, Anne-Geneviève Marcelin, Vincent Calvez, David Boutolleau, Elise Klément, Valérie Pourcher, Eric Caumes.
**EPI_ISL_ 408478**	hCoV-19/Chon gqing/YC01/20 20	Asia / China / Chongqinq / Yongchuan	2020-01-21	Yongchuan District Center for Disease Control and Prevention	Chongqing Municipal Center for Disease Control and Prevention	Ye Sheng, Tang Yun, Ling Hua,Yu zhen,Chen Shuang,Tan ZhangPing, Su Kun, Li Qing, Tang Wenge, Rong Rong
**EPI_ISL_ 408479**	hCoV-19/Chon gqing/ZX01/20 20	Asia / China / Chongqing / Zhongxian	2020-01-23	Zhongxian Center for Disease Control and Prevention	Chongqing Municipal Center for Disease Control and Prevention	Ye Sheng, Tang Yun, Ling Hua, Zhang Hong, Yu zhen,Chen Shuang,Tan ZhangPing, Su Kun, Li Qin, Tang Wenge, Rong Rong
**EPI_ISL_ 408480**	hCoV-19/Yunn an/IVDC-YN-00 3/2020	Asia / China / Yunnan / Kunming	2020-01-17	National Institute for Viral Disease Control and Prevention, China CDC	National Institute for Viral Disease Control & Prevention, CCDC	Wenjie Tan，Xiaoqing Fu，Xiang Zhao，Wenling Wang， Peihua Niu，Roujian Lu,Yanhong Sun，Baoying Huang，Li Zhao，Fei Ye，Wenbo Xu，George F. Gao， Guizhen Wu
**EPI_ISL_ 408481**	hCoV-19/Chon gqing/IVDC-CQ -001/2020	Asia / China / Chongqing	2020-01-18	National Institute for Viral Disease Control and Prevention, China CDC	National Institute for Viral Disease Control & Prevention, CCDC	Wenjie Tan, Hengqin Wang, Xiang Zhao, Wenling Wang, Peihua Niu, Roujian Lu, Sheng Ye, Baoying Huang, Li Zhao, Fei Ye, Wenbo Xu, George F. Gao, Guizhen Wu
**EPI_ISL_ 408482**	hCoV-19/Shan dong/IVDC-SD- 001/2020	Asia / China / Shandong / Qingdao	2020-01-19	National Institute for Viral Disease Control and Prevention, China CDC	National Institute for Viral Disease Control & Prevention, CCDC	Wenjie Tan, Zhaoguo Wang, Xiang Zhao, Wenling Wang, Peihua Niu, Roujian Lu, Ti Liu, Baoying Huang, Li Zhao, Fei Ye, Wenbo Xu, George F. Gao, Guizhen Wu
**EPI_ISL_ 408484**	hCoV-19/Sichu an/IVDC-SC-00 1/2020	Asia / China / Sichuan / Chengdu	2020-01-15	National Institute for Viral Disease Control and Prevention, China CDC	National Institute for Viral Disease Control & Prevention, CCDC	Wenjie Tan, Jianan Xu, Wenling Wang, Peihua Niu, Roujian Lu, Huiping Yang, Xiang Zhao, Baoying Huang, Li Zhao, Fei Ye, Wenbo Xu, George F. Gao, Guizhen Wu
**EPI_ISL_ 408486**	hCoV-19/Jiang xi/IVDC-JX-002 /2020	Asia / China / Jiangxi / Pingxiang	2020-01-11	National Institute for Viral Disease Control and Prevention, China CDC	National Institute for Viral Disease Control & Prevention, CCDC	Wenjie Tan, Yong Shi, Wenling Wang, Peihua Niu, Roujian Lu, Jianxiong Li, Xiang Zhao, Baoying Huang, Li Zhao, Fei Ye, Wenbo Xu, George F. Gao, Guizhen Wu
**EPI_ISL_ 408488**	hCoV-19/Jiang su/IVDC-JS-001 /2020	Asia / China / Jiangsu / Huaian	2020-01-19	National Institute for Viral Disease Control and	National Institute for Viral Disease Control & Prevention, CCDC	Wenjie Tan, Shenjiao Wang, Wenling Wang, Peihua Niu, Roujian Lu, Kangchen Zhao, Xiang

				Prevention, China CDC		Zhao, Baoying Huang, Li Zhao, Fei Ye, Wenbo Xu, George F. Gao, Guizhen Wu
**EPI_ISL_ 408489**	hCoV-19/Taiwan/NTU01/2020	Asia / Taiwan / Taipei	2020-01-31	Department of Laboratory Medicine, National Taiwan University Hospital	Microbial Genomics Core Lab, National Taiwan University Centers of Genomic and Precision Medicine	Shiou-Hwei Yeh, You-Yu Lin, Ya-Yun Lai, Chiao-Ling Li, Shan-Chwen Chang, Pei-Jer Chen, Sui-Yuan Chang
**EPI_ISL_ 408514**	hCoV-19/env/ Wuhan/IVDC-HBF13-20/2020	Asia / China / Hubei / Wuhan	2020-01-01	Institute of Viral Disease Control and Prevention, China CDC	Institute of Viral Disease Control and Prevention, China CDC	William J. Liu, Peipei Liu, Xiang Zhao, Peihua Niu, Yingze Zhao, Wenwen Lei, Ziqian Xu, Shumei Zou, Wei Zhen, Beiwei Ye, Mengjie Yang, Weifeng Shi, Roujian Lu, Wenjie Tan, Zhixiao Chen, Yuchao Wu, Juan Song, Weimin Zhou, Dayan Wang, Jun Han, Wenbo Xu, George F. Gao, Guizhen Wu
**EPI_ISL_ 408515**	hCoV-19/env/ Wuhan/IVDC-HBF13-21/2020	Asia / China / Hubei / Wuhan	2020-01-01	Institute of Viral Disease Control and Prevention, China CDC	Institute of Viral Disease Control and Prevention, China CDC	William J. Liu, Peipei Liu, Xiang Zhao, Peihua Niu, Yingze Zhao, Wenwen Lei, Ziqian Xu, Shumei Zou, Wei Zhen, Beiwei Ye, Mengjie Yang, Weifeng Shi, Roujian Lu, Wenjie Tan, Zhixiao Chen, Yuchao Wu, Juan Song, Weimin Zhou, Dayan Wang, Jun Han, Wenbo Xu, George F. Gao, Guizhen Wu
**EPI_ISL_ 408665**	hCoV-19/Japan/TY-WK-012/2020	Asia / Japan / Tokyo	2020-01-29	Dept. of Virology III, National Institute of Infectious Diseases	Pathogen Genomics Center, National Institute of Infectious Diseases	Tsuyoshi Sekizuka, Shutoku Matsuyama, Naganori Nao, Kazuya Shirato, Makoto Takeda, Makoto Kuroda
**EPI_ISL_ 408666**	hCoV-19/Japan/TY-WK-501/2020	Asia / Japan / Tokyo	2020-01-31	Dept. of Virology III, National Institute of Infectious Diseases	Pathogen Genomics Center, National Institute of Infectious Diseases	Tsuyoshi Sekizuka, Shutoku Matsuyama, Naganori Nao, Kazuya Shirato, Makoto Takeda, Makoto Kuroda
**EPI_ISL_ 408667**	hCoV-19/Japan/TY-WK-521/2020	Asia / Japan / Tokyo	2020-01-31	Dept. of Virology III, National Institute of Infectious Diseases	Pathogen Genomics Center, National Institute of Infectious Diseases	Tsuyoshi Sekizuka, Shutoku Matsuyama, Naganori Nao, Kazuya Shirato, Makoto Takeda, Makoto Kuroda
**EPI_ISL_ 408668**	hCoV-19/Vietnam/VR03-38142/2020	Asia / Vietnam / Thanh Hoa	2020-01-24	National Influenza Center - National Institute of Hygiene and Epidemiology (NIHE)	National Influenza Center - National Institute of Hygiene and Epidemiology (NIHE)	Ung Thi Hong Trang, Hoang Vu Mai Phuong, Nguyen Le Khanh Hang, Nguyen Vu Son, Le Thi Thanh, Vuong Duc Cuong, Nguyen Phuong Anh, Pham Thi Hien, Tran Thu Huong, Le Thi Quynh Mai,
**EPI_ISL_ 408669**	hCoV-19/Japan/KY-V-029/2020	Asia / Japan / Kyoto	2020-01-29	Dept. of Virology III, National Institute of Infectious Diseases	Pathogen Genomics Center, National Institute of Infectious Diseases	Tsuyoshi Sekizuka, Shutoku Matsuyama, Naganori Nao, Kazuya Shirato, Makoto Takeda, Makoto Kuroda
**EPI_ISL_ 408670**	hCoV-19/USA/ WI1/2020	North America / USA / Wisconsin	2020-01-31	Wisconsin Department of Health Services	Pathogen Discovery, Respiratory Viruses Branch, Division of Viral Diseases, Centers for Dieases Control and Prevention	Jing Zhang, Anna Uehara, Krista Queen, Yan Li, Ying Tao, Clinton R. Paden, Xiaoyan Lu, Brian Lynch, Senthil Kumar K. Sakthivel, Brett L. Whitaker, Shifaq Kamili, Lijuan Wang, Janna' R. Murray, Susan I. Gerber, Stephen Lindstrom, Suxiang Tong
**EPI_ISL_ 408976**	hCoV-19/Australia/NSW02/2020	Oceania / Australia / New South Wales / Sydney	2020-01-22	Centre for Infectious Diseases and Microbiology Laboratory Services	NSW Health Pathology - Institute of Clinical Pathology and Medical Research; Westmead Hospital; University of Sydney	Rockett R, Sadsad R, Eden J-S, Carter I, Rahman H, Holmes EC, O'Sullivan MV, Sintchenko V, Chen SC, Maddocks S, Kok J and Dwyer DE for the 2019-nCoV Study Group*
**EPI_ISL_ 408977**	hCoV-19/Australia/NSW03/2020	Oceania / Australia / New South	2020-01-25	Serology, Virology and OTDS Laboratories (SAViD), NSW	NSW Health Pathology - Institute of Clinical Pathology and Medical Research; Centre for	Eden J-S, Carter I, Rahman H, Rawlinson W, Holmes EC, Rockett R, O'Sullivan MV, Sintchenko V, Chen SC, Maddocks S, Kok J and Dwyer DE for the 2019-nCoV Study Group*

		Wales / Sydney		Health Pathology Randwick	Infectious Diseases and Microbiology Laboratory Services; Westmead Hospital; University of Sydney	
**EPI_ISL_408978**	hCoV-19/Wuhan/WH05/2020	Asia / China / Hubei / Wuhan	2020-02-07	Wuhan Fourth Hospital	Beijing Genomics Institute (BGI)	Weijun Chen
**EPI_ISL_409067**	hCoV-19/USA/MA1/2020	North America / USA / Massachusetts	2020-01-29	Massachusetts Department of Public Health	Pathogen Discovery, Respiratory Viruses Branch, Division of Viral Diseases, Centers for Dieases Control and Prevention	Clinton R. Paden, Jing Zhang, Krista Queen, Yan Li, Ying Tao, Anna Uehara, Xiaoyan Lu, Brian Lynch, Senthil Kumar K. Sakthivel, Brett L. Whitaker, Shifaq Kamili, Lijuan Wang, Janna' R. Murray, Susan I. Gerber, Stephen Lindstrom, Suxiang Tong
**EPI_ISL_410044**	hCoV-19/USA/CA6/2020	North America / USA / California	2020-01-27	California Department of Public Health	Pathogen Discovery, Respiratory Viruses Branch, Division of Viral Diseases, Centers for Dieases Control and Prevention	Jing Zhang, Krista Queen, Yan Li, Ying Tao, Anna Uehara, Clinton R. Paden, Xiaoyan Lu, Brian Lynch, Senthil Kumar K. Sakthivel, Brett L. Whitaker, Shifaq Kamili, Lijuan Wang, Janna' R. Murray, Susan I. Gerber, Stephen Lindstrom, Suxiang Tong
**EPI_ISL_410045**	hCoV-19/USA/IL2/2020	North America / USA / Illinois	2020-01-28	IL Department of Public Health Chicago Laboratory	Pathogen Discovery, Respiratory Viruses Branch, Division of Viral Diseases, Centers for Dieases Control and Prevention	Yan Li, Jing Zhang, Krista Queen, Ying Tao, Anna Uehara, Clinton R. Paden, Xiaoyan Lu, Brian Lynch, Senthil Kumar K. Sakthivel, Brett L. Whitaker, Shifaq Kamili, Lijuan Wang, Janna' R. Murray, Susan I. Gerber, Stephen Lindstrom, Suxiang Tong
**EPI_ISL_410218**	hCoV-19/Taiwan/NTU02/2020	Asia / Taiwan / Taipei	2020-02-05	Department of Laboratory Medicine, National Taiwan University Hospital	Microbial Genomics Core Lab, National Taiwan University Centers of Genomic and Precision Medicine	Shiou-Hwei Yeh, You-Yu Lin, Ya-Yun Lai, Chiao-Ling Li, Shan-Chwen Chang, Pei-Jer Chen, Sui-Yuan Chang
**EPI_ISL_410301**	hCoV-19/Nepal/61/2020	Asia / Nepal / Kathmandu	2020-01-13	National Influenza Centre, National Public Health Laboratory, Kathmandu, Nepal	The University of Hong Kong	Ranjit Sah, Runa Jha, Daniel Chu, Haogao Gu, Malik Peiris, Anup Bastola, Alfonso J. Rodriguez-Morales, Bibek Kumar Lal, Basu Dev Pandey, Leo Poon
**EPI_ISL_410486**	hCoV-19/France/RA739/2020	Europe / France / Rhone-Alpes / Contamines	2020-02-08	CNR Virus des Infections Respiratoires - France SUD	CNR Virus des Infections Respiratoires - France SUD	Bal, Antonin; Destras, Gregory; Gaymard, Alexandre; Bouscambert-Duchamp, Maude; Cheynet, Valérie; Brengel-Pesce, Karen; Morfin-Sherpa, Florence; Valette, Martine; Josset, Laurence; Lina, Bruno.
**EPI_ISL_410531**	hCoV-19/Japan/NA-20-05-1/2020	Asia / Japan / Nara	2020-01-25	Dept. of Pathology, National Institute of Infectious Diseases	Pathogen Genomics Center, National Institute of Infectious Diseases	Tsuyoshi Sekizuka, Harutaka Katano, Shutoku Matsuyama, Naganori Nao, Kazuya Shirato, Motoi Suzuki, Hideki Hasegawa, Takaji Wakita, Makoto Takeda, Tadaki Suzuki, Makoto Kuroda
**EPI_ISL_410532**	hCoV-19/Japan/OS-20-07-1/2020	Asia / Japan / Osaka	2020-01-23	Dept. of Pathology, National Institute of Infectious Diseases	Pathogen Genomics Center, National Institute of Infectious Diseases	Tsuyoshi Sekizuka, Harutaka Katano, Shutoku Matsuyama, Naganori Nao, Kazuya Shirato, Motoi Suzuki, Hideki Hasegawa, Takaji Wakita, Makoto Takeda, Tadaki Suzuki, Makoto Kuroda
**EPI_ISL_410535**	hCoV-19/Singapore/4/2020	Asia / Singapore	2020-02-03	National Centre for Infectious Diseases	Programme in Emerging Infectious Diseases, Duke-NUS Medical School	Danielle E Anderson, Martin Linster, Yan Zhuang, Jayanthi Jayakumar, David CB Lye, Yee Sin Leo, Barnaby E Young, Yvonne CF Su, Gavin JD Smith
**EPI_ISL_410536**	hCoV-19/Singapore/5/2020	Asia / Singapore	2020-02-06	Singapore General Hospital, Molecular Laboratory,	Programme in Emerging Infectious Diseases, Duke-NUS Medical School	Danielle E Anderson, Martin Linster, Yan Zhuang, Jayanthi Jayakumar, Kian Sing Chan, Lynette LE Oon, Shirin Kalimuddin, Jenny GH Low, Yvonne CF Su, Gavin JD Smith

				Division of Pathology		
**EPI_ISL_ 410537**	hCoV-19/Singapore/6/2020	Asia / Singapore	2020-02-09	Singapore General Hospital, Molecular Laboratory, Division of Pathology	Programme in Emerging Infectious Diseases, Duke-NUS Medical School	Danielle E Anderson, Martin Linster, Yan Zhuang, Jayanthi Jayakumar, Kian Sing Chan, Lynette LE Oon, Shirin Kalimuddin, Jenny GH Low, Yvonne CF Su, Gavin JD Smith
**EPI_ISL_ 410545**	hCoV-19/Italy/INMI1-isl/2020	Europe / Italy / Rome	2020-01-29	INMI Lazzaro Spallanzani IRCCS	Laboratory of Virology, INMI Lazzaro Spallanzani IRCCS	Maria R. Capobianchi, Cesare E. M. Gruber, Martina Rueca, Barbara Bartolini, Francesco Messina, Emanuela Giombini, Francesca Colavita, Concetta Castilletti, Eleonora Lalle, Fabrizio Carletti, Emanuele Nicastri, Giuseppe Ippolito.
**EPI_ISL_ 410713**	hCoV-19/Singapore/7/2020	Asia / Singapore	2020-01-27	National Public Health Laboratory, National Centre for Infectious Diseases	National Public Health Laboratory, National Centre for Infectious Diseases	Octavia S, Mak TM, Cui L, Lin RTP
**EPI_ISL_ 410714**	hCoV-19/Singapore/8/2020	Asia / Singapore	2020-02-03	National Public Health Laboratory, National Centre for Infectious Diseases	National Public Health Laboratory, National Centre for Infectious Diseases	Octavia S, Mak TM, Cui L, Lin RTP
**EPI_ISL_ 410715**	hCoV-19/Singapore/9/2020	Asia / Singapore	2020-02-04	National Public Health Laboratory, National Centre for Infectious Diseases	National Public Health Laboratory, National Centre for Infectious Diseases	Octavia S, Mak TM, Cui L, Lin RTP
**EPI_ISL_ 410716**	hCoV-19/Singapore/10/2020	Asia / Singapore	2020-02-04	National Public Health Laboratory, National Centre for Infectious Diseases	National Centre for Infectious Diseases, National Centre for Infectious Diseases	Octavia S, Mak TM, Cui L, Lin RTP
**EPI_ISL_ 410717**	hCoV-19/Australia/QLD03/2020	Oceania / Australia / Queensland / Gold Coast	2020-02-05	Pathology Queensland	Public Health Virology Laboratory	Ben Huang, Alyssa Pyke, Amanda De Jong, Andrew Van Den Hurk, Carmel Taylor, David Warrilow, Doris Genge, Elisabeth Gamez, Glen Hewitson, Ian Maxwell Mackay, Inga Sultana, Jamie McMahon, Jean Barcelon, Judy Northill, Mitchell Finger, Natalie Simpson, Neelima Nair, Peter Burtonclay, Peter Moore, Sarah Wheatley, Sean Moody, Sonja Hall-Mendelin, Timothy Gardam, and Frederick Moore.
**EPI_ISL_ 410718**	hCoV-19/Australia/QLD04/2020	Oceania / Australia / Queensland / Gold Coast	2020-02-05	Pathology Queensland	Public Health Virology Laboratory	Ben Huang, Alyssa Pyke, Amanda De Jong, Andrew Van Den Hurk, Carmel Taylor, David Warrilow, Doris Genge, Elisabeth Gamez, Glen Hewitson, Ian Maxwell Mackay, Inga Sultana, Jamie McMahon, Jean Barcelon, Judy Northill, Mitchell Finger, Natalie Simpson, Neelima Nair, Peter Burtonclay, Peter Moore, Sarah Wheatley, Sean Moody, Sonja Hall-Mendelin, Timothy Gardam, and Frederick Moore.
**EPI_ISL_ 410719**	hCoV-19/Singapore/11/2020	Asia / Singapore	2020-02-02	National Public Health Laboratory	National Public Health Laboratory	Octavia S, Mak TM, Cui L, Lin RTP
**EPI_ISL_ 411060**	hCoV-19/Fujian/8/2020	Asia / China / Fujian	2020-01-21	Fujian Center for Disease Control and Prevention	Fujian Center for Disease Control and Prevention	Chen Wei, Zhang Yanhua, He Wenxiang, Weng Yuwei

**EPI_ISL_411066**	hCoV-19/Fujian/13/2020	Asia / China / Fujian	2020-01-22	Fujian Center for Disease Control and Prevention	Fujian Center for Disease Control and Prevention	Chen Wei, Zhang Yanhua, He Wenxiang, Weng Yuwei
**EPI_ISL_411218**	hCoV-19/France/IDF0571/2020	Europe / France / Ile-de-France / Paris	2020-02-02	Department of Infectious and Tropical Diseases, Bichat Claude Bernard Hospital, Paris	Laboratoire Virpath, CIRI U111, UCBL1, INSERM, CNRS, ENS Lyon	Olivier Terrier, Aurélien Traversier, Julien Fouret, Yazdan Yazdanpanah, Xavier Lescure, Catherine Legras-Lachuer, Alexandre Gaymard, Bruno Lina, Manuel Rosa-Calatrava
**EPI_ISL_411219**	hCoV-19/France/IDF0386-islP1/2020	Europe / France / Ile-de-France / Paris	2020-01-28	Department of Infectious and Tropical Diseases, Bichat Claude Bernard Hospital, Paris	Laboratoire Virpath, CIRI U111, UCBL1, INSERM, CNRS, ENS Lyon	Olivier Terrier, Aurélien Traversier, Julien Fouret, Yazdan Yazdanpanah, Xavier Lescure, Alexandre Gaymard, Bruno Lina, Manuel Rosa-Calatrava
**EPI_ISL_411902**	hCoV-19/Cambodia/0012/2020	Asia / Cambodia / Sihanoukville	2020-01-27	Virology Unit, Institut Pasteur du Cambodge.	Virology Unit, Institut Pasteur du Cambodge (Sequencing done by: Jessica E Manning/Jennifer A Bohl at Malaria and Vector Research Research Laboratory, National Institute of Allergy and Infectious Diseases and Vida Ahyong from Chan-Zuckerberg Biohub)	Erik A Karlsson, Jennifer A Bohl, Vida Ahyong, Veasna Duong, Philippe Dussart, Jessica E Manning.
**EPI_ISL_411950**	hCoV-19/Jiangsu/JS01/2020	Asia / China / Jiangsu	2020-01-23	NHC Key laboratory of Enteric Pathogenic Microbiology, Institute of Pathogenic Microbiology	Jiangsu Provincial Center for Disease Control & Prevention	Lunbiao Cui,Kangchen Zhao,Xiaojuan Zhu,Yiyue Ge,Tao Wu,Bin Wu,Yin Chen,Fengcai Zhu,Baoli Zhu,Ming Wu
**EPI_ISL_411951**	hCoV-19/Sweden/01/2020	Europe / Sweden	2020-02-07	unknown	Unit for Laboratory Development and Technology Transfer, Public Health Agency of Sweden	Bengner,M., Palmerus,M., Lindsjo,O., Lind Karlberg,M., Monteil,V., Appelberg,S., Brave,A., Muradrasoli,S. and Tegmark-Wisell,K.
**EPI_ISL_411952**	hCoV-19/Jiangsu/JS02/2020	Asia / China / Jiangsu	2020-01-24	NHC Key laboratory of Enteric Pathogenic Microbiology, Institute of Pathogenic Microbiology	Jiangsu Provincial Center for Disease Control & Prevention	Kangchen Zhao, Xiaojuan Zhu, Lunbiao Cui, Tao Wu, Yiyue Ge, Bin Wu, Yin Chen, Fengcai Zhu, Baoli Zhu, Ming Wu
**EPI_ISL_411953**	hCoV-19/Jiangsu/JS03/2020	Asia / China / Jiangsu	2020-01-24	NHC Key laboratory of Enteric Pathogenic Microbiology, Institute of Pathogenic Microbiology	Jiangsu Provincial Center for Disease Control & Prevention	Kangchen Zhao, Xiaojuan Zhu, Lunbiao Cui, Tao Wu, Yiyue Ge, Bin Wu, Yin Chen, Fengcai Zhu, Baoli Zhu, Ming Wu
**EPI_ISL_411954**	hCoV-19/USA/CA7/2020	North America / USA / California	2020-02-06	California Department of Public Health	Pathogen Discovery, Respiratory Viruses Branch, Division of Viral Diseases, Centers for Dieases Control and Prevention	Krista Queen, Anna Uehara, Jing Zhang, Yan Li, Ying Tao, Clinton R. Paden, Haibin Wang, Shifaq Kamili, Xiaoyan Lu, Brian Lynch, Senthil Kumar K. Sakthivel, Brett L. Whitaker, Lijuan Wang, Janna' R. Murray, Susan I. Gerber, Stephen Lindstrom, Suxiang Tong

**EPI_ISL_ 411955**	hCoV-19/USA/ CA8/2020	North America / USA / California	2020-02-10	California Department of Public Health	Pathogen Discovery, Respiratory Viruses Branch, Division of Viral Diseases, Centers for Dieases Control and Prevention	Krista Queen, Anna Uehara, Jing Zhang, Yan Li, Ying Tao, Clinton R. Paden, Haibin Wang, Shifaq Kamili, Xiaoyan Lu, Brian Lynch, Senthil Kumar K. Sakthivel, Brett L. Whitaker, Lijuan Wang, Janna' R. Murray, Susan I. Gerber, Stephen Lindstrom, Suxiang Tong
**EPI_ISL_ 411956**	hCoV-19/USA/ TX1/2020	North America / USA / Texas	2020-02-11	Texas Department of State Health Services	Pathogen Discovery, Respiratory Viruses Branch, Division of Viral Diseases, Centers for Dieases Control and Prevention	Krista Queen, Anna Uehara, Jing Zhang, Yan Li, Ying Tao, Clinton R. Paden, Haibin Wang, Shifaq Kamili, Xiaoyan Lu, Brian Lynch, Senthil Kumar K. Sakthivel, Brett L. Whitaker, Lijuan Wang, Janna' R. Murray, Susan I. Gerber, Stephen Lindstrom, Suxiang Tong
**EPI_ISL_ 412026**	hCoV-19/Hefei /2/2020	Asia / China / Anhui / Hefei	2020-02-23	Second Hospital of Anhui Medical University	Second Hospital of Anhui Medical University	Changtai Wang, Zhongping Liua, Zixiang Chen, Xin Huang, Mengyuan Xua, Tengfei He, Mengji Lu, Zhenhua Zhang
**EPI_ISL_ 412028**	hCoV-19/Hong Kong/VM2000 1061/2020	Asia / Hong Kong	2020-01-22	Hong Kong Department of Health	School of Public Health, The University of Hon g Kong	Dominic N.C. Tsang, Daniel K.W. Chu, Leo L.M. Poon, Malik Peiris
**EPI_ISL_ 412029**	hCoV-19/Hong Kong/VB20024 950/2020	Asia / Hong Kong	2020-01-30	Hong Kong Department of Health	The University of Hong Kong	Dominic N.C. Tsang, Daniel K.W. Chu, Leo L.M. Poon, Malik Peiris
**EPI_ISL_ 412030**	hCoV-19/Hong Kong/VB20026 565/2020	Asia / Hong Kong	2020-02-01	Hong Kong Department of Health	School of Public Health, The University of Hon g Kong	Dominic N.C. Tsang, Daniel K.W. Chu, Leo L.M. Poon, Malik Peiris
**EPI_ISL_ 412116**	hCoV-19/Engla nd/09c/2020	Europe / United Kingdom / England	2020-02-09	Respiratory Virus Unit, Microbiology Services Colindale, Public Health England	Respiratory Virus Unit, Microbiology Services Colindale, Public Health England	Monica Galiano, Shahjahan Miah, Angie Lackenby, Omolola Akinbami, Tiina Talts, Leena Bhaw, Richard Myers, Steven Platt, Kirstin Edwards, Jonathan Hubb, Joanna Ellis, Maria Zambon
**EPI_ISL_ 412459**	hCoV-19/Jingz hou/HBCDC-H B-01/2020	Asia / China / Hubei / Jingzhou	2020-01-08	Jingzhou Center for Disease Control and Prevention	Hubei Provincial Center for Disease Control and Prevention	Bin Fang, Xiang Li, Xiao Yu, Linlin Liu, Bo Yang, Faxian Zhan, Guojun Ye, Xixiang Huo, Junqiang Xu, Bo Yu, Kun Cai, Jing Li, Maoyi Chen,Jie Hu, Chunlin Mao, Yongzhong Jiang.
**EPI_ISL_ 412862**	hCoV-19/USA/ CA9/2020	North America / USA / California / Solano	2020-02-23	California Department of Public Health	Pathogen Discovery, Respiratory Viruses Branch, Division of Viral Diseases, Centers for Disease Control and Prevention	Krista Queen, Anna Uehara, Jing Zhang, Yan Li, Ying Tao, Clinton R. Paden, Haibin Wang, Shifaq Kamili, Xiaoyan Lu, Brian Lynch, Senthil Kumar K. Sakthivel, Brett L. Whitaker, Lijuan Wang, Janna' R. Murray, Jasmine Padilla, Justin Lee, Susan I. Gerber, Stephen Lindstrom, Suxiang Tong
**EPI_ISL_ 412869**	hCoV-19/South Korea/KCDC05 /2020	Asia / South Korea /Seoul	2020-01-30	Division of Viral Diseases, Center for Laboratory Control of Infectious Diseases, Korea Centers for Diseases Control and Prevention	Division of Viral Diseases, Center for Laboratory Control of Infectious Diseases, Korea Centers for Diseases Control and Prevention	Jeong-Min Kim, Yoon-Seok Chung, Namjoo Lee, Mi-Seon Kim, Sang Hee Woo, Hye-Jun Jo, Sehee Park, Heui Man Kim, Myung Guk Han
**EPI_ISL_ 412870**	hCoV-19/South Korea/KCDC06 /2020	Asia / South Korea/ Seoul	2020-01-30	Division of Viral Diseases, Center for Laboratory Control of Infectious Diseases, Korea Centers for Diseases Control and Prevention	Division of Viral Diseases, Center for Laboratory Control of Infectious Diseases, Korea Centers for Diseases Control and Prevention	Jeong-Min Kim, Yoon-Seok Chung, Namjoo Lee, Mi-Seon Kim, Sang Hee Woo, Hye-Jun Jo, Sehee Park, Heui Man Kim, Myung Guk Han
**EPI_ISL_ 412871**	hCoV-19/South Korea/KCDC07 /2020	Asia / South Korea / Seoul	2020-01-31	Division of Viral Diseases, Center for Laboratory Control of	Division of Viral Diseases, Center for Laboratory Control of Infectious Diseases,	Jeong-Min Kim, Yoon-Seok Chung, Namjoo Lee, Mi-Seon Kim, Sang Hee Woo, Hye-Jun Jo, Sehee Park, Heui Man Kim, Myung Guk Han

				Infectious Diseases, Korea Centers for Diseases Control and Prevention	Korea Centers for Diseases Control and Prevention	
**EPI_ISL_412872**	hCoV-19/South Korea/KCDC12/2020	Asia / South Korea / Gyeonggi-do	2020-02-01	Division of Viral Diseases, Center for Laboratory Control of Infectious Diseases, Korea Centers for Diseases Control and Prevention	Division of Viral Diseases, Center for Laboratory Control of Infectious Diseases, Korea Centers for Diseases Control and Prevention	Jeong-Min Kim, Yoon-Seok Chung, Namjoo Lee, Mi-Seon Kim, Sang Hee Woo, Hye-Jun Jo, Sehee Park, Heui Man Kim, Myung Guk Han
**EPI_ISL_412873**	hCoV-19/South Korea/KCDC24/2020	Asia / South Korea / Chungcheongnam-do	2020-02-06	Division of Viral Diseases, Center for Laboratory Control of Infectious Diseases, Korea Centers for Diseases Control and Prevention	Division of Viral Diseases, Center for Laboratory Control of Infectious Diseases, Korea Centers for Diseases Control and Prevention	Jeong-Min Kim, Yoon-Seok Chung, Namjoo Lee, Mi-Seon Kim, Sang Hee Woo, Hye-Jun Jo, Sehee Park, Heui Man Kim, Myung Guk Han
**EPI_ISL_412898**	hCoV-19/Wuhan/HBCDC-HB-02/2019	Asia / China / Hubei / Wuhan	2019-12-30	Wuhan Jinyintan Hospital	Hubei Provincial Center for Disease Control and Prevention	Bin Fang, Xiang Li, Xiao Yu, Linlin Liu, Bo Yang, Faxian Zhan, Guojun Ye, Xixiang Huo, Junqiang Xu, Bo Yu, Kun Cai, Jing Li, Yongzhong Jiang.
**EPI_ISL_412899**	hCoV-19/Wuhan/HBCDC-HB-03/2019	Asia / China / Hubei / Wuhan	2019-12-30	Wuhan Jinyintan Hospital	Hubei Provincial Center for Disease Control and Prevention	Bin Fang, Xiang Li, Xiao Yu, Linlin Liu, Bo Yang, Faxian Zhan, Guojun Ye, Xixiang Huo, Junqiang Xu, Bo Yu, Kun Cai, Jing Li, Yongzhong Jiang.
**EPI_ISL_412912**	hCoV-19/Germany/Baden-Wuerttemberg-1/2020	Europe / Germany / Baden-Wuerttemberg	2020-02-25	State Health Office Baden-Wuerttemberg	Charité Universitätsmedizin Berlin, Institute of Virology	Victor M Corman, Julia Schneider, Barbara Mühlemann, Talitha Veith, Jörn Beheim-Schwarzbach, Terry Jones, Rainer Oehme, Silke Fischer, Christian Drosten
**EPI_ISL_412964**	hCoV-19/Brazil/SPBR-01/2020	South America / Brazil / Sao Paulo / Sao Paulo	2020-02-25	Hospital Israelita Albert Einstein	Instituto Adolfo Lutz Interdisciplinary Procedures Center Strategic Laboratory	Jaqueline Goes de Jesus, Claudio Tavares Sacchi, Daniela Bernardes Borges da Silva, Ingra Morales Claro, Flávia Cristina da Silva Sales, Claudia Regina Gonçalves, Joshua Quick, Maria do Carmo, Sampaio Tavares Timenetsky, Nicholas James Loman, Andrew Rambaut, Ester Cerdeira Sabino, Nuno Rodrigues Faria
**EPI_ISL_412965**	hCoV-19/Canada/BC_37_0-2/2020	North America / Canada / British Columbia	2020-02-16	BCCDC Public Health Laboratory	BCCDC Public Health Laboratory	Harrigan, Prystajecky, Krajden, Lee, Kamelian, Lapointe, Choi, Hoang, Sekirov, Levett, Tyson, Loman, Quick, Li, Gilmour
**EPI_ISL_412966**	hCoV-19/China/IQTC01/2020	Asia / China / Guangdong / Guangzhou	2020-02-05	unknown	Technology Centre, Guangzhou Customs	Shi,Y., Sun,J., Zheng,K., Huang,J. and Zhao,J.
**EPI_ISL_412967**	hCoV-19/China/IQTC02/2020	Asia / China / Guangzhou	2020-01-29	unknown	Technology Centre, Guangzhou Customs	Shi,Y., Zheng,K., Sun,J., Huang,J., Zhu,A., Zhuang,Z., Dai,J., Chen,Z., Sun,F., Zhang,Z., Li,X. and Wang,Y.
**EPI_ISL_412968**	hCoV-19/Japan/Hu_DP_Kng_19-020/2020	Asia / Japan	2020-02-10	unknown	Takayuki Hishiki Kanagawa Prefectural Institute of Public Health, Department of Microbiology	Hishiki,T., Suzuki,R., Sakuragi,J., Usui,K., Tanaka,Y., Kawai,J., Kogo,Y., Matsuki,Y., An,T., Hayashizaki,Y. and Takasaki,T.

**EPI_ISL_412969**	hCoV-19/Japan/Hu_DP_Kng_19-027/2020	Asia / Japan	2020-02-10	unknown	Takayuki Hishiki Kanagawa Prefectural Institute of Public Health, Department of Microbiology	Hishiki,T., Suzuki,R., Sakuragi,J., Usui,K., Tanaka,Y., Kawai,J., Kogo,Y., Matsuki,Y., An,T., Hayashizaki,Y. and Takasaki,T.
**EPI_ISL_412970**	hCoV-19/USA/WA2/2020	North America / USA / Washington / Snohomish County	2020-02-24	Washington State Department of Health	Seattle Flu Study	Helen Chu, Michael Boeckh, Janet Englund, Michael Famulare, Barry Lutz, Deborah Nickerson, Mark Rieder, Lea Starita, Matthew Thompson, Jay Shendure, and Trevor Bedford
**EPI_ISL_412971**	hCoV-19/Finland/FIN-25/2020	Europe / Finland / Helsinki	2020-02-25	HUS Diagnostiikkakeskus, Hallinto	Department of Virology Faculty of Medicine, Medicum University of Helsinki	Teemu Smura, Suvi Kuivanen, Hannimari Kallio-Kokko, Olli Vapalahti
**EPI_ISL_412972**	hCoV-19/Mexico/CDMX-InDRE_01/2020	North America / Mexico / Mexico City	2020-02-27	Instituto Nacional de Enfermedades Respiratorias	Instituto de Diagnostico y Referencia Epidemiologicos (INDRE)	Ramirez-Gonzalez Ernesto, Garces-Ayala Fabiola, Araiza-Rodriguez Adnan, Mendieta-Condado Edgar, Rodriguez-Maldonado Abril, Wong-Arambula Claudia, Vazquez-Perez Joel, Martinez Arturo, Boukadida Celia, Munoz-Medina Esteban, Sanchez Alejandro, Isa Pavel, Taboada Blanca, Lopez Susana, Arias Carlos, Barrera-Badillo Gisela, Hernandez-Rivas Lucia, Lopez-Martinez Irma
**EPI_ISL_412973**	hCoV-19/Italy/CDG1/2020	Europe / Italy / Lombardy	2020-02-20	Department of Infectious Diseases, Istituto Superiore di Sanità, Roma, Italy	Virology Laboratory, Scientific Department, Army Medical Center	Paola Stefanelli, Stefano Fiore, Antonella Marchi, Eleonora Benedetti, Concetta Fabiani, Giovanni Faggioni, Antonella Fortunato, Riccardo De Santis, Silvia Fillo, Anna Anselmo, Andrea Ciammaruconi, Stefano Palomba, Florigio Lista
**EPI_ISL_412974**	hCoV-19/Italy/SPL1/2020	Europe / Italy / Rome	2020-01-29	Department of Infectious Diseases, Istituto Superiore di Sanità, Rome, Italy	Virology Laboratory, Scientific Department, Army Medical Center	Paola Stefanelli, Stefano Fiore, Antonella Marchi, Eleonora Benedetti, Concetta Fabiani, Giovanni Faggioni, Antonella Fortunato, Silvia Fillo, Riccardo De Santis, Andrea Ciammaruconi, Giancarlo Petralito, Filippo Molinari, Florigio Lista
**EPI_ISL_412975**	hCoV-19/Australia/NSW05/2020	Oceania / Australia / New South Wales / Sydney	2020-02-28	Centre for Infectious Diseases and Microbiology Laboratory Services	NSW Health Pathology - Institute of Clinical Pathology and Medical Research; Westmead Hospital; University of Sydney	Eden J-S, Carter I, Rahman H, Holmes EC, Rockett R, O'Sullivan MV, Sintchenko V, Chen SC, Maddocks S, Kok J and Dwyer DE for the 2019-nCoV Study Group
**EPI_ISL_412978**	hCoV-19/Wuhan/HBCDC-HB-02/2020	Asia / China / Hubei / Wuhan	2020-01-17	The Central Hospital Of Wuhan	Hubei Provincial Center for Disease Control and Prevention	Bin Fang, Xiang Li, Xiao Yu, Linlin Liu, Bo Yang, Faxian Zhan, Guojun Ye, Xixiang Huo, Junqiang Xu, Bo Yu, Kun Cai, Jing Li, Yongzhong Jiang.
**EPI_ISL_412979**	hCoV-19/Wuhan/HBCDC-HB-03/2020	Asia / China / Hubei / Wuhan	2020-01-18	Union Hospital of Tongji Medical College, Huazhong University of Science and Technology	Hubei Provincial Center for Disease Control and Prevention	Bin Fang, Xiang Li, Xiao Yu, Linlin Liu, Bo Yang, Faxian Zhan, Guojun Ye, Xixiang Huo, Junqiang Xu, Bo Yu, Kun Cai, Jing Li, Yongzhong Jiang.
**EPI_ISL_412980**	hCoV-19/Wuhan/HBCDC-HB-04/2020	Asia / China / Hubei / Wuhan	2020-01-18	Union Hospital of Tongji Medical College, Huazhong University of Science and Technology	Hubei Provincial Center for Disease Control and Prevention	Bin Fang, Xiang Li, Xiao Yu, Linlin Liu, Bo Yang, Faxian Zhan, Guojun Ye, Xixiang Huo, Junqiang Xu, Bo Yu, Kun Cai, Jing Li, Yongzhong Jiang.
**EPI_ISL_412981**	hCoV-19/Wuhan/HBCDC-HB-05/2020	Asia / China / Hubei / Wuhan	2020-01-18	CR&WISCO GENERAL HOSPITAL	Hubei Provincial Center for Disease	Bin Fang, Xiang Li, Xiao Yu, Linlin Liu, Bo Yang, Faxian Zhan, Guojun Ye, Xixiang Huo, Junqiang Xu, Bo Yu, Kun Cai, Jing Li, Yongzhong Jiang.

					Control and Prevention	
**EPI_ISL_ 412982**	hCoV-19/Wuhan/HBCDC-HB-06/2020	Asia / China / Hubei / Wuhan	2020-02-07	Wuhan Lung Hospital	Hubei Provincial Center for Disease Control and Prevention	Bin Fang, Xiang Li, Xiao Yu, Linlin Liu, Bo Yang, Faxian Zhan, Guojun Ye, Xixiang Huo, Junqiang Xu, Bo Yu, Kun Cai, Jing Li, Yongzhong Jiang.
**EPI_ISL_ 412983**	hCoV-19/Tianmen/HBCDC-HB-07/2020	Asia / China / Hubei / Tianmen	2020-02-08	Tianmen Center for Disease Control and Prevention	Hubei Provincial Center for Disease Control and Prevention	Bin Fang, Xiang Li, Xiao Yu, Linlin Liu, Bo Yang, Faxian Zhan, Guojun Ye, Xixiang Huo, Junqiang Xu, Bo Yu, Kun Cai, Jing Li, YiFa Zhu, Yangyang Tao,Xierong Li,Yongzhong Jiang.
**EPI_ISL_ 413014**	hCoV-19/Canada/ON-PHL2445/2020	North America / Canada / Ontario	2020-01-25	Public Health Ontario Laboratory	Ontario Agency for Health Protection and Promotion (OAHPP)	Alireza Eshaghi, Samir N Patel, Jonathan B Gubbay, Vanessa G Allen, Christine Frantz, Aimin Li, Sandeep Nagra
**EPI_ISL_ 413015**	hCoV-19/Canada/ON-VIDO-01/2020	North America / Canada / Ontario	2020-01-23	Public Health Ontario Laboratory	National Microbiology Laboratory	Shari Tyson, Anna Majer, Erika Landry, Morag Graham, Grace Seo, Philip Mabon, Natalie Knox, Adrian Zetner, Samira Mubareka, Rob Kozak, Jocelyne Lew, Darryl Falzarano, Gerdts Volker, Jonathan Gubbay, Stephanie Booth, Guillaume Poliquin, Tom Graefenhan, Matthew Gilmour, Nathalie Bastien, Yan Li, Timothy Booth
**EPI_ISL_ 413016**	hCoV-19/Brazil/SPBR-02/2020	South America / Brazil / Sao Paulo / Sao Paulo	2020-02-28	Hospital Israelita Albert Einstein	Instituto Adolfo Lutz, Interdisciplinary Procedures Center, Strategic Laboratory	Jaqueline Goes de Jesus, Claudio Tavares Sacchi, Fabiana Cristina Pereira dos Santos, Ingra Morales Claro, Flávia Cristina da Silva Sales, Claudia Regina Gonçalves, Joshua Quick, Maria do Carmo Sampaio Tavares Timenetsky, Nicholas James Loman, Andrew Rambaut, Ester Cerdeira Sabino, Nuno Rodrigues Faria
**EPI_ISL_ 413017**	hCoV-19/South Korea/KUMC01/2020	Asia / South Korea	2020-02-06	Department of Microbiology, Institute for Viral Diseases, College of Medicine, Korea University	Department of Microbiology, Institute for Viral Diseases, College of Medicine, Korea University	Changmin Kang, Joon-Yong Bae, Jungmin Lee, Heedo Park, Juyoung Cho, Jeonghun Kim, Gee eun Lee, Cui Chunguang, Kyeong-ryeol Shin, Dong Min Kim, Jin Il Kim, Man-Seong Park
**EPI_ISL_ 413018**	hCoV-19/South Korea/KUMC02/2020	Asia / South Korea	2020-02-06	Department of Microbiology, Institute for Viral Diseases, College of Medicine, Korea University	Department of Microbiology, Institute for Viral Diseases, College of Medicine, Korea University	Changmin Kang, Joon-Yong Bae, Jungmin Lee, Heedo Park, Juyoung Cho, Jeonghun Kim, Gee eun Lee, Cui Chunguang, Kyeong-ryeol Shin, Dong Min Kim, Jin Il Kim, Man-Seong Park
**EPI_ISL_ 413019**	hCoV-19/Switzerland/1000477102/2020	Europe / Switzerland / Zurich	2020-02-26	Department of Internal Medicine, Triemli Hospital	Institute of Medical Virology, University of Zurich	Stefan Schmutz, Maryam Zaheri, Verena Kufner, Patrick Redli, Fiona Steiner, Jon Huder, Riccarda Capaul, Andrea Zbinden, Jürg Böni, Michael Huber, Gerhard Eich, Alexandra Trkola
**EPI_ISL_ 413020**	hCoV-19/Switzerland/1000477377/2020	Europe / Switzerland / Zurich	2020-02-27	Department of Internal Medicine, Triemli Hospital	Institute of Medical Virology, University of Zurich	Stefan Schmutz, Maryam Zaheri, Verena Kufner, Patrick Redli, Fiona Steiner, Jon Huder, Riccarda Capaul, Andrea Zbinden, Jürg Böni, Michael Huber, Gerhard Eich, Alexandra Trkola
**EPI_ISL_ 413021**	hCoV-19/Switzerland/1000477757/2020	Europe / Switzerland / Zurich	2020-02-29	Klinik Hirslanden Zurich	Institute of Medical Virology, University of Zurich	Stefan Schmutz, Maryam Zaheri, Verena Kufner, Gabriela Ziltener, Patrick Redli, Fiona Steiner, Jon Huder, Riccarda Capaul, Andrea Zbinden, Jürg Böni, Michael Huber, Christian Ruef, Alexandra Trkola
**EPI_ISL_ 413022**	hCoV-19/Switzerland/1000477796/2020	Europe / Switzerland / Zurich	2020-02-29	Division of Infectious Diseases, University Hospital Zurich	Institute of Medical Virology, University of Zurich	Stefan Schmutz, Maryam Zaheri, Verena Kufner, Gabriela Ziltener, Patrick Redli, Fiona Steiner, Jon Huder, Riccarda Capaul, Andrea Zbinden, Jürg Böni, Michael Huber, Roberto Speck, Alexandra Trkola
**EPI_ISL_ 413023**	hCoV-19/Switzerland/1000477797/2020	Europe / Switzerland / Zurich	2020-02-29	Division of Infectious Diseases,	Institute of Medical Virology, University of Zurich	Stefan Schmutz, Maryam Zaheri, Verena Kufner, Gabriela Ziltener, Patrick Redli, Fiona Steiner, Jon Huder, Riccarda Capaul, Andrea Zbinden, Jürg

				University Hospital Zurich		Böni, Michael Huber, Roberto Speck, Alexandra Trkola
**EPI_ISL_413024**	hCoV-19/Switzerland/1000477806/2020	Europe / Switzerland / Zurich	2020-02-29	Division of Infectious Diseases, University Hospital Zurich	Institute of Medical Virology, University of Zurich	Stefan Schmutz, Maryam Zaheri, Verena Kufner, Gabriela Ziltener, Patrick Redli, Fiona Steiner, Jon Huder, Riccarda Capaul, Andrea Zbinden, Jürg Böni, Michael Huber, Roberto Speck, Alexandra Trkola
**EPI_ISL_413213**	hCoV-19/Australia/NSW06/2020	Oceania / Australia / New South Wales / Sydney	2020-02-29	Centre for Infectious Diseases and Microbiology Laboratory Services	NSW Health Pathology - Institute of Clinical Pathology and Medical Research; Westmead Hospital; University of Sydney	Eden J-S, Carter I, Rahman H, Holmes EC, Rockett R, O'Sullivan MV, Sintchenko V, Chen SC, Maddocks S, Kok J and Dwyer DE for the 2019-nCoV Study Group*
**EPI_ISL_413214**	hCoV-19/Australia/NSW07/2020	Oceania / Australia / New South Wales / Sydney	2020-02-29	Centre for Infectious Diseases and Microbiology Laboratory Services	NSW Health Pathology - Institute of Clinical Pathology and Medical Research; Westmead Hospital; University of Sydney	Eden J-S, Carter I, Rahman H, Holmes EC, Rockett R, O'Sullivan MV, Sintchenko V, Chen SC, Maddocks S, Kok J and Dwyer DE for the 2019-nCoV Study Group*
**EPI_ISL_413221**	hCoV-19/Scotland/CVR01/2020	Europe / United Kingdom / Scotland	2020-03-02	West of Scotland Specialist Virology Centre, NHSGGC	MRC-University of Glasgow Centre for Virus Research	Emma Thomson, Antonia Ho; James Shephard, Shirin Ashraf; Kathy Smollett, Daniel Mair, Stephen Carmichael, Ana da Silva Filipe; Richard Orton, Josh Singer, David L Robertson; Andrew Rambaut; Alasdair MacLean, Rory Gunson.
**EPI_ISL_413455**	hCoV-19/USA/WA4-UW2/2020	North America / USA / Washington	2020-02-28	Washington State Public Health Lab	University of Washington Virology Lab	Pavitra Roychoudhury, Arun Nalla, Hong Xie, Keith Jerome, Alexander Greninger
**EPI_ISL_413456**	hCoV-19/USA/WA-S2/2020	North America / USA / Washington / King County	2020-02-20	Seattle Flu Study	Seattle Flu Study	Chu et al
**EPI_ISL_413457**	hCoV-19/USA/WA6-UW3/2020	North America / USA / Washington	2020-02-29	Washington State Public Health Lab	UW Virology Lab	Pavitra Roychoudhury, Arun Nalla, Hong Xie, Keith Jerome, Alexander Greninger
**EPI_ISL_413458**	hCoV-19/USA/WA7-UW4/2020	North America / USA / Washington	2020-03-01	Washington State Public Health Lab	UW Virology Lab	Pavitra Roychoudhury, Arun Nalla, Hong Xie, Keith Jerome, Alexander Greninger
**EPI_ISL_413485**	hCoV-19/Anhui/SZ005/2020	Asia / China / Anhui / Suzhou	2020-01-24	Department of microbiology laboratory,Anhui Provincial Center for Disease Control and Prevention	Department of microbiology laboratory,Anhui Provincial Center for Disease Control and Prevention	Weiwei Li,Jun He,Yong Sun,Junling Yu,Qingqing Chen,Yuan Yuan,Yonglin Shi,Zhuhui Zhang,Yinglu Ge,Weidong Li,Bin Su,Zhirong Liu
**EPI_ISL_413486**	hCoV-19/USA/WA8-UW5/2020	North America / USA / Washington	2020-03-01	Valley Medical Center	University of Washington Virology Lab	Pavitra Roychoudhury, Arun Nalla, Hong Xie, Keith Jerome, Alexander Greninger
**EPI_ISL_413487**	hCoV-19/USA/WA9-UW6/2020	North America / USA / Washington	2020-03-01	Harborview Medical Center	University of Washington Virology Lab	Pavitra Roychoudhury, Arun Nalla, Hong Xie, Keith Jerome, Alexander Greninger
**EPI_ISL_413488**	hCoV-19/Germany/NRW-01/2020	Europe / Germany / North Rhine Westphalia /	2020-02-28	Center of Medical Microbiology, Virology, and Hospital Hygiene,	Center of Medical Microbiology, Virology, and Hospital Hygiene, University of Duesseldorf	Ortwin Adams, Marcel Andree, Alexander Dilthey, Torsten Feldt, Sandra Hauka, Torsten Houwaart, Björn-Erik Jensen, Detlef Kindgen-Milles, Malte Kohns Vasconcelos, Klaus

		Heinsberg District		University of Duesseldorf		Pfeffer, Tina Senff, Daniel Strelow, Jörg Timm, Andreas Walker, Tobias Wienemann
**EPI_ISL_ 413489**	hCoV-19/Italy/ UniSR1/2020	Europe / Italy / Lombardy / Milan	2020-03-03	Laboratorio di Microbiologia e Virologia, Università Vita-Salute San Raffaele, Milano	Laboratorio di Microbiologia e Virologia, Università Vita-Salute San Raffaele, Milano	R.A Diotti, E. Criscuolo, M. Castelli, V. Caputo, R. Ferrarese, M. Sampaolo, E. Boeri, I. Negri, V. Amato, G. Lo Raso, C. Di Resta, R. Burioni, M. Clementi, N. Mancini & N. Clementi
**EPI_ISL_ 413490**	hCoV-19/New Zealand/01/2020	Oceania / New Zealand / Auckland	2020-02-27	Auckland Hospital	Institute of Environmental Science and Research (ESR)	Matt Storey, Xiaoyun Ren, Gary McAuliffe, Sally Roberts, Matthew Blakiston, Erasmus Smit, Lauren Jelly, Joep de Ligt
**EPI_ISL_ 413519**	hCoV-19/Beijing/231/2020	Asia / China / Beijing	2020-01-28	unknown	Infectious Disease Control Center	Li,J., Li,L., Li,Z., Qiu,S., Song,H., Li,P. and Li,P.
**EPI_ISL_ 413520**	hCoV-19/Beijing/233/2020	Asia / China / Beijing	2020-01-28	unknown	Infectious Disease Control Center	Li,J., Li,L., Li,Z., Qiu,S., Song,H., Li,P. and Li,P.
**EPI_ISL_ 413521**	hCoV-19/Beijing/235/2020	Asia / China / Beijing	2020-01-28	unknown	Infectious Disease Control Center	Li,J., Li,L., Li,Z., Qiu,S., Song,H., Li,P. and Li,P.
**EPI_ISL_ 413522**	hCoV-19/India/1-27/2020	Asia / India / Kerala	2020-01-27	Indian Council of Medical Research - National Institute of Virology	National Influenza Center, Indian Council of Medical Research - National Institute of Virology	Potdar V, Yadav PD, Choudhary ML, Shete-Aich A
**EPI_ISL_ 413523**	hCoV-19/India/1-31/2020	Asia / India / Kerala	2020-01-31	Indian Council of Medical Research-National Institute of Virology	National Influenza Center, Indian Council of Medical Research-National Institute of Virology	Potdar V, Yadav PD, Choudhary ML, Shete-Aich A
**EPI_ISL_ 413550**	hCoV-19/Nigeria/Lagos01/2020	Africa / Nigeria / Lagos	2020-02-27	Centre for Human and Zoonotic Virology (CHAZVY), College of Medicine University of Lagos/Lagos University Teaching Hospital (LUTH), part of the Laboratory Network of the Nigeria Centre for Disease Control (NCDC)	African Centre of Excellence for Genomics of Infectious Diseases (ACEGID), Redeemer's University, Ede, Osun State, Nigeria	Oluniyi P.E., Ajogbasile F.V., Kayode A., Oguzie J., Folarin O.A., Ihekweazu C. Happi C.T.
**EPI_ISL_ 413555**	hCoV-19/Wales/PHW1/2020	Europe / United Kingdom / Wales	2020-02-27	Wales Specialist Virology Centre	Public Health Wales Microbiology Cardiff	Catherine Moore, Cen Sabu, Joanne Watkins, Sally Corden, Tom Connor
**EPI_ISL_ 413556**	hCoV-19/Wales/PHW2/2020	Europe / United Kingdom / Wales	2020-03-04	Wales Specialist Virology Centre	Public Health Wales Microbiology Cardiff	Catherine Moore, Tim Jones, Joanne Watkins, Sally Corden, Tom Connor
**EPI_ISL_ 413557**	hCoV-19/USA/ CA-CDPH-UC1/ 2020	North America / USA / California / Sonoma County	2020-02-28	California Department of Public Health	Chiu Laboratory, University of California, San Francisco	Xianding Deng, Scot Federman, Chao-Yang Pan, Hugo Guevara,Wei Gu, Debra A. Wadford, and Charles Y. Chiu
**EPI_ISL_ 413558**	hCoV-19/USA/ CA-CDPH-UC2/ 2020	North America / USA / California /	2020-02-27	California Department of Public Health	Chiu Laboratory, University of California, San Francisco	Xianding Deng, Scot Federman, Chao-Yang Pan, Hugo Guevara,Wei Gu, Debra A. Wadford, and Charles Y. Chiu

		Solano County				
**EPI_ISL_413559**	hCoV-19/USA/CA-CDPH-UC3/2020	North America / USA / California / Solano County	2020-02-27	California Department of Public Health	Chiu Laboratory, University of California, San Francisco	Xianding Deng, Scot Federman, Chao-Yang Pan, Hugo Guevara,Wei Gu, Debra A. Wadford, and Charles Y. Chiu
**EPI_ISL_413560**	hCoV-19/USA/WA-S3/2020	North America / USA / Washington	2020-02-28	Seattle Flu Study	Seattle Flu Study	Chu et al
**EPI_ISL_413561**	hCoV-19/USA/CA-CDPH-UC4/2020	North America / USA / California / Solano County	2020-02-27	California Department of Public Health	Chiu Laboratory, University of California, San Francisco	Xianding Deng, Scot Federman, Chao-Yang Pan, Hugo Guevara,Wei Gu, Debra A. Wadford, and Charles Y. Chiu
**EPI_ISL_413562**	hCoV-19/USA/WA11-UW7/2020	North America / USA / Washington	2020-03-02	UW Virology Lab	UW Virology Lab	Pavitra Roychoudhury, Hong Xie, Keith Jerome, Alexander Greninger
**EPI_ISL_413563**	hCoV-19/USA/WA12-UW8/2020	North America / USA / Washington	2020-03-03	UW Virology Lab	UW Virology Lab	Pavitra Roychoudhury, Hong Xie, Keith Jerome, Alexander Greninger
**EPI_ISL_413564**	hCoV-19/Netherlands/Andel_1365066/2020	Europe / Netherlands / Andel	2020-03-01	MHC West-Brabant	Erasmus Medical Center	David Nieuwenhuijse, Bas Oude Munnink, Reina Sikkema, Claudia Schapendonk, Irina Chestakova, Anne van der Linden, Mark Pronk, Pascal Lexmond, Corien Swaan, Manon Haverkate, Madelief Mollers, Mart Stein, Sandra Kengne Kamga Mobou, Jeroen van Kampen, Jolanda Voermans, Aura Timen, Corine GeurtsvanKessel, Annemiek van der Eijk, Richard Molenkamp, Marion Koopmans, on behalf of the Dutch national COVID-19 response team.
**EPI_ISL_413565**	hCoV-19/Netherlands/Berlicum_1363564/2020	Europe / Netherlands / Berlicum	2020-02-24	Foundation Pamm	Erasmus Medical Center	David Nieuwenhuijse, Bas Oude Munnink, Reina Sikkema, Claudia Schapendonk, Irina Chestakova, Anne van der Linden, Mark Pronk, Pascal Lexmond, Corien Swaan, Manon Haverkate, Madelief Mollers, Mart Stein, Sandra Kengne Kamga Mobou, Jeroen van Kampen, Jolanda Voermans, Aura Timen, Corine GeurtsvanKessel, Annemiek van der Eijk, Richard Molenkamp, Marion Koopmans, on behalf of the Dutch national COVID-19 response team.
**EPI_ISL_413566**	hCoV-19/Netherlands/Blaricum_1364780/2020	Europe / Netherlands / Blaricum	2020-03-02	MHC Gooi & Vechtstreek	Erasmus Medical Center	David Nieuwenhuijse, Bas Oude Munnink, Reina Sikkema, Claudia Schapendonk, Irina Chestakova, Anne van der Linden, Mark Pronk, Pascal Lexmond, Corien Swaan, Manon Haverkate, Madelief Mollers, Mart Stein, Sandra Kengne Kamga Mobou, Jeroen van Kampen, Jolanda Voermans, Aura Timen, Corine GeurtsvanKessel, Annemiek van der Eijk, Richard Molenkamp, Marion Koopmans, on behalf of the Dutch national COVID-19 response team.
**EPI_ISL_413568**	hCoV-19/Netherlands/Dalen_1363624/2020	Europe / Netherlands / Dalen	2020-03-01	MHC Drente	Erasmus Medical Center	David Nieuwenhuijse, Bas Oude Munnink, Reina Sikkema, Claudia Schapendonk, Irina Chestakova, Anne van der Linden, Mark Pronk, Pascal Lexmond, Corien Swaan, Manon Haverkate, Madelief Mollers, Mart Stein, Sandra Kengne Kamga Mobou, Jeroen van Kampen, Jolanda Voermans, Aura Timen, Corine GeurtsvanKessel, Annemiek van der Eijk, Richard Molenkamp,

						Marion Koopmans, on behalf of the Dutch national COVID-19 response team.
**EPI_ISL_413569**	hCoV-19/Netherlands/Delft_1363424/2020	Europe / Netherlands / Delft	2020-02-28	RIVM	Erasmus Medical Center	David Nieuwenhuijse, Bas Oude Munnink, Reina Sikkema, Claudia Schapendonk, Irina Chestakova, Anne van der Linden, Mark Pronk, Pascal Lexmond, Corien Swaan, Manon Haverkate, Madelief Mollers, Mart Stein, Sandra Kengne Kamga Mobou, Jeroen van Kampen, Jolanda Voermans, Aura Timen, Corine GeurtsvanKessel, Annemiek van der Eijk, Richard Molenkamp, Marion Koopmans, on behalf of the Dutch national COVID-19 response team.
**EPI_ISL_413570**	hCoV-19/Netherlands/Diemen_1363454/2020	Europe / Netherlands / Diemen	2020-02-28	RIVM	Erasmus Medical Center	David Nieuwenhuijse, Bas Oude Munnink, Reina Sikkema, Claudia Schapendonk, Irina Chestakova, Anne van der Linden, Mark Pronk, Pascal Lexmond, Corien Swaan, Manon Haverkate, Madelief Mollers, Mart Stein, Sandra Kengne Kamga Mobou, Jeroen van Kampen, Jolanda Voermans, Aura Timen, Corine GeurtsvanKessel, Annemiek van der Eijk, Richard Molenkamp, Marion Koopmans, on behalf of the Dutch national COVID-19 response team.
**EPI_ISL_413571**	hCoV-19/Netherlands/Eindhoven_1363782/2020	Europe / Netherlands / Eindhoven	2020-03-02	MHC Brabant Zuidoost	Erasmus Medical Center	David Nieuwenhuijse, Bas Oude Munnink, Reina Sikkema, Claudia Schapendonk, Irina Chestakova, Anne van der Linden, Mark Pronk, Pascal Lexmond, Corien Swaan, Manon Haverkate, Madelief Mollers, Mart Stein, Sandra Kengne Kamga Mobou, Jeroen van Kampen, Jolanda Voermans, Aura Timen, Corine GeurtsvanKessel, Annemiek van der Eijk, Richard Molenkamp, Marion Koopmans, on behalf of the Dutch national COVID-19 response team.
**EPI_ISL_413572**	hCoV-19/Netherlands/Haarlem_1363688/2020	Europe / Netherlands / Haarlem	2020-03-01	MHC Kennemerland	Erasmus Medical Center	David Nieuwenhuijse, Bas Oude Munnink, Reina Sikkema, Claudia Schapendonk, Irina Chestakova, Anne van der Linden, Mark Pronk, Pascal Lexmond, Corien Swaan, Manon Haverkate, Madelief Mollers, Mart Stein, Sandra Kengne Kamga Mobou, Jeroen van Kampen, Jolanda Voermans, Aura Timen, Corine GeurtsvanKessel, Annemiek van der Eijk, Richard Molenkamp, Marion Koopmans, on behalf of the Dutch national COVID-19 response team.
**EPI_ISL_413573**	hCoV-19/Netherlands/Hardinxveld_Giessendam_1364806/2020	Europe / Netherlands / Hardinxveld Giessendam	2020-03-02	Dienst Gezondheid & Jeugd Zuid-Holland Zuid	Erasmus Medical Center	David Nieuwenhuijse, Bas Oude Munnink, Reina Sikkema, Claudia Schapendonk, Irina Chestakova, Anne van der Linden, Mark Pronk, Pascal Lexmond, Corien Swaan, Manon Haverkate, Madelief Mollers, Mart Stein, Sandra Kengne Kamga Mobou, Jeroen van Kampen, Jolanda Voermans, Aura Timen, Corine GeurtsvanKessel, Annemiek van der Eijk, Richard Molenkamp, Marion Koopmans, on behalf of the Dutch national COVID-19 response team.
**EPI_ISL_413574**	hCoV-19/Netherlands/Helmond_1363548/2020	Europe / Netherlands / Helmond	2020-02-29	MHC West-Brabant	Erasmus Medical Center	David Nieuwenhuijse, Bas Oude Munnink, Reina Sikkema, Claudia Schapendonk, Irina Chestakova, Anne van der Linden, Mark Pronk, Pascal Lexmond, Corien Swaan, Manon Haverkate, Madelief Mollers, Mart Stein, Sandra Kengne Kamga Mobou, Jeroen van Kampen, Jolanda Voermans, Aura Timen, Corine GeurtsvanKessel, Annemiek van der Eijk, Richard Molenkamp, Marion Koopmans, on behalf of the Dutch national COVID-19 response team.
**EPI_ISL_413575**	hCoV-19/NetherlandsL/Hout	Europe / Netherlands / Houten	2020-02-29	RIVM	Erasmus Medical Center	David Nieuwenhuijse, Bas Oude Munnink, Reina Sikkema, Claudia Schapendonk, Irina Chestakova, Anne van der Linden, Mark Pronk, Pascal

	en_1363498/2020					Lexmond, Corien Swaan, Manon Haverkate, Madelief Mollers, Mart Stein, Sandra Kengne Kamga Mobou, Jeroen van Kampen, Jolanda Voermans, Aura Timen, Corine GeurtsvanKessel, Annemiek van der Eijk, Richard Molenkamp, Marion Koopmans, on behalf of the Dutch national COVID-19 response team.
**EPI_ISL_413576**	hCoV-19/Netherlands/Loon_op_zand_1363512/2020	Europe / Netherlands / Loon op zand	2020-02-29	RIVM	Erasmus Medical Center	David Nieuwenhuijse, Bas Oude Munnink, Reina Sikkema, Claudia Schapendonk, Irina Chestakova, Anne van der Linden, Mark Pronk, Pascal Lexmond, Corien Swaan, Manon Haverkate, Madelief Mollers, Mart Stein, Sandra Kengne Kamga Mobou, Jeroen van Kampen, Jolanda Voermans, Aura Timen, Corine GeurtsvanKessel, Annemiek van der Eijk, Richard Molenkamp, Marion Koopmans, on behalf of the Dutch national COVID-19 response team.
**EPI_ISL_413577**	hCoV-19/Netherlands/Naarden_1364774/2020	Europe / Netherlands / Naarden	2020-03-02	MHC Gooi & Vechtstreek	Erasmus Medical Center	David Nieuwenhuijse, Bas Oude Munnink, Reina Sikkema, Claudia Schapendonk, Irina Chestakova, Anne van der Linden, Mark Pronk, Pascal Lexmond, Corien Swaan, Manon Haverkate, Madelief Mollers, Mart Stein, Sandra Kengne Kamga Mobou, Jeroen van Kampen, Jolanda Voermans, Aura Timen, Corine GeurtsvanKessel, Annemiek van der Eijk, Richard Molenkamp, Marion Koopmans, on behalf of the Dutch national COVID-19 response team.
**EPI_ISL_413578**	hCoV-19/Netherlands/Nieuwendijk_1363582/2020	Europe / Netherlands / Nieuwendijk	2020-03-01	ErasmusMC	Erasmus Medical Center	David Nieuwenhuijse, Bas Oude Munnink, Reina Sikkema, Claudia Schapendonk, Irina Chestakova, Anne van der Linden, Mark Pronk, Pascal Lexmond, Corien Swaan, Manon Haverkate, Madelief Mollers, Mart Stein, Sandra Kengne Kamga Mobou, Jeroen van Kampen, Jolanda Voermans, Aura Timen, Corine GeurtsvanKessel, Annemiek van der Eijk, Richard Molenkamp, Marion Koopmans, on behalf of the Dutch national COVID-19 response team.
**EPI_ISL_413579**	hCoV-19/Netherlands/Nootdorp_1364222/2020	Europe / Netherlands / Nootdorp	2020-03-03	MHC Haaglanden	Erasmus Medical Center	David Nieuwenhuijse, Bas Oude Munnink, Reina Sikkema, Claudia Schapendonk, Irina Chestakova, Anne van der Linden, Mark Pronk, Pascal Lexmond, Corien Swaan, Manon Haverkate, Madelief Mollers, Mart Stein, Sandra Kengne Kamga Mobou, Jeroen van Kampen, Jolanda Voermans, Aura Timen, Corine GeurtsvanKessel, Annemiek van der Eijk, Richard Molenkamp, Marion Koopmans, on behalf of the Dutch national COVID-19 response team.
**EPI_ISL_413580**	hCoV-19/Netherlands/Oisterwijk_1364072/2020	Europe / Netherlands / Oisterwijk	2020-03-02	MHC Hart voor Brabant	Erasmus Medical Center	David Nieuwenhuijse, Bas Oude Munnink, Reina Sikkema, Claudia Schapendonk, Irina Chestakova, Anne van der Linden, Mark Pronk, Pascal Lexmond, Corien Swaan, Manon Haverkate, Madelief Mollers, Mart Stein, Sandra Kengne Kamga Mobou, Jeroen van Kampen, Jolanda Voermans, Aura Timen, Corine GeurtsvanKessel, Annemiek van der Eijk, Richard Molenkamp, Marion Koopmans, on behalf of the Dutch national COVID-19 response team.
**EPI_ISL_413581**	hCoV-19/Netherlands/Oss_1363500/2020	Europe / Netherlands / Oss	2020-02-29	RIVM	Erasmus Medical Center	David Nieuwenhuijse, Bas Oude Munnink, Reina Sikkema, Claudia Schapendonk, Irina Chestakova, Anne van der Linden, Mark Pronk, Pascal Lexmond, Corien Swaan, Manon Haverkate, Madelief Mollers, Mart Stein, Sandra Kengne Kamga Mobou, Jeroen van Kampen, Jolanda Voermans, Aura Timen, Corine GeurtsvanKessel, Annemiek van der Eijk, Richard Molenkamp,

						Marion Koopmans, on behalf of the Dutch national COVID-19 response team.
**EPI_ISL_413582**	hCoV-19/Netherlands/Rotterdam_1363790/2020	Europe / Netherlands / Rotterdam	2020-03-01	ErasmusMC	Erasmus Medical Center	David Nieuwenhuijse, Bas Oude Munnink, Reina Sikkema, Claudia Schapendonk, Irina Chestakova, Anne van der Linden, Mark Pronk, Pascal Lexmond, Corien Swaan, Manon Haverkate, Madelief Mollers, Mart Stein, Sandra Kengne Kamga Mobou, Jeroen van Kampen, Jolanda Voermans, Aura Timen, Corine GeurtsvanKessel, Annemiek van der Eijk, Richard Molenkamp, Marion Koopmans, on behalf of the Dutch national COVID-19 response team.
**EPI_ISL_413583**	hCoV-19/Netherlands/Rotterdam_1364040/2020	Europe / Netherlands / Rotterdam	2020-03-02	MHC Rotterdam-Rijnmond	Erasmus Medical Center	David Nieuwenhuijse, Bas Oude Munnink, Reina Sikkema, Claudia Schapendonk, Irina Chestakova, Anne van der Linden, Mark Pronk, Pascal Lexmond, Corien Swaan, Manon Haverkate, Madelief Mollers, Mart Stein, Sandra Kengne Kamga Mobou, Jeroen van Kampen, Jolanda Voermans, Aura Timen, Corine GeurtsvanKessel, Annemiek van der Eijk, Richard Molenkamp, Marion Koopmans, on behalf of the Dutch national COVID-19 response team.
**EPI_ISL_413584**	hCoV-19/Netherlands/Rotterdam_1364740/2020	Europe / Netherlands / Rotterdam	2020-03-03	unknown	Erasmus Medical Center	David Nieuwenhuijse, Bas Oude Munnink, Reina Sikkema, Claudia Schapendonk, Irina Chestakova, Anne van der Linden, Mark Pronk, Pascal Lexmond, Corien Swaan, Manon Haverkate, Madelief Mollers, Mart Stein, Sandra Kengne Kamga Mobou, Jeroen van Kampen, Jolanda Voermans, Aura Timen, Corine GeurtsvanKessel, Annemiek van der Eijk, Richard Molenkamp, Marion Koopmans, on behalf of the Dutch national COVID-19 response team.
**EPI_ISL_413586**	hCoV-19/Netherlands/Tilburg_1363354/2020	Europe / Netherlands / Tilburg	2020-02-27	Foundation Elisabeth-Tweesteden Ziekenhuis	Erasmus Medical Center	David Nieuwenhuijse, Bas Oude Munnink, Reina Sikkema, Claudia Schapendonk, Irina Chestakova, Anne van der Linden, Mark Pronk, Pascal Lexmond, Corien Swaan, Manon Haverkate, Madelief Mollers, Mart Stein, Sandra Kengne Kamga Mobou, Jeroen van Kampen, Jolanda Voermans, Aura Timen, Corine GeurtsvanKessel, Annemiek van der Eijk, Richard Molenkamp, Marion Koopmans, on behalf of the Dutch national COVID-19 response team.
**EPI_ISL_413587**	hCoV-19/Netherlands/Tilburg_1364286/2020	Europe / Netherlands / Tilburg	2020-03-03	Foundation Elisabeth-Tweesteden Ziekenhuis	Erasmus Medical Center	David Nieuwenhuijse, Bas Oude Munnink, Reina Sikkema, Claudia Schapendonk, Irina Chestakova, Anne van der Linden, Mark Pronk, Pascal Lexmond, Corien Swaan, Manon Haverkate, Madelief Mollers, Mart Stein, Sandra Kengne Kamga Mobou, Jeroen van Kampen, Jolanda Voermans, Aura Timen, Corine GeurtsvanKessel, Annemiek van der Eijk, Richard Molenkamp, Marion Koopmans, on behalf of the Dutch national COVID-19 response team.
**EPI_ISL_413588**	hCoV-19/Netherlands/Utrecht_1363564/2020	Europe / Netherlands / Utrecht	2020-03-01	MHC Utrecht	Erasmus Medical Center	David Nieuwenhuijse, Bas Oude Munnink, Reina Sikkema, Claudia Schapendonk, Irina Chestakova, Anne van der Linden, Mark Pronk, Pascal Lexmond, Corien Swaan, Manon Haverkate, Madelief Mollers, Mart Stein, Sandra Kengne Kamga Mobou, Jeroen van Kampen, Jolanda Voermans, Aura Timen, Corine GeurtsvanKessel, Annemiek van der Eijk, Richard Molenkamp, Marion Koopmans, on behalf of the Dutch national COVID-19 response team.
**EPI_ISL_413589**	hCoV-19/Netherlands/Utrech	Europe / Netherlands / Utrecht	2020-03-01	MHC Utrecht	Erasmus Medical Center	David Nieuwenhuijse, Bas Oude Munnink, Reina Sikkema, Claudia Schapendonk, Irina Chestakova, Anne van der Linden, Mark Pronk, Pascal

	t_1363628/2020					Lexmond, Corien Swaan, Manon Haverkate, Madelief Mollers, Mart Stein, Sandra Kengne Kamga Mobou, Jeroen van Kampen, Jolanda Voermans, Aura Timen, Corine GeurtsvanKessel, Annemiek van der Eijk, Richard Molenkamp, Marion Koopmans, on behalf of the Dutch national COVID-19 response team.
**EPI_ISL_413590**	hCoV-19/Netherlands/Utrecht_1364066/2020	Europe / Netherlands / Utrecht	2020-03-02	MHC Utrecht	Erasmus Medical Center	David Nieuwenhuijse, Bas Oude Munnink, Reina Sikkema, Claudia Schapendonk, Irina Chestakova, Anne van der Linden, Mark Pronk, Pascal Lexmond, Corien Swaan, Manon Haverkate, Madelief Mollers, Mart Stein, Sandra Kengne Kamga Mobou, Jeroen van Kampen, Jolanda Voermans, Aura Timen, Corine GeurtsvanKessel, Annemiek van der Eijk, Richard Molenkamp, Marion Koopmans, on behalf of the Dutch national COVID-19 response team.
**EPI_ISL_413591**	hCoV-19/Netherlands/Zeewolde_1365080/2020	Europe / Netherlands / Zeewolde	2020-03-02	MHC Flevoland	Erasmus Medical Center	David Nieuwenhuijse, Bas Oude Munnink, Reina Sikkema, Claudia Schapendonk, Irina Chestakova, Anne van der Linden, Mark Pronk, Pascal Lexmond, Corien Swaan, Manon Haverkate, Madelief Mollers, Mart Stein, Sandra Kengne Kamga Mobou, Jeroen van Kampen, Jolanda Voermans, Aura Timen, Corine GeurtsvanKessel, Annemiek van der Eijk, Richard Molenkamp, Marion Koopmans, on behalf of the Dutch national COVID-19 response team.
**EPI_ISL_413592**	hCoV-19/Taiwan/NTU03/2020	Asia / Taiwan / Taipei	2020-03-02	Department of Laboratory Medicine, National Taiwan University Hospital	Microbial Genomics Core Lab, National Taiwan University Centers of Genomic and Precision Medicine	Shiou-Hwei Yeh, You-Yu Lin, Ya-Yun Lai, Chiao-Ling Li, Shan-Chwen Chang, Pei-Jer Chen, Sui-Yuan Chang
**EPI_ISL_413593**	hCoV-19/Luxembourg/Lux1/2020	Europe / Luxembourg	2020-02-29	Laboratoire National de Santé	Erasmus Medical Center	David Nieuwenhuijse, Bas Oude Munnink, Reina Sikkema, Claudia Schapendonk, Irina Chestakova, Anne van der Linden, Mark Pronk, Pascal Lexmond, T. Abdelrahman, G. Fournier, J. Mossong, T. Nguyen, Jeroen van Kampen, Jolanda Voermans, Corine GeurtsvanKessel, Annemiek van der Eijk, Richard Molenkamp, Marion Koopmans, on behalf of the Dutch national COVID-19 response team.
**EPI_ISL_413594**	hCoV-19/Australia/NSW08/2020	Oceania / Australia / NSW / Sydney	2020-02-28	Centre for Infectious Diseases and Microbiology Laboratory Services	NSW Health Pathology - Institute of Clinical Pathology and Medical Research; Westmead Hospital; University of Sydney	Rockett R, Eden J-S, Lam C, Gray K, Timms, V, Gall, M, Alicia, A, Carter I, Rahman H, Holmes EC,, O'Sullivan MV, Sintchenko V, Chen SC, Maddocks S, Kok J and Dwyer DE for the 2019-nCoV Study Group*
**EPI_ISL_413596**	hCoV-19/Australia/NSW10/2020	Oceania / Australia / NSW / Sydney	2020-02-28	Centre for Infectious Diseases and Microbiology - Public Health	NSW Health Pathology - Institute of Clinical Pathology and Medical Research; Westmead Hospital; University of Sydney	Rockett R, Eden J-S, Lam C, Gray K, Timms, V, Gall, M, Carter I, Rahman H, Holmes EC, O'Sullivan MV, Sintchenko V, Chen SC, Maddocks S, Kok J and Dwyer DE for the 2019-nCoV Study Group*
**EPI_ISL_413597**	hCoV-19/Australia/NSW11/2020	Oceania / Australia / NSW / Sydney	2020-03-02	Centre for Infectious Diseases and Microbiology-Public Health	NSW Health Pathology - Institute of Clinical Pathology and Medical Research; Westmead Hospital; University of Sydney	Lam C, Eden J-S, Rockett R, Gray K, Timms, V, Gall, M, Carter I, Rahman H, Holmes EC, O'Sullivan MV, Sintchenko V, Chen SC, Maddocks S, Kok J and Dwyer DE for the 2019-nCoV Study Group*
**EPI_ISL_413598**	hCoV-19/Australia/NSW12/2020	Oceania / Australia / NSW / Sydney	2020-03-04	Centre for Infectious Diseases and Microbiology - Public Health	NSW Health Pathology - Institute of Clinical Pathology and Medical Research; Westmead	Gray K, Eden J-S, Lam C, Rockett R, Timms, V, Gall, M, Carter I, Rahman H, Holmes EC, O'Sullivan MV, Sintchenko V, Chen SC, Maddocks S, Kok J and Dwyer DE for the 2019-nCoV Study Group*

					Hospital; University of Sydney	
**EPI_ISL_413599**	hCoV-19/Australia/NSW13/2020	Oceania / Australia / NSW / Sydney	2020-03-04	Centre for Infectious Diseases and Microbiology - Public Health	NSW Health Pathology - Institute of Clinical Pathology and Medical Research; Westmead Hospital; University of Sydney	Timms, V, Eden J-S, Lam C, Gray K, Rockett R, Gall, M, Carter I, Rahman H, Holmes EC, O'Sullivan MV, Sintchenko V, Chen SC, Maddocks S, Kok J and Dwyer DE for the 2019-nCoV Study Group*
**EPI_ISL_413600**	hCoV-19/Australia/NSW14/2020	Oceania / Australia / NSW / Sydney	2020-03-03	Centre for Infectious Diseases and Microbiology - Public Health	NSW Health Pathology - Institute of Clinical Pathology and Medical Research; Westmead Hospital; University of Sydney	Gall, M, Eden J-S, Lam C, Gray K, Timms, V, Rockett R, Carter I, Rahman H, Holmes EC, O'Sullivan MV, Sintchenko V, Chen SC, Maddocks S, Kok J and Dwyer DE for the 2019-nCoV Study Group*
**EPI_ISL_413601**	hCoV-19/USA/WA13-UW9/2020	North America / USA / Washington	2020-03-02	UW Virology Lab	UW Virology Lab	Pavitra Roychoudhury, Hong Xie, Keith Jerome, Alexander Greninger
**EPI_ISL_413602**	hCoV-19/Finland/FIN03032020A/2020	Europe / Finland / Helsinki	2020-03-03	Department of Virology and Immunology, University of Helsinki and Helsinki University Hospital, Huslab Finland	Department of Virology, Faculty of Medicine, University of Helsinki, Helsinki, Finland	Teemu Smura, Hannimari Kallio-Kokko, Olli Vapalahti
**EPI_ISL_413603**	hCoV-19/Finland/FIN03032020B/2020	Europe / Finland / Helsinki	2020-03-03	Department of Virology and Immunology, University of Helsinki and Helsinki University Hospital, Huslab Finland	Department of Virology, Faculty of Medicine, University of Helsinki, Helsinki, Finland	Teemu Smura, Hannimari Kallio-Kokko, Olli Vapalahti
**EPI_ISL_413604**	hCoV-19/Finland/FIN03032020C/2020	Europe / Finland / Helsinki	2020-03-03	Department of Virology and Immunology, University of Helsinki and Helsinki University Hospital, Huslab Finland	Department of Virology, Faculty of Medicine, University of Helsinki, Helsinki, Finland	Teemu Smura, Hannimari Kallio-Kokko, Olli Vapalahti
**EPI_ISL_413605**	hCoV-19/Finland/FIN01032020/2020	Europe / Finland / Helsinki	2020-03-01	Department of Virology and Immunology, University of Helsinki and Helsinki University Hospital, Huslab Finland	Department of Virology, Faculty of Medicine, University of Helsinki, Helsinki, Finland	Teemu Smura, Hannimari Kallio-Kokko, Olli Vapalahti
**EPI_ISL_413647**	hCoV-19/Portugal/CV62/2020	Europe / Portugal	2020-03-01	Centro Hospital do Porto, E.P.E. - H. Geral de Santo Antonio	Instituto Nacional de Saude (INSA)	Raquel Guiomar, Inês Costa, Pedro Pechirra, Joana Mendonça, Luís Vieira, Helena Ramos, Joana Isidro, Vítor Borges, João Paulo Gomes
**EPI_ISL_413648**	hCoV-19/Portugal/CV63/2020	Europe / Portugal	2020-03-01	Centro Hospitalar e Universitário de Sao Joao, Porto	Instituto Nacional de Saude (INSA)	Raquel Guiomar, Inês Costa, Pedro Pechirra, Joana Mendonça, Luís Vieira, João Tiago Guimarães, Joana Isidro, Vítor Borges, João Paulo Gomes
**EPI_ISL_413649**	hCoV-19/USA/WA14-UW10/2020	North America / USA / Washington	2020-03-05	UW Virology Lab	UW Virology Lab	Pavitra Roychoudhury, Hong Xie, Keith Jerome, Alexander Greninger

**EPI_ISL_ 413650**	hCoV-19/USA/ WA15-UW11/2 020	North America / USA / Washington	2020-03-05	UW Virology Lab	UW Virology Lab	Pavitra Roychoudhury, Hong Xie, Keith Jerome, Alexander Greninger
**EPI_ISL_ 413651**	hCoV-19/USA/ WA16-UW12/2 020	North America / USA / Washington	2020-03-05	UW Virology Lab	UW Virology Lab	Pavitra Roychoudhury, Hong Xie, Keith Jerome, Alexander Greninger
**EPI_ISL_ 413652**	hCoV-19/USA/ WA17-UW13/2 020	North America / USA / Washington	2020-03-05	UW Virology Lab	UW Virology Lab	Pavitra Roychoudhury, Hong Xie, Keith Jerome, Alexander Greninger
**EPI_ISL_ 413653**	hCoV-19/USA/ WA18-UW14/2 020	North America / USA / Washington	2020-03-05	UW Virology Lab	UW Virology Lab	Pavitra Roychoudhury, Hong Xie, Keith Jerome, Alexander Greninger
**EPI_ISL_ 413691**	hCoV-19/China /WF0001/2020	Asia / China	2020-01	Weifang Center for Disease Control and Prevention	Weifang Center for Disease Control and Prevention & BGI-Shenzhen	Qing Nie, Xingguang Li, Erik M Volz, Han Fu, Haowei Wang, Xiaoyue Xi, Wei Chen, Dehui Liu, Yingying Chen, Mengmeng Tian, Wei Tan, Junjie Zai, Wanying Sun, Jiandong Li, Junhua Li
**EPI_ISL_ 413692**	hCoV-19/China /WF0002/2020	Asia / China	2020-01	Weifang Center for Disease Control and Prevention	Weifang Center for Disease Control and Prevention & BGI-Shenzhen	Qing Nie, Xingguang Li, Erik M Volz, Han Fu, Haowei Wang, Xiaoyue Xi, Wei Chen, Dehui Liu, Yingying Chen, Mengmeng Tian, Wei Tan, Junjie Zai, Wanying Sun, Jiandong Li, Junhua Li
**EPI_ISL_ 413693**	hCoV-19/China /WF0003/2020	Asia / China	2020-01	Weifang Center for Disease Control and Prevention	Weifang Center for Disease Control and Prevention & BGI-Shenzhen	Qing Nie, Xingguang Li, Erik M Volz, Han Fu, Haowei Wang, Xiaoyue Xi, Wei Chen, Dehui Liu, Yingying Chen, Mengmeng Tian, Wei Tan, Junjie Zai, Wanying Sun, Jiandong Li, Junhua Li
**EPI_ISL_ 413694**	hCoV-19/China /WF0004/2020	Asia / China	2020-01	Weifang Center for Disease Control and Prevention	Weifang Center for Disease Control and Prevention & BGI-Shenzhen	Qing Nie, Xingguang Li, Erik M Volz, Han Fu, Haowei Wang, Xiaoyue Xi, Wei Chen, Dehui Liu, Yingying Chen, Mengmeng Tian, Wei Tan, Junjie Zai, Wanying Sun, Jiandong Li, Junhua Li
**EPI_ISL_ 413697**	hCoV-19/China /WF0012/2020	Asia / China	2020-02	Weifang Center for Disease Control and Prevention	Weifang Center for Disease Control and Prevention & BGI-Shenzhen	Qing Nie, Xingguang Li, Erik M Volz, Han Fu, Haowei Wang, Xiaoyue Xi, Wei Chen, Dehui Liu, Yingying Chen, Mengmeng Tian, Wei Tan, Junjie Zai, Wanying Sun, Jiandong Li, Junhua Li
**EPI_ISL_ 413711**	hCoV-19/China /WF0014/2020	Asia / China	2020-02	Weifang Center for Disease Control and Prevention	Weifang Center for Disease Control and Prevention & BGI-Shenzhen	Qing Nie, Xingguang Li, Erik M Volz, Han Fu, Haowei Wang, Xiaoyue Xi, Wei Chen, Dehui Liu, Yingying Chen, Mengmeng Tian, Wei Tan, Junjie Zai, Wanying Sun, Jiandong Li, Junhua Li
**EPI_ISL_ 413729**	hCoV-19/China /WF0015/2020	Asia / China	2020-02	Weifang Center for Disease Control and Prevention	Weifang Center for Disease Control and Prevention & BGI-Shenzhen	Qing Nie, Xingguang Li, Erik M Volz, Han Fu, Haowei Wang, Xiaoyue Xi, Wei Chen, Dehui Liu, Yingying Chen, Mengmeng Tian, Wei Tan, Junjie Zai, Wanying Sun, Jiandong Li, Junhua Li
**EPI_ISL_ 413746**	hCoV-19/China /WF0016/2020	Asia / China	2020-02	Weifang Center for Disease Control and Prevention	Weifang Center for Disease Control and Prevention & BGI-Shenzhen	Qing Nie, Xingguang Li, Erik M Volz, Han Fu, Haowei Wang, Xiaoyue Xi, Wei Chen, Dehui Liu, Yingying Chen, Mengmeng Tian, Wei Tan, Junjie Zai, Wanying Sun, Jiandong Li, Junhua Li
**EPI_ISL_ 413748**	hCoV-19/China /WF0018/2020	Asia / China	2020-02	Weifang Center for Disease Control and Prevention	Weifang Center for Disease Control and Prevention & BGI-Shenzhen	Qing Nie, Xingguang Li, Erik M Volz, Han Fu, Haowei Wang, Xiaoyue Xi, Wei Chen, Dehui Liu, Yingying Chen, Mengmeng Tian, Wei Tan, Junjie Zai, Wanying Sun, Jiandong Li, Junhua Li
**EPI_ISL_ 413749**	hCoV-19/China /WF0019/2020	Asia / China	2020-02	Weifang Center for Disease Control and Prevention	Weifang Center for Disease Control and Prevention & BGI-Shenzhen	Qing Nie, Xingguang Li, Erik M Volz, Han Fu, Haowei Wang, Xiaoyue Xi, Wei Chen, Dehui Liu, Yingying Chen, Mengmeng Tian, Wei Tan, Junjie Zai, Wanying Sun, Jiandong Li, Junhua Li
**EPI_ISL_ 413750**	hCoV-19/China /WF0020/2020	Asia / China	2020-02	Weifang Center for Disease Control and Prevention	Weifang Center for Disease Control and Prevention & BGI-Shenzhen	Qing Nie, Xingguang Li, Erik M Volz, Han Fu, Haowei Wang, Xiaoyue Xi, Wei Chen, Dehui Liu, Yingying Chen, Mengmeng Tian, Wei Tan, Junjie Zai, Wanying Sun, Jiandong Li, Junhua Li

**EPI_ISL_413751**	hCoV-19/China/WF0021/2020	Asia / China	2020-02	Weifang Center for Disease Control and Prevention	Weifang Center for Disease Control and Prevention & BGI-Shenzhen	Qing Nie, Xingguang Li, Erik M Volz, Han Fu, Haowei Wang, Xiaoyue Xi, Wei Chen, Dehui Liu, Yingying Chen, Mengmeng Tian, Wei Tan, Junjie Zai, Wanying Sun, Jiandong Li, Junhua Li
**EPI_ISL_413752**	hCoV-19/China/WF0023/2020	Asia / China	2020-02	Weifang Center for Disease Control and Prevention	Weifang Center for Disease Control and Prevention & BGI-Shenzhen	Qing Nie, Xingguang Li, Erik M Volz, Han Fu, Haowei Wang, Xiaoyue Xi, Wei Chen, Dehui Liu, Yingying Chen, Mengmeng Tian, Wei Tan, Junjie Zai, Wanying Sun, Jiandong Li, Junhua Li
**EPI_ISL_413753**	hCoV-19/China/WF0024/2020	Asia / China	2020-02	Weifang Center for Disease Control and Prevention	Weifang Center for Disease Control and Prevention & BGI-Shenzhen	Qing Nie, Xingguang Li, Erik M Volz, Han Fu, Haowei Wang, Xiaoyue Xi, Wei Chen, Dehui Liu, Yingying Chen, Mengmeng Tian, Wei Tan, Junjie Zai, Wanying Sun, Jiandong Li, Junhua Li
**EPI_ISL_413761**	hCoV-19/China/WF0026/2020	Asia / China	2020-02	Weifang Center for Disease Control and Prevention	Weifang Center for Disease Control and Prevention & BGI-Shenzhen	Qing Nie, Xingguang Li, Erik M Volz, Han Fu, Haowei Wang, Xiaoyue Xi, Wei Chen, Dehui Liu, Yingying Chen, Mengmeng Tian, Wei Tan, Junjie Zai, Wanying Sun, Jiandong Li, Junhua Li
**EPI_ISL_413791**	hCoV-19/China/WF0028/2020	Asia / China	2020-02	Weifang Center for Disease Control and Prevention	Weifang Center for Disease Control and Prevention & BGI-Shenzhen	Qing Nie, Xingguang Li, Erik M Volz, Han Fu, Haowei Wang, Xiaoyue Xi, Wei Chen, Dehui Liu, Yingying Chen, Mengmeng Tian, Wei Tan, Junjie Zai, Wanying Sun, Jiandong Li, Junhua Li
**EPI_ISL_413809**	hCoV-19/China/WF0029/2020	Asia / China	2020-02	Weifang Center for Disease Control and Prevention	Weifang Center for Disease Control and Prevention & BGI-Shenzhen	Qing Nie, Xingguang Li, Erik M Volz, Han Fu, Haowei Wang, Xiaoyue Xi, Wei Chen, Dehui Liu, Yingying Chen, Mengmeng Tian, Wei Tan, Junjie Zai, Wanying Sun, Jiandong Li, Junhua Li
**EPI_ISL_413850**	hCoV-19/Guangdong/GDFS2020054-P0005/2020	Asia / China / Guangdong	2020-02-10	Guangdong Provincial Institution of Public Health, Guangdong Provincial Center for Disease Control and Prevention	Guangdong Provincial Institution of Public Health	Jing Lu, Louis du Plessis, Liu Zhe, Jiufeng Sun, Sarah François, Huifang Lin, Moritz Kraemer, Jingju Peng, Qianlin Xiong, Runyu Yuan, Lilian Zeng, Pingping Zhou, Chuming Liang, Tao Liu, Wei Li, Juan Su, Huanying Zheng, Kang Min, Song Tie, Bo Peng, Shisong Fang, Wenzhe Su, Kuibiao Li, Ruilin Sun, Ru bai, Xi Tang, Minfeng Liang, Nuno Faria, Josh Quick, Andrew Rambaut, Verity Hill, Wenjun Ma, Nick Loman, Oliver Pybus, Changwen Ke
**EPI_ISL_413851**	hCoV-19/Guangdong/2020XN4373-P0039/2020	Asia / China / Guangdong	2020-01-30	Guangdong Provincial Institution of Public Health, Guangdong Provincial Center for Disease Control and Prevention	Guangdong Provincial Institution of Public Health	Jing Lu, Louis du Plessis, Liu Zhe, Jiufeng Sun, Sarah François, Huifang Lin, Moritz Kraemer, Jingju Peng, Qianlin Xiong, Runyu Yuan, Lilian Zeng, Pingping Zhou, Chuming Liang, Tao Liu, Wei Li, Juan Su, Huanying Zheng, Kang Min, Song Tie, Bo Peng, Shisong Fang, Wenzhe Su, Kuibiao Li, Ruilin Sun, Ru bai, Xi Tang, Minfeng Liang, Nuno Faria, Josh Quick, Andrew Rambaut, Verity Hill, Wenjun Ma, Nick Loman, Oliver Pybus, Changwen Ke
**EPI_ISL_413852**	hCoV-19/Guangdong/2020XN4433-P0040/2020	Asia / China / Guangdong	2020-01-30	Guangdong Provincial Institution of Public Health, Guangdong Provincial Center for Disease Control and Prevention	Guangdong Provincial Institution of Public Health	Jing Lu, Louis du Plessis, Liu Zhe, Jiufeng Sun, Sarah François, Huifang Lin, Moritz Kraemer, Jingju Peng, Qianlin Xiong, Runyu Yuan, Lilian Zeng, Pingping Zhou, Chuming Liang, Tao Liu, Wei Li, Juan Su, Huanying Zheng, Kang Min, Song Tie, Bo Peng, Shisong Fang, Wenzhe Su, Kuibiao Li, Ruilin Sun, Ru bai, Xi Tang, Minfeng Liang, Nuno Faria, Josh Quick, Andrew Rambaut, Verity Hill, Wenjun Ma, Nick Loman, Oliver Pybus, Changwen Ke
**EPI_ISL_413853**	hCoV-19/Guangdong/2020XN4243-P0035/2020	Asia / China / Guangdong	2020-01-30	Guangdong Provincial Institution of Public Health, Guangdong Provincial Center for Disease Control and Prevention	Guangdong Provincial Institution of Public Health	Jing Lu, Louis du Plessis, Liu Zhe, Jiufeng Sun, Sarah François, Huifang Lin, Moritz Kraemer, Jingju Peng, Qianlin Xiong, Runyu Yuan, Lilian Zeng, Pingping Zhou, Chuming Liang, Tao Liu, Wei Li, Juan Su, Huanying Zheng, Kang Min, Song Tie, Bo Peng, Shisong Fang, Wenzhe Su, Kuibiao Li, Ruilin Sun, Ru bai, Xi Tang, Minfeng Liang, Nuno Faria, Josh Quick, Andrew Rambaut, Verity Hill, Wenjun Ma, Nick Loman, Oliver Pybus, Changwen Ke

**EPI_ISL_413854**	hCoV-19/Guangdong/2020XN4475-P0042/2020	Asia / China / Guangdong	2020-01-30	Guangdong Provincial Institution of Public Health, Guangdong Provincial Center for Disease Control and Prevention	Guangdong Provincial Institution of Public Health	Jing Lu, Louis du Plessis, Liu Zhe, Jiufeng Sun, Sarah François, Huifang Lin, Moritz Kraemer, Jingju Peng, Qianlin Xiong, Runyu Yuan, Lilian Zeng, Pingping Zhou, Chuming Liang, Tao Liu, Wei Li, Juan Su, Huanying Zheng, Kang Min, Song Tie, Bo Peng, Shisong Fang, Wenzhe Su, Kuibiao Li, Ruilin Sun, Ru bai, Xi Tang, Minfeng Liang, Nuno Faria, Josh Quick, Andrew Rambaut, Verity Hill, Wenjun Ma, Nick Loman, Oliver Pybus, Changwen Ke
**EPI_ISL_413855**	hCoV-19/Guangdong/GD2020234-P0023/2020	Asia / China / Guangdong	2020-02-07	Guangdong Provincial Institution of Public Health, Guangdong Provincial Center for Disease Control and Prevention	Guangdong Provincial Institution of Public Health	Jing Lu, Louis du Plessis, Liu Zhe, Jiufeng Sun, Sarah François, Huifang Lin, Moritz Kraemer, Jingju Peng, Qianlin Xiong, Runyu Yuan, Lilian Zeng, Pingping Zhou, Chuming Liang, Tao Liu, Wei Li, Juan Su, Huanying Zheng, Kang Min, Song Tie, Bo Peng, Shisong Fang, Wenzhe Su, Kuibiao Li, Ruilin Sun, Ru bai, Xi Tang, Minfeng Liang, Nuno Faria, Josh Quick, Andrew Rambaut, Verity Hill, Wenjun Ma, Nick Loman, Oliver Pybus, Changwen Ke
**EPI_ISL_413856**	hCoV-19/Guangdong/GD2020233-P0027/2020	Asia / China / Guangdong	2020-02-07	Guangdong Provincial Institution of Public Health, Guangdong Provincial Center for Disease Control and Prevention	Guangdong Provincial Institution of Public Health	Jing Lu, Louis du Plessis, Liu Zhe, Jiufeng Sun, Sarah François, Huifang Lin, Moritz Kraemer, Jingju Peng, Qianlin Xiong, Runyu Yuan, Lilian Zeng, Pingping Zhou, Chuming Liang, Tao Liu, Wei Li, Juan Su, Huanying Zheng, Kang Min, Song Tie, Bo Peng, Shisong Fang, Wenzhe Su, Kuibiao Li, Ruilin Sun, Ru bai, Xi Tang, Minfeng Liang, Nuno Faria, Josh Quick, Andrew Rambaut, Verity Hill, Wenjun Ma, Nick Loman, Oliver Pybus, Changwen Ke
**EPI_ISL_413857**	hCoV-19/Guangdong/2020XN4448-P0002/2020	Asia / China / Guangdong	2020-01-31	Guangdong Provincial Institution of Public Health, Guangdong Provincial Center for Disease Control and Prevention	Guangdong Provincial Institution of Public Health	Jing Lu, Louis du Plessis, Liu Zhe, Jiufeng Sun, Sarah François, Huifang Lin, Moritz Kraemer, Jingju Peng, Qianlin Xiong, Runyu Yuan, Lilian Zeng, Pingping Zhou, Chuming Liang, Tao Liu, Wei Li, Juan Su, Huanying Zheng, Kang Min, Song Tie, Bo Peng, Shisong Fang, Wenzhe Su, Kuibiao Li, Ruilin Sun, Ru bai, Xi Tang, Minfeng Liang, Nuno Faria, Josh Quick, Andrew Rambaut, Verity Hill, Wenjun Ma, Nick Loman, Oliver Pybus, Changwen Ke
**EPI_ISL_413858**	hCoV-19/Guangdong/2020XN4459-P0041/2020	Asia / China / Guangdong	2020-01-30	Guangdong Provincial Institution of Public Health, Guangdong Provincial Center for Disease Control and Prevention	Guangdong Provincial Institution of Public Health	Jing Lu, Louis du Plessis, Liu Zhe, Jiufeng Sun, Sarah François, Huifang Lin, Moritz Kraemer, Jingju Peng, Qianlin Xiong, Runyu Yuan, Lilian Zeng, Pingping Zhou, Chuming Liang, Tao Liu, Wei Li, Juan Su, Huanying Zheng, Kang Min, Song Tie, Bo Peng, Shisong Fang, Wenzhe Su, Kuibiao Li, Ruilin Sun, Ru bai, Xi Tang, Minfeng Liang, Nuno Faria, Josh Quick, Andrew Rambaut, Verity Hill, Wenjun Ma, Nick Loman, Oliver Pybus, Changwen Ke
**EPI_ISL_413859**	hCoV-19/Guangdong/2020XN4276-P0037/2020	Asia / China / Guangdong	2020-01-31	Guangdong Provincial Institution of Public Health, Guangdong Provincial Center for Disease Control and Prevention	Guangdong Provincial Institution of Public Health	Jing Lu, Louis du Plessis, Liu Zhe, Jiufeng Sun, Sarah François, Huifang Lin, Moritz Kraemer, Jingju Peng, Qianlin Xiong, Runyu Yuan, Lilian Zeng, Pingping Zhou, Chuming Liang, Tao Liu, Wei Li, Juan Su, Huanying Zheng, Kang Min, Song Tie, Bo Peng, Shisong Fang, Wenzhe Su, Kuibiao Li, Ruilin Sun, Ru bai, Xi Tang, Minfeng Liang, Nuno Faria, Josh Quick, Andrew Rambaut, Verity Hill, Wenjun Ma, Nick Loman, Oliver Pybus, Changwen Ke
**EPI_ISL_413860**	hCoV-19/Guangdong/2020XN4273-P0036/2020	Asia / China / Guangdong	2020-01-30	Guangdong Provincial Institution of Public Health, Guangdong Provincial Center	Guangdong Provincial Institution of Public Health	Jing Lu, Louis du Plessis, Liu Zhe, Jiufeng Sun, Sarah François, Huifang Lin, Moritz Kraemer, Jingju Peng, Qianlin Xiong, Runyu Yuan, Lilian Zeng, Pingping Zhou, Chuming Liang, Tao Liu, Wei Li, Juan Su, Huanying Zheng, Kang Min, Song Tie, Bo Peng, Shisong Fang, Wenzhe Su, Kuibiao Li,

				for Disease Control and Prevention		Ruilin Sun, Ru bai, Xi Tang, Minfeng Liang, Nuno Faria, Josh Quick, Andrew Rambaut, Verity Hill, Wenjun Ma, Nick Loman, Oliver Pybus, Changwen Ke
**EPI_ISL_413861**	hCoV-19/Guangdong/GD2020080-P0010/2020	Asia / China / Guangdong	2020-02-01	Guangdong Provincial Institution of Public Health, Guangdong Provincial Center for Disease Control and Prevention	Guangdong Provincial Institution of Public Health	Jing Lu, Louis du Plessis, Liu Zhe, Jiufeng Sun, Sarah François, Huifang Lin, Moritz Kraemer, Jingju Peng, Qianlin Xiong, Runyu Yuan, Lilian Zeng, Pingping Zhou, Chuming Liang, Tao Liu, Wei Li, Juan Su, Huanying Zheng, Kang Min, Song Tie, Bo Peng, Shisong Fang, Wenzhe Su, Kuibiao Li, Ruilin Sun, Ru bai, Xi Tang, Minfeng Liang, Nuno Faria, Josh Quick, Andrew Rambaut, Verity Hill, Wenjun Ma, Nick Loman, Oliver Pybus, Changwen Ke
**EPI_ISL_413862**	hCoV-19/Guangdong/GD2020086-P0021/2020	Asia / China / Guangdong	2020-02-01	Guangdong Provincial Institution of Public Health, Guangdong Provincial Center for Disease Control and Prevention	Guangdong Provincial Institution of Public Health	Jing Lu, Louis du Plessis, Liu Zhe, Jiufeng Sun, Sarah François, Huifang Lin, Moritz Kraemer, Jingju Peng, Qianlin Xiong, Runyu Yuan, Lilian Zeng, Pingping Zhou, Chuming Liang, Tao Liu, Wei Li, Juan Su, Huanying Zheng, Kang Min, Song Tie, Bo Peng, Shisong Fang, Wenzhe Su, Kuibiao Li, Ruilin Sun, Ru bai, Xi Tang, Minfeng Liang, Nuno Faria, Josh Quick, Andrew Rambaut, Verity Hill, Wenjun Ma, Nick Loman, Oliver Pybus, Changwen Ke
**EPI_ISL_413863**	hCoV-19/Guangdong/GD2020087-P0008/2020	Asia / China / Guangdong	2020-02-01	Guangdong Provincial Institution of Public Health, Guangdong Provincial Center for Disease Control and Prevention	Guangdong Provincial Institution of Public Health	Jing Lu, Louis du Plessis, Liu Zhe, Jiufeng Sun, Sarah François, Huifang Lin, Moritz Kraemer, Jingju Peng, Qianlin Xiong, Runyu Yuan, Lilian Zeng, Pingping Zhou, Chuming Liang, Tao Liu, Wei Li, Juan Su, Huanying Zheng, Kang Min, Song Tie, Bo Peng, Shisong Fang, Wenzhe Su, Kuibiao Li, Ruilin Sun, Ru bai, Xi Tang, Minfeng Liang, Nuno Faria, Josh Quick, Andrew Rambaut, Verity Hill, Wenjun Ma, Nick Loman, Oliver Pybus, Changwen Ke
**EPI_ISL_413864**	hCoV-19/Guangdong/GD2020246-P0028/2020	Asia / China / Guangdong	2020-02-09	Guangdong Provincial Institution of Public Health, Guangdong Provincial Center for Disease Control and Prevention	Guangdong Provincial Institution of Public Health	Jing Lu, Louis du Plessis, Liu Zhe, Jiufeng Sun, Sarah François, Huifang Lin, Moritz Kraemer, Jingju Peng, Qianlin Xiong, Runyu Yuan, Lilian Zeng, Pingping Zhou, Chuming Liang, Tao Liu, Wei Li, Juan Su, Huanying Zheng, Kang Min, Song Tie, Bo Peng, Shisong Fang, Wenzhe Su, Kuibiao Li, Ruilin Sun, Ru bai, Xi Tang, Minfeng Liang, Nuno Faria, Josh Quick, Andrew Rambaut, Verity Hill, Wenjun Ma, Nick Loman, Oliver Pybus, Changwen Ke
**EPI_ISL_413866**	hCoV-19/Guangdong/GDSZ202008-P0020/2020	Asia / China / Guangdong	2020-02-05	Guangdong Provincial Institution of Public Health, Guangdong Provincial Center for Disease Control and Prevention	Guangdong Provincial Institution of Public Health	Jing Lu, Louis du Plessis, Liu Zhe, Jiufeng Sun, Sarah François, Huifang Lin, Moritz Kraemer, Jingju Peng, Qianlin Xiong, Runyu Yuan, Lilian Zeng, Pingping Zhou, Chuming Liang, Tao Liu, Wei Li, Juan Su, Huanying Zheng, Kang Min, Song Tie, Bo Peng, Shisong Fang, Wenzhe Su, Kuibiao Li, Ruilin Sun, Ru bai, Xi Tang, Minfeng Liang, Nuno Faria, Josh Quick, Andrew Rambaut, Verity Hill, Wenjun Ma, Nick Loman, Oliver Pybus, Changwen Ke
**EPI_ISL_413867**	hCoV-19/Guangdong/GDSZ202015-P0019/2020	Asia / China / Guangdong	2020-02-05	Guangdong Provincial Institution of Public Health, Guangdong Provincial Center for Disease Control and Prevention	Guangdong Provincial Institution of Public Health	Jing Lu, Louis du Plessis, Liu Zhe, Jiufeng Sun, Sarah François, Huifang Lin, Moritz Kraemer, Jingju Peng, Qianlin Xiong, Runyu Yuan, Lilian Zeng, Pingping Zhou, Chuming Liang, Tao Liu, Wei Li, Juan Su, Huanying Zheng, Kang Min, Song Tie, Bo Peng, Shisong Fang, Wenzhe Su, Kuibiao Li, Ruilin Sun, Ru bai, Xi Tang, Minfeng Liang, Nuno Faria, Josh Quick, Andrew Rambaut, Verity Hill, Wenjun Ma, Nick Loman, Oliver Pybus, Changwen Ke

**EPI_ISL_413868**	hCoV-19/Guangdong/DG-S9-P0045/2020	Asia / China / Guangdong	2020-02-28	Guangdong Provincial Institution of Public Health, Guangdong Provincial Center for Disease Control and Prevention	Guangdong Provincial Institution of Public Health	Jing Lu, Louis du Plessis, Liu Zhe, Jiufeng Sun, Sarah François, Huifang Lin, Moritz Kraemer, Jingju Peng, Qianlin Xiong, Runyu Yuan, Lilian Zeng, Pingping Zhou, Chuming Liang, Tao Liu, Wei Li, Juan Su, Huanying Zheng, Kang Min, Song Tie, Bo Peng, Shisong Fang, Wenzhe Su, Kuibiao Li, Ruilin Sun, Ru bai, Xi Tang, Minfeng Liang, Nuno Faria, Josh Quick, Andrew Rambaut, Verity Hill, Wenjun Ma, Nick Loman, Oliver Pybus, Changwen Ke
**EPI_ISL_413869**	hCoV-19/Guangdong/FS-S42-P0046/2020	Asia / China / Guangdong	2020-02-28	Guangdong Provincial Institution of Public Health, Guangdong Provincial Center for Disease Control and Prevention	Guangdong Provincial Institution of Public Health	Jing Lu, Louis du Plessis, Liu Zhe, Jiufeng Sun, Sarah François, Huifang Lin, Moritz Kraemer, Jingju Peng, Qianlin Xiong, Runyu Yuan, Lilian Zeng, Pingping Zhou, Chuming Liang, Tao Liu, Wei Li, Juan Su, Huanying Zheng, Kang Min, Song Tie, Bo Peng, Shisong Fang, Wenzhe Su, Kuibiao Li, Ruilin Sun, Ru bai, Xi Tang, Minfeng Liang, Nuno Faria, Josh Quick, Andrew Rambaut, Verity Hill, Wenjun Ma, Nick Loman, Oliver Pybus, Changwen Ke
**EPI_ISL_413870**	hCoV-19/Guangdong/FS-S48-P0047/2020	Asia / China / Guangdong	2020-02-28	Guangdong Provincial Institution of Public Health, Guangdong Provincial Center for Disease Control and Prevention	Guangdong Provincial Institution of Public Health	Jing Lu, Louis du Plessis, Liu Zhe, Jiufeng Sun, Sarah François, Huifang Lin, Moritz Kraemer, Jingju Peng, Qianlin Xiong, Runyu Yuan, Lilian Zeng, Pingping Zhou, Chuming Liang, Tao Liu, Wei Li, Juan Su, Huanying Zheng, Kang Min, Song Tie, Bo Peng, Shisong Fang, Wenzhe Su, Kuibiao Li, Ruilin Sun, Ru bai, Xi Tang, Minfeng Liang, Nuno Faria, Josh Quick, Andrew Rambaut, Verity Hill, Wenjun Ma, Nick Loman, Oliver Pybus, Changwen Ke
**EPI_ISL_413871**	hCoV-19/Guangdong/MM-S1-P0048/2020	Asia / China / Guangdong	2020-02-28	Guangdong Provincial Institution of Public Health, Guangdong Provincial Center for Disease Control and Prevention	Guangdong Provincial Institution of Public Health	Jing Lu, Louis du Plessis, Liu Zhe, Jiufeng Sun, Sarah François, Huifang Lin, Moritz Kraemer, Jingju Peng, Qianlin Xiong, Runyu Yuan, Lilian Zeng, Pingping Zhou, Chuming Liang, Tao Liu, Wei Li, Juan Su, Huanying Zheng, Kang Min, Song Tie, Bo Peng, Shisong Fang, Wenzhe Su, Kuibiao Li, Ruilin Sun, Ru bai, Xi Tang, Minfeng Liang, Nuno Faria, Josh Quick, Andrew Rambaut, Verity Hill, Wenjun Ma, Nick Loman, Oliver Pybus, Changwen Ke
**EPI_ISL_413872**	hCoV-19/Guangdong/SZ-N59-P0049/2020	Asia / China / Guangdong	2020-02-28	Guangdong Provincial Institution of Public Health, Guangdong Provincial Center for Disease Control and Prevention	Guangdong Provincial Institution of Public Health	Jing Lu, Louis du Plessis, Liu Zhe, Jiufeng Sun, Sarah François, Huifang Lin, Moritz Kraemer, Jingju Peng, Qianlin Xiong, Runyu Yuan, Lilian Zeng, Pingping Zhou, Chuming Liang, Tao Liu, Wei Li, Juan Su, Huanying Zheng, Kang Min, Song Tie, Bo Peng, Shisong Fang, Wenzhe Su, Kuibiao Li, Ruilin Sun, Ru bai, Xi Tang, Minfeng Liang, Nuno Faria, Josh Quick, Andrew Rambaut, Verity Hill, Wenjun Ma, Nick Loman, Oliver Pybus, Changwen Ke
**EPI_ISL_413873**	hCoV-19/Guangdong/GZ-S6-P0050/2020	Asia / China / Guangdong	2020-02-28	Guangdong Provincial Institution of Public Health, Guangdong Provincial Center for Disease Control and Prevention	Guangdong Provincial Institution of Public Health	Jing Lu, Louis du Plessis, Liu Zhe, Jiufeng Sun, Sarah François, Huifang Lin, Moritz Kraemer, Jingju Peng, Qianlin Xiong, Runyu Yuan, Lilian Zeng, Pingping Zhou, Chuming Liang, Tao Liu, Wei Li, Juan Su, Huanying Zheng, Kang Min, Song Tie, Bo Peng, Shisong Fang, Wenzhe Su, Kuibiao Li, Ruilin Sun, Ru bai, Xi Tang, Minfeng Liang, Nuno Faria, Josh Quick, Andrew Rambaut, Verity Hill, Wenjun Ma, Nick Loman, Oliver Pybus, Changwen Ke
**EPI_ISL_413874**	hCoV-19/Guangdong/GDFS2020127-P0026/2020	Asia / China / Guangdong	2020-02-12	Guangdong Provincial Institution of Public Health, Guangdong Provincial Center	Guangdong Provincial Institution of Public Health	Jing Lu, Louis du Plessis, Liu Zhe, Jiufeng Sun, Sarah François, Huifang Lin, Moritz Kraemer, Jingju Peng, Qianlin Xiong, Runyu Yuan, Lilian Zeng, Pingping Zhou, Chuming Liang, Tao Liu, Wei Li, Juan Su, Huanying Zheng, Kang Min, Song Tie, Bo Peng, Shisong Fang, Wenzhe Su, Kuibiao Li,

				for Disease Control and Prevention		Ruilin Sun, Ru bai, Xi Tang, Minfeng Liang, Nuno Faria, Josh Quick, Andrew Rambaut, Verity Hill, Wenjun Ma, Nick Loman, Oliver Pybus, Changwen Ke
**EPI_ISL_413875**	hCoV-19/Guangdong/GDFS2020052-P0025/2020	Asia / China / Guangdong	2020-02-10	Guangdong Provincial Institution of Public Health, Guangdong Provincial Center for Disease Control and Prevention	Guangdong Provincial Institution of Public Health	Jing Lu, Louis du Plessis, Liu Zhe, Jiufeng Sun, Sarah François, Huifang Lin, Moritz Kraemer, Jingju Peng, Qianlin Xiong, Runyu Yuan, Lilian Zeng, Pingping Zhou, Chuming Liang, Tao Liu, Wei Li, Juan Su, Huanying Zheng, Kang Min, Song Tie, Bo Peng, Shisong Fang, Wenzhe Su, Kuibiao Li, Ruilin Sun, Ru bai, Xi Tang, Minfeng Liang, Nuno Faria, Josh Quick, Andrew Rambaut, Verity Hill, Wenjun Ma, Nick Loman, Oliver Pybus, Changwen Ke
**EPI_ISL_413882**	hCoV-19/Guangdong/GD2020139-P0007/2020	Asia / China / Guangdong	2020-02-02	Guangdong Provincial Institution of Public Health, Guangdong Provincial Center for Disease Control and Prevention	Guangdong Provincial Institution of Public Health	Jing Lu, Louis du Plessis, Liu Zhe, Jiufeng Sun, Sarah François, Huifang Lin, Moritz Kraemer, Jingju Peng, Qianlin Xiong, Runyu Yuan, Lilian Zeng, Pingping Zhou, Chuming Liang, Tao Liu, Wei Li, Juan Su, Huanying Zheng, Kang Min, Song Tie, Bo Peng, Shisong Fang, Wenzhe Su, Kuibiao Li, Ruilin Sun, Ru bai, Xi Tang, Minfeng Liang, Nuno Faria, Josh Quick, Andrew Rambaut, Verity Hill, Wenjun Ma, Nick Loman, Oliver Pybus, Changwen Ke
**EPI_ISL_413883**	hCoV-19/Guangdong/GD2020134-P0031/2020	Asia / China / Guangdong	2020-02-02	Guangdong Provincial Institution of Public Health, Guangdong Provincial Center for Disease Control and Prevention	Guangdong Provincial Institution of Public Health	Jing Lu, Louis du Plessis, Liu Zhe, Jiufeng Sun, Sarah François, Huifang Lin, Moritz Kraemer, Jingju Peng, Qianlin Xiong, Runyu Yuan, Lilian Zeng, Pingping Zhou, Chuming Liang, Tao Liu, Wei Li, Juan Su, Huanying Zheng, Kang Min, Song Tie, Bo Peng, Shisong Fang, Wenzhe Su, Kuibiao Li, Ruilin Sun, Ru bai, Xi Tang, Minfeng Liang, Nuno Faria, Josh Quick, Andrew Rambaut, Verity Hill, Wenjun Ma, Nick Loman, Oliver Pybus, Changwen Ke
**EPI_ISL_413884**	hCoV-19/Guangdong/GD2020115-P0009/2020	Asia / China / Guangdong	2020-02-01	Guangdong Provincial Institution of Public Health, Guangdong Provincial Center for Disease Control and Prevention	Guangdong Provincial Institution of Public Health	Jing Lu, Louis du Plessis, Liu Zhe, Jiufeng Sun, Sarah François, Huifang Lin, Moritz Kraemer, Jingju Peng, Qianlin Xiong, Runyu Yuan, Lilian Zeng, Pingping Zhou, Chuming Liang, Tao Liu, Wei Li, Juan Su, Huanying Zheng, Kang Min, Song Tie, Bo Peng, Shisong Fang, Wenzhe Su, Kuibiao Li, Ruilin Sun, Ru bai, Xi Tang, Minfeng Liang, Nuno Faria, Josh Quick, Andrew Rambaut, Verity Hill, Wenjun Ma, Nick Loman, Oliver Pybus, Changwen Ke
**EPI_ISL_413888**	hCoV-19/Guangdong/2020XN4239-P0034/2020	Asia / China / Guangdong	2020-01-30	Guangdong Provincial Institution of Public Health, Guangdong Provincial Center for Disease Control and Prevention	Guangdong Provincial Institution of Public Health	Jing Lu, Louis du Plessis, Liu Zhe, Jiufeng Sun, Sarah François, Huifang Lin, Moritz Kraemer, Jingju Peng, Qianlin Xiong, Runyu Yuan, Lilian Zeng, Pingping Zhou, Chuming Liang, Tao Liu, Wei Li, Juan Su, Huanying Zheng, Kang Min, Song Tie, Bo Peng, Shisong Fang, Wenzhe Su, Kuibiao Li, Ruilin Sun, Ru bai, Xi Tang, Minfeng Liang, Nuno Faria, Josh Quick, Andrew Rambaut, Verity Hill, Wenjun Ma, Nick Loman, Oliver Pybus, Changwen Ke
**EPI_ISL_413889**	hCoV-19/Guangdong/2020XN4291-P0038/2020	Asia / China / Guangdong	2020-01-30	Guangdong Provincial Institution of Public Health, Guangdong Provincial Center for Disease Control and Prevention	Guangdong Provincial Institution of Public Health	Jing Lu, Louis du Plessis, Liu Zhe, Jiufeng Sun, Sarah François, Huifang Lin, Moritz Kraemer, Jingju Peng, Qianlin Xiong, Runyu Yuan, Lilian Zeng, Pingping Zhou, Chuming Liang, Tao Liu, Wei Li, Juan Su, Huanying Zheng, Kang Min, Song Tie, Bo Peng, Shisong Fang, Wenzhe Su, Kuibiao Li, Ruilin Sun, Ru bai, Xi Tang, Minfeng Liang, Nuno Faria, Josh Quick, Andrew Rambaut, Verity Hill, Wenjun Ma, Nick Loman, Oliver Pybus, Changwen Ke

Table S5. Acknowledgements table for the 500 genomed data set used in this study

Accession ID	Originating Laboratory	Submitting Laboratory	Authors
**EPI_ISL_407893**	Centre for Infectious Diseases and Microbiology Laboratory Services	NSW Health Pathology - Institute of Clinical Pathology and Medical Research; Westmead Hospital; University of Sydney	Eden J-S et al
**EPI_ISL_408976**	Centre for Infectious Diseases and Microbiology Laboratory Services	NSW Health Pathology - Institute of Clinical Pathology and Medical Research; Westmead Hospital; University of Sydney	Rockett R et al
**EPI_ISL_408977**	Serology, Virology and OTDS Laboratories (SAViD), NSW Health Pathology Randwick	NSW Health Pathology - Institute of Clinical Pathology and Medical Research; Centre for Infectious Diseases and Microbiology Laboratory Services; Westmead Hospital; University of Sydney	Eden J-S et al
**EPI_ISL_412975**	Centre for Infectious Diseases and Microbiology Laboratory Services	NSW Health Pathology - Institute of Clinical Pathology and Medical Research; Westmead Hospital; University of Sydney	Eden J-S et al
**EPI_ISL_413213**	Centre for Infectious Diseases and Microbiology Laboratory Services	NSW Health Pathology - Institute of Clinical Pathology and Medical Research; Westmead Hospital; University of Sydney	Eden J-S et al
**EPI_ISL_413594**	Centre for Infectious Diseases and Microbiology Laboratory Services	NSW Health Pathology - Institute of Clinical Pathology and Medical Research; Westmead Hospital; University of Sydney	Rockett R et al
**EPI_ISL_413596**	Centre for Infectious Diseases and Microbiology - Public Health	NSW Health Pathology - Institute of Clinical Pathology and Medical Research; Westmead Hospital; University of Sydney	Rockett R et al
**EPI_ISL_427657**	Centre for Infectious Diseases and Microbiology Public Health	NSW Health Pathology - Institute of Clinical Pathology and Medical Research; Westmead Hospital; University of Sydney	Rockett R et al
**EPI_ISL_413597**	Centre for Infectious Diseases and Microbiology- Public Health	NSW Health Pathology - Institute of Clinical Pathology and Medical Research; Westmead Hospital; University of Sydney	Lam C et al
**EPI_ISL_413598**	Centre for Infectious Diseases and Microbiology - Public Health	NSW Health Pathology - Institute of Clinical Pathology and Medical Research; Westmead Hospital; University of Sydney	Gray K et al
**EPI_ISL_413599**	Centre for Infectious Diseases and Microbiology - Public Health	NSW Health Pathology - Institute of Clinical Pathology and Medical Research; Westmead Hospital; University of Sydney	Timms et al
**EPI_ISL_427660**	Centre for Infectious Diseases and Microbiology Public Health	NSW Health Pathology - Institute of Clinical Pathology and Medical Research; Westmead Hospital; University of Sydney	Rockett R et al
**EPI_ISL_407894**	Pathology Queensland	Public Health Virology Laboratory	Ben Huang et al
**EPI_ISL_407896**	Pathology Queensland	Public Health Virology Laboratory	Ben Huang et al
**EPI_ISL_410717**	Pathology Queensland	Public Health Virology Laboratory	Ben Huang et al
**EPI_ISL_410718**	Pathology Queensland	Public Health Virology Laboratory	Ben Huang et al
**EPI_ISL_406844**	Monash Medical Centre	Collaboration between the University of Melbourne at The Peter Doherty Institute for Infection and Immunity, and the Victorian Infectious Disease Reference Laboratory	Caly et al
**EPI_ISL_419765**	Victorian Infectious Diseases Reference Laboratory (VIDRL)	Victorian Infectious Diseases Reference Laboratory and Microbiological Diagnostic Unit Public Health Laboratory, Doherty Institute	Caly L. et al
**EPI_ISL_420456**	PathWest Laboratory Medicine WA	PathWest Laboratory Medicine WA	Chisha Sikazwe et al
**EPI_ISL_430737**	Chinese PLA Institute for Disease Control and Prevention	Chinese PLA Institute for Disease Control and Prevention	Peng Li et al
**EPI_ISL_452361**	Laboratory of Infectious Diseases Center of Beijing Ditan Hospital	Laboratory of Infectious Diseases Center of Beijing Ditan Hospital	Siyuan Yang et al
**EPI_ISL_452345**	Laboratory of Infectious Diseases Center of Beijing Ditan Hospital	Laboratory of Infectious Diseases Center of Beijing Ditan Hospital	Siyuan Yang et al
**EPI_ISL_452332**	Laboratory of Infectious Diseases Center of Beijing Ditan Hospital	Laboratory of Infectious Diseases Center of Beijing Ditan Hospital	Siyuan Yang et al
**EPI_ISL_418800**	KU Leuven, Clinical and Epidemiological Virology	KU Leuven, Clinical and Epidemiological Virology	Bert Vanmechelen et al
**EPI_ISL_418983**	KU Leuven, Clinical and Epidemiological Virology	KU Leuven, Clinical and Epidemiological Virology	Bert Vanmechelen et al
**EPI_ISL_407976**	KU Leuven, Clinical and Epidemiological Virology	KU Leuven, Clinical and Epidemiological Virology	Bert Vanmechelen et al
**EPI_ISL_418798**	KU Leuven, Clinical and Epidemiological Virology	KU Leuven, Clinical and Epidemiological Virology	Bert Vanmechelen et al

**EPI_ISL_418982**	KU Leuven, Clinical and Epidemiological Virology	KU Leuven, Clinical and Epidemiological Virology	Bert Vanmechelen et al
**EPI_ISL_468729**	Lab voor klinische biologie	Onderzoeksgroep Virologie	Laurens Lambrechts et al
**EPI_ISL_417012**	Department of Clinical Microbiology	GIGA Medical Genomics	Durkin Keith et al
**EPI_ISL_412964**	Hospital Israelita Albert Einstein	Instituto Adolfo Lutz Interdisciplinary Procedures Center Strategic Laboratory	Jaqueline Goes de Jesus et al
**EPI_ISL_413016**	Hospital Israelita Albert Einstein	Instituto Adolfo Lutz, Interdisciplinary Procedures Center, Strategic Laboratory	Jaqueline Goes de Jesus et al
**EPI_ISL_411902**	Virology Unit, Institut Pasteur du Cambodge.	Virology Unit, Institut Pasteur du Cambodge (Sequencing done by: Jessica E Manning/Jennifer A Bohl at Malaria and Vector Research Research Laboratory, National Institute of Allergy and Infectious Diseases and Vida Ahyong from Chan-Zuckerberg Biohub)	Erik A Karlsson et al
**EPI_ISL_412965**	BCCDC Public Health Laboratory	BCCDC Public Health Laboratory	Harrigan et al
**EPI_ISL_415588**	BCCDC Public Health Laboratory	BCCDC Public Health Laboratory	Harrigan et al
**EPI_ISL_429806**	Dr. Georges-L.-Dumont University Hospital Centre	National Microbiology Laboratory	Anna Majer et al
**EPI_ISL_413014**	Public Health Ontario Laboratory	Ontario Agency for Health Protection and Promotion (OAHPP)	Alireza Eshaghi et al
**EPI_ISL_411957**	unknown	Key Laboratory of Human Diseases, Comparative Medicine, Institute of Laboratory Animal Science	Linlin et al
**EPI_ISL_408481**	National Institute for Viral Disease Control and Prevention, China CDC	National Institute for Viral Disease Control & Prevention, CCDC	Wenjie Tan et al
**EPI_ISL_408478**	Yongchuan District Center for Disease Control and Prevention	Chongqing Municipal Center for Disease Control and Prevention	Ye Sheng et al
**EPI_ISL_408479**	Zhongxian Center for Disease Control and Prevention	Chongqing Municipal Center for Disease Control and Prevention	Ye Sheng et al
**EPI_ISL_412116**	Respiratory Virus Unit, Microbiology Services Colindale, Public Health England	Respiratory Virus Unit, Microbiology Services Colindale, Public Health England	Monica Galiano et al
**EPI_ISL_464171**	Respiratory Virus Unit, Microbiology Services Colindale, Public Health England	Respiratory Virus Unit, Microbiology Services Colindale, Public Health England	PHE Covid Sequencing Team et al
**EPI_ISL_464176**	Respiratory Virus Unit, Microbiology Services Colindale, Public Health England	Respiratory Virus Unit, Microbiology Services Colindale, Public Health England	PHE Covid Sequencing Team et al
**EPI_ISL_464598**	Respiratory Virus Unit, Microbiology Services Colindale, Public Health England	Respiratory Virus Unit, Microbiology Services Colindale, Public Health England	PHE Covid Sequencing Team et al
**EPI_ISL_464888**	Respiratory Virus Unit, Microbiology Services Colindale, Public Health England	Respiratory Virus Unit, Microbiology Services Colindale, Public Health England	PHE Covid Sequencing Team et al
**EPI_ISL_464918**	Respiratory Virus Unit, Microbiology Services Colindale, Public Health England	Respiratory Virus Unit, Microbiology Services Colindale, Public Health England	PHE Covid Sequencing Team et al
**EPI_ISL_440178**	PHE South West Regional Laboratory, National Infection Service	Wellcome Sanger Institute for the COVID-19 Genomics UK Consortium	Stephanie Hutchings et al
**EPI_ISL_407079**	Lapland Central Hospital	Department of Virology, University of Helsinki and Helsinki University Hospital, Helsinki, Finland	Teemu Smura et al
**EPI_ISL_412971**	HUS Diagnostiikkakeskus, Hallinto	Department of Virology Faculty of Medicine, Medicum University of Helsinki	Teemu Smura et al
**EPI_ISL_414642**	Department of Virology and Immunology, University of Helsinki and Helsinki University Hospital, Huslab Finland	Department of Virology, Faculty of Medicine, University of Helsinki, Helsinki, Finland	Teemu Smura et al
**EPI_ISL_418219**	CHU - Hôpital Cavale Blanche - Labo. de Virologie	National Reference Center for Viruses of Respiratory Infections, Institut Pasteur, Paris	Mélanie Albert et al
**EPI_ISL_416511**	CHRU Pontchaillou - Laboratoire de Virologie	National Reference Center for Viruses of Respiratory Infections, Institut Pasteur, Paris	Mélnie Albert et al
**EPI_ISL_429968**	Centre Hospitalier Compiègne Laboratoire de Biologie	National Reference Center for Viruses of Respiratory Infections, Institut Pasteur, Paris	Mélanie Albert et al
**EPI_ISL_418218**	Centre Hospitalier Compiègne Laboratoire de Biologie	National Reference Center for Viruses of Respiratory Infections, Institut Pasteur, Paris	Mélanie Albert et al
**EPI_ISL_415654**	Centre Hospitalier Compiègne Laboratoire de Biologie	National Reference Center for Viruses of Respiratory Infections, Institut Pasteur, Paris	Mélnie Albert et al
**EPI_ISL_416495**	Centre Hospitalier Compiègne Laboratoire de Biologie	National Reference Center for Viruses of Respiratory Infections, Institut Pasteur, Paris	Mélnie Albert et al
**EPI_ISL_418226**	EHPAD - Résidences les Cèdres	National Reference Center for Viruses of Respiratory Infections, Institut Pasteur, Paris	Mélanie Albert et al

**EPI_ISL_406597**	Department of Infectious and Tropical Diseases, Bichat Claude Bernard Hospital, Paris	National Reference Center for Viruses of Respiratory Infections, Institut Pasteur, Paris	Mélanie Albert et al
**EPI_ISL_411219**	Department of Infectious and Tropical Diseases, Bichat Claude Bernard Hospital, Paris	Laboratoire Virpath, CIRI U111, UCBL1, INSERM, CNRS, ENS Lyon	Olivier Terrier et al
**EPI_ISL_408430**	Department of Infectious and Tropical Diseases, Bichat Claude Bernard Hospital, Paris	National Reference Center for Viruses of Respiratory Infections, Institut Pasteur, Paris	Mélanie Albert et al
**EPI_ISL_417335**	Institut des Agents Infectieux (IAI), Hospices Civils de Lyon	CNR Virus des Infections Respiratoires - France SUD	Antonin Bal et al
**EPI_ISL_416745**	CNR Virus des Infections Respiratoires - France SUD	CNR Virus des Infections Respiratoires - France SUD	Bal et al
**EPI_ISL_410486**	CNR Virus des Infections Respiratoires - France SUD	CNR Virus des Infections Respiratoires - France SUD	Bal et al
**EPI_ISL_424352**	unknown	Clinical Laboratory	Ge et al
**EPI_ISL_455465**	Jiangxi Province Center for Disease Control and Prevention	Jiangxi Province Center for Disease Control and Prevention	JianXiong Li et al
**EPI_ISL_437244**	Max von Pettenkofer Institute, Virology, National Reference Center for Retroviruses, LMU München	Laboratory for Functional Genome Analysis, Dept. Genomics, Gene Center of the LMU Munich	Max Muenchhoff et al
**EPI_ISL_412912**	State Health Office Baden-Wuerttemberg	Charité Universitätsmedizin Berlin, Institute of Virology	Victor M Corman et al
**EPI_ISL_406862**	Charité Universitätsmedizin Berlin, Institute of Virology; Institut für Mikrobiologie der Bundeswehr, Munich	Charité Universitätsmedizin Berlin, Institute of Virology	Victor M Corman et al
**EPI_ISL_452218**	University Hospital Frankfurt	Widera/Toptan	Tuna Toptan1 et al
**EPI_ISL_452220**	University Hospital Frankfurt	Widera/Toptan	Tuna Toptan1 et al
**EPI_ISL_417457**	Center of Medical Microbiology, Virology, and Hospital Hygiene, University of Duesseldorf	Center of Medical Microbiology, Virology, and Hospital Hygiene, University of Duesseldorf	Ortwin Adams et al
**EPI_ISL_425133**	Center of Medical Microbiology, Virology, and Hospital Hygiene, University of Duesseldorf	Center of Medical Microbiology, Virology, and Hospital Hygiene, University of Duesseldorf	Ortwin Adams et al
**EPI_ISL_413860**	Guangdong Provincial Institution of Public Health, Guangdong Provinical Center for Disease Control and Prevention	Guangdong Provincial Institution of Public Health	Jing Lu et al
**EPI_ISL_403932**	Guangdong Provincial Center for Diseases Control and Prevention; Guangdong Provincial Public Health	Department of Microbiology, Guangdong Provincial Center for Diseases Control and Prevention	Min Kang et al
**EPI_ISL_403934**	Guangdong Provincial Center for Diseases Control and Prevention; Guangdong Provincial Public Health	Department of Microbiology, Guangdong Provincial Center for Diseases Control and Prevention	Min Kang et al
**EPI_ISL_403936**	Guangdong Provincial Center for Diseases Control and Prevention; Guangdong Provincial Public Health	Department of Microbiology, Guangdong Provincial Center for Diseases Control and Prevention	Min Kang et al
**EPI_ISL_403937**	Guangdong Provincial Center for Diseases Control and Prevention; Guangdong Provincial Public Health	Department of Microbiology, Guangdong Provincial Center for Diseases Control and Prevention	Min Kang et al
**EPI_ISL_428440**	Guangdong Provincial Center for Diseases Control and Prevention;Guangdong Provincial Institute of Public Health	School of Public Health, The University of Hong Kong	Bosheng Li et al
**EPI_ISL_428443**	Guangdong Provincial Center for Diseases Control and Prevention;Guangdong Provincial Institute of Public Health	School of Public Health, The University of Hong Kong	Bosheng Li et al
**EPI_ISL_428447**	Guangdong Provincial Center for Diseases Control and Prevention;Guangdong Provincial Institute of Public Health	School of Public Health, The University of Hong Kong	Bosheng Li et al
**EPI_ISL_428462**	Guangdong Provincial Center for Diseases Control and Prevention;Guangdong Provincial Institute of Public Health	School of Public Health, The University of Hong Kong	Bosheng Li et al
**EPI_ISL_428463**	Guangdong Provincial Center for Diseases Control and Prevention;Guangdong Provincial Institute of Public Health	School of Public Health, The University of Hong Kong	Bosheng Li et al
**EPI_ISL_413861**	Guangdong Provincial Institution of Public Health, Guangdong Provinical Center for Disease Control and Prevention	Guangdong Provincial Institution of Public Health	Jing Lu et al

**EPI_ISL_413884**	Guangdong Provincial Institution of Public Health, Guangdong Provinical Center for Disease Control and Prevention	Guangdong Provincial Institution of Public Health	Jing Lu et al
**EPI_ISL_413864**	Guangdong Provincial Institution of Public Health, Guangdong Provinical Center for Disease Control and Prevention	Guangdong Provincial Institution of Public Health	Jing Lu et al
**EPI_ISL_413875**	Guangdong Provincial Institution of Public Health, Guangdong Provinical Center for Disease Control and Prevention	Guangdong Provincial Institution of Public Health	Jing Lu et al
**EPI_ISL_413850**	Guangdong Provincial Institution of Public Health, Guangdong Provinical Center for Disease Control and Prevention	Guangdong Provincial Institution of Public Health	Jing Lu et al
**EPI_ISL_413874**	Guangdong Provincial Institution of Public Health, Guangdong Provinical Center for Disease Control and Prevention	Guangdong Provincial Institution of Public Health	Jing Lu et al
**EPI_ISL_454418**	unknown	Research and Experiment Center	Guo et al
**EPI_ISL_429076**	The First Affiliated Hospital of Guangzhou Medical University	BGI-shenzhen & The First Affiliated Hospital of Guangzhou Medical University	et al
**EPI_ISL_429078**	The First Affiliated Hospital of Guangzhou Medical University	BGI-shenzhen & The First Affiliated Hospital of Guangzhou Medical University	et al
**EPI_ISL_429081**	The First Affiliated Hospital of Guangzhou Medical University	BGI-shenzhen & The First Affiliated Hospital of Guangzhou Medical University	et al
**EPI_ISL_429082**	The First Affiliated Hospital of Guangzhou Medical University	BGI-shenzhen & The First Affiliated Hospital of Guangzhou Medical University	Yanqun Wang et al
**EPI_ISL_429084**	The First Affiliated Hospital of Guangzhou Medical University	BGI-shenzhen & The First Affiliated Hospital of Guangzhou Medical University	et al
**EPI_ISL_414686**	State Key Laboratory of Respiratory Disease, National Clinical Research Center for Respiratory Disease, Guangzhou Institute of Respiratory Health, the First Affiliated Hospital of Guangzhou Medical University	The First Affiliated Hospital of Guangzhou Medical University & BGI-Shenzhen	Zhao et al et al
**EPI_ISL_429085**	The First Affiliated Hospital of Guangzhou Medical University	BGI-shenzhen & The First Affiliated Hospital of Guangzhou Medical University	Yanqun Wang et al
**EPI_ISL_429086**	The First Affiliated Hospital of Guangzhou Medical University	BGI-shenzhen & The First Affiliated Hospital of Guangzhou Medical University	et al
**EPI_ISL_429090**	The First Affiliated Hospital of Guangzhou Medical University	BGI-shenzhen & The First Affiliated Hospital of Guangzhou Medical University	Yanqun Wang et al
**EPI_ISL_429091**	The First Affiliated Hospital of Guangzhou Medical University	BGI-shenzhen & The First Affiliated Hospital of Guangzhou Medical University	Yanqun Wang et al
**EPI_ISL_414688**	State Key Laboratory of Respiratory Disease, National Clinical Research Center for Respiratory Disease, Guangzhou Institute of Respiratory Health, the First Affiliated Hospital of Guangzhou Medical University	The First Affiliated Hospital of Guangzhou Medical University & BGI-Shenzhen	Zhao et al et al
**EPI_ISL_429093**	The First Affiliated Hospital of Guangzhou Medical University	BGI-shenzhen & The First Affiliated Hospital of Guangzhou Medical University	Yanqun Wang et al
**EPI_ISL_429095**	The First Affiliated Hospital of Guangzhou Medical University	BGI-shenzhen & The First Affiliated Hospital of Guangzhou Medical University	et al
**EPI_ISL_429096**	The First Affiliated Hospital of Guangzhou Medical University	BGI-shenzhen & The First Affiliated Hospital of Guangzhou Medical University	Yanqun Wang et al
**EPI_ISL_429099**	The First Affiliated Hospital of Guangzhou Medical University	BGI-shenzhen & The First Affiliated Hospital of Guangzhou Medical University	et al
**EPI_ISL_429101**	The First Affiliated Hospital of Guangzhou Medical University	BGI-shenzhen & The First Affiliated Hospital of Guangzhou Medical University	et al
**EPI_ISL_429103**	The First Affiliated Hospital of Guangzhou Medical University	BGI-shenzhen & The First Affiliated Hospital of Guangzhou Medical University	et al
**EPI_ISL_457687**	The First Affiliated Hospital of Guangzhou Medical University, Guangzhou, China	BGI-shenzhen & The First Affiliated Hospital of Guangzhou Medical University	Yanqun Wang et al
**EPI_ISL_457688**	The First Affiliated Hospital of Guangzhou Medical University, Guangzhou, China	BGI-shenzhen & The First Affiliated Hospital of Guangzhou Medical University	Yanqun Wang et al
**EPI_ISL_457689**	The First Affiliated Hospital of Guangzhou Medical University, Guangzhou, China	BGI-shenzhen & The First Affiliated Hospital of Guangzhou Medical University	Yanqun Wang et al
**EPI_ISL_457690**	The First Affiliated Hospital of Guangzhou Medical University, Guangzhou, China	BGI-shenzhen & The First Affiliated Hospital of Guangzhou Medical University	Yanqun Wang et al

**EPI_ISL_457691**	The First Affiliated Hospital of Guangzhou Medical University, Guangzhou, China	BGI-shenzhen & The First Affiliated Hospital of Guangzhou Medical University	Yanqun Wang et al
**EPI_ISL_457692**	The First Affiliated Hospital of Guangzhou Medical University, Guangzhou, China	BGI-shenzhen & The First Affiliated Hospital of Guangzhou Medical University	Yanqun Wang et al
**EPI_ISL_457693**	The First Affiliated Hospital of Guangzhou Medical University, Guangzhou, China	BGI-shenzhen & The First Affiliated Hospital of Guangzhou Medical University	Yanqun Wang et al
**EPI_ISL_457694**	The First Affiliated Hospital of Guangzhou Medical University, Guangzhou, China	BGI-shenzhen & The First Affiliated Hospital of Guangzhou Medical University	Yanqun Wang et al
**EPI_ISL_457695**	The First Affiliated Hospital of Guangzhou Medical University, Guangzhou, China	BGI-shenzhen & The First Affiliated Hospital of Guangzhou Medical University	Yanqun Wang et al
**EPI_ISL_457696**	The First Affiliated Hospital of Guangzhou Medical University, Guangzhou, China	BGI-shenzhen & The First Affiliated Hospital of Guangzhou Medical University	Yanqun Wang et al
**EPI_ISL_406970**	Hangzhou Center for Disease and Control Microbiology Lab	Hangzhou Center for Disease and Control Microbiology Lab	Yu Hua et al
**EPI_ISL_418510**	Hangzhou Center for Disease Control and Prevention	Insepction Center of Hangzhou Center for Disease Control and Prevention	Yu hua et al
**EPI_ISL_418441**	Hangzhou Center for Disease Control and Prevention	Inspection Center of Hangzhou Center for Disease Control and Prevention	Yu hua et al
**EPI_ISL_418442**	Hangzhou Center for Disease Control and Prevention	Inspection Center of Hangzhou Center for Disease Control and Prevention	Yu hua et al
**EPI_ISL_418513**	Hangzhou Center for Disease Control and Prevention	Inspection Center of Hangzhou Center for Disease Control and Prevention	Yu hua et al
**EPI_ISL_418502**	Hangzhou Center for Disease Control and Prevention	Inspection Center of Hangzhou Center for Disease Control and Prevention	Yu hua et al
**EPI_ISL_418503**	Hangzhou Center for Disease Control and Prevention	Inspection Center of Hangzhou Center for Disease Control and Prevention	Yu hua et al
**EPI_ISL_418515**	Hangzhou Center for Disease Control and Prevention	Inspection Center of Hangzhou Center for Disease Control and Prevention	Yu hua et al
**EPI_ISL_418504**	Hangzhou Center for Disease Control and Prevention	Inspection Center of Hangzhou Center for Disease Control and Prevention	Yu hua et al
**EPI_ISL_418506**	Hangzhou Center for Disease Control and Prevention	Inspection Center of Hanghzou Center for Disease Control and Prevention	Yu hua et al
**EPI_ISL_407313**	Hangzhou Center for Disease Control and Prevention	Hangzhou Center for Disease Control and Prevention	Jun Li et al
**EPI_ISL_421236**	Hangzhou Center for Diseases Control and Prevention	Hangzhou Center for Diseases Control and Prevention	Jun Li et al
**EPI_ISL_421234**	Hangzhou Center for Diseases Control and Prevention	Hangzhou Center for Diseases Control and Prevention	Jun Li et al
**EPI_ISL_421233**	Hangzhou Center for Diseases Control and Prevention	Hangzhou Center for Diseases Control and Prevention	Jun Li et al
**EPI_ISL_421232**	Hangzhou Center for Diseases Control and Prevention	Hangzhou Center for Diseases Control and Prevention	Jun Li et al
**EPI_ISL_421231**	Hangzhou Center for Diseases Control and Prevention	Hangzhou Center for Diseases Control and Prevention	Jun Li et al
**EPI_ISL_421229**	Hangzhou Center for Diseases Control and Prevention	Hangzhou Center for Diseases Control and Prevention	Jun Li et al
**EPI_ISL_421228**	Hangzhou Center for Diseases Control and Prevention	Hangzhou Center for Diseases Control and Prevention	Jun Li et al
**EPI_ISL_421227**	Hangzhou Center for Diseases Control and Prevention	Hangzhou Center for Diseases Control and Prevention	Jun Li et al
**EPI_ISL_421226**	Hangzhou Center for Diseases Control and Prevention	Hangzhou Center for Diseases Control and Prevention	Jun Li et al
**EPI_ISL_421225**	Hangzhou Center for Diseases Control and Prevention	Hangzhou Center for Diseases Control and Prevention	Jun Li et al
**EPI_ISL_421224**	Hangzhou Center for Diseases Control and Prevention	Hangzhou Center for Diseases Control and Prevention	Jun Li et al
**EPI_ISL_482575**	Hangzhou Center for Diseases Control and Prevention	Hangzhou Center for Diseases Control and Prevention	Jun Li et al
**EPI_ISL_482577**	Hangzhou Center for Diseases Control and Prevention	Hangzhou Center for Diseases Control and Prevention	Jun Li et al
**EPI_ISL_415709**	State Key Laboratory for Diagnosis and Treatment of Infectious Diseases, National Clinical Research Center for Infectious Diseases, First Affiliated Hospital, Zhejiang University School of Medicine, Hangzhou, China. 310003	State Key Laboratory for Diagnosis and Treatment of Infectious Diseases, National Clinical Research Center for Infectious Diseases, First Affiliated Hospital, Zhejiang University School of Medicine, Hangzhou, China. 310003	Hangping Yao et al
**EPI_ISL_418991**	State Key Laboratory for Diagnosis and Treatment of Infectious Diseases, National Clinical Research Center for Infectious Diseases, First Affiliated	State Key Laboratory for Diagnosis and Treatment of Infectious Diseases, National Clinical Research Center for Infectious Diseases, First Affiliated Hospital, Zhejiang	Hangping Yao et al

	Hospital, Zhejiang University School of Medicine, Hangzhou, China 310003	University School of Medicine, Hangzhou, China 310003	
**EPI_ISL_416042**	State Key Laboratory for Diagnosis and Treatment of Infectious Diseases, National Clinical Research Center for Infectious Diseases, First Affiliated Hospital, Zhejiang University School of Medicine, Hangzhou, China. 310003	State Key Laboratory for Diagnosis and Treatment of Infectious Diseases, National Clinical Research Center for Infectious Diseases, First Affiliated Hospital, Zhejiang University School of Medicine, Hangzhou, China. 310003	Hangping Yao et al
**EPI_ISL_416046**	State Key Laboratory for Diagnosis and Treatment of Infectious Diseases, National Clinical Research Center for Infectious Diseases, First Affiliated Hospital, Zhejiang University School of Medicine, Hangzhou, China 310003	State Key Laboratory for Diagnosis and Treatment of Infectious Diseases, National Clinical Research Center for Infectious Diseases, First Affiliated Hospital, Zhejiang University School of Medicine, Hangzhou, China 310003	Hangping Yao et al
**EPI_ISL_415711**	State Key Laboratory for Diagnosis and Treatment of Infectious Diseases, National Clinical Research Center for Infectious Diseases, First Affiliated Hospital, Zhejiang University School of Medicine, Hangzhou, China. 310003	State Key Laboratory for Diagnosis and Treatment of Infectious Diseases, National Clinical Research Center for Infectious Diseases, First Affiliated Hospital, Zhejiang University School of Medicine, Hangzhou, China. 310003	Hangping Yao et al
**EPI_ISL_416425**	State Key Laboratory for Diagnosis and Treatment of Infectious Diseases, National Clinical Research Center for Infectious Diseases, First Affiliated Hospital, Zhejiang University School of Medicine, Hangzhou, China 310003	State Key Laboratory for Diagnosis and Treatment of Infectious Diseases, National Clinical Research Center for Infectious Diseases, First Affiliated Hospital, Zhejiang University School of Medicine, Hangzhou, China 310003	Hangping Yao et al
**EPI_ISL_416474**	State Key Laboratory for Diagnosis and Treatment of Infectious Diseases, National Clinical Research Center for Infectious Diseases, First Affiliated Hospital, Zhejiang University School of Medicine, Hangzhou, China 310003	State Key Laboratory for Diagnosis and Treatment of Infectious Diseases, National Clinical Research Center for Infectious Diseases, First Affiliated Hospital, Zhejiang University School of Medicine, Hangzhou, China 310003	Hangping Yao et al
**EPI_ISL_450442**	unknown	The Department of Infectious Disease Prevention and Control	Li et al
**EPI_ISL_420455**	Department of Clinical Pathology, Pamela Youde Nethersole Eastern Hospital	Department of Health Technology and Informatics, Faculty of Health and Social Science, The Hong Kong Polytechnic University	Kenneth Siu-Sing LEUNG et al
**EPI_ISL_418815**	Department of Clinical Pathology, Pamela Youde Nethersole Eastern Hospital	Department of Health Technology and Informatics, Faculty of Health and Social Science, The Hong Kong Polytechnic University	Kenneth Siu-Sing LEUNG et al
**EPI_ISL_419219**	Department of Clinical Pathology, Pamela Youde Nethersole Eastern Hospital	Department of Health Technology and Informatics, Faculty of Health and Social Science, The Hong Kong Polytechnic University	Kenneth Siu-Sing LEUNG et al
**EPI_ISL_419223**	Department of Pathology, Princess Margaret Hospital	Department of Health Technology and Informatics, Faculty of Health and Social Science, The Hong Kong Polytechnic University	Kenneth Siu-Sing LEUNG et al
**EPI_ISL_412029**	Hong Kong Department of Health	The University of Hong Kong	Dominic N.C. Tsang et al
**EPI_ISL_414571**	Hong Kong Department of Health	School of Public Health, The University of Hong Kong	Dominic N.C. Tsang et al
**EPI_ISL_450405**	unknown	School of Public Health	Sit et al
**EPI_ISL_413522**	Indian Council of Medical Research - National Institute of Virology	National Influenza Center, Indian Council of Medical Research - National Institute of Virology	Potdar V et al
**EPI_ISL_413523**	Indian Council of Medical Research-National Institute of Virology	National Influenza Center, Indian Council of Medical Research-National Institute of Virology	Potdar V et al
**EPI_ISL_421665**	National Influenza Center, Indian Council of Medical Research - National Institute of Virology	Indian Council of Medical Research-National Institute of Virology, Microbial Containment Complex	Pragya D. Yadav et al
**EPI_ISL_431101**	Department of Microbiology,Gandhi Medical College and Hospital	Virus Research Laboratory, Department of Zoology, Osmania University,Hyderabad,India	Muttineni Radhakrishna et al
**EPI_ISL_452213**	NIV Influenza	NIV Influenza	Potdar V et al
**EPI_ISL_426179**	National Influenza Center, Indian Council of Medical Research - National Institute of Virology	Indian Council of Medical Research-National Institute of Virology, Microbial Containment Complex	Pragya D. Yadav et al
**EPI_ISL_424349**	unknown	Human Genetic Research Center	Zeinali et al
**EPI_ISL_457699**	Department of Infectious Diseases, Istituto Superiore di Sanità, Roma , Italy	Army Medical and Veterinary Research Center	Paola Stefanelli et al
**EPI_ISL_457728**	Department of Infectious Diseases, Istituto Superiore di Sanità, Roma , Italy	Army Medical and Veterinary Research Center	Paola Stefanelli et al

**EPI_ISL_457749**	Department of Infectious Diseases, Istituto Superiore di Sanità, Roma , Italy	Army Medical and Veterinary Research Center	Paola Stefanelli et al
**EPI_ISL_457826**	Army Medical Center - Scientific Department	Army Medical and Veterinary Research Center	Silvia Fillo et al
**EPI_ISL_460080**	Molecular Virology Unit, Fondazione IRCCS Policlinico San Matteo , Pavia	Laboratory of Virology, INMI Lazzaro Spallanzani IRCCS	Antonio Piralla et al
**EPI_ISL_460079**	Molecular Virology Unit, Fondazione IRCCS Policlinico San Matteo , Pavia	Laboratory of Virology, INMI Lazzaro Spallanzani IRCCS	Barbara Bartolini et al
**EPI_ISL_460083**	Molecular Virology Unit, Fondazione IRCCS Policlinico San Matteo , Pavia	Laboratory of Virology, INMI Lazzaro Spallanzani IRCCS	Martina Rueca et al
**EPI_ISL_412973**	Department of Infectious Diseases, Istituto Superiore di Sanità, Roma , Italy	Virology Laboratory, Scientific Department, Army Medical Center	Paola Stefanelli et al
**EPI_ISL_479791**	Laboratory of Molecular Virology of the International Centre for Genetic Engineering and Biotechnology (ICGEB)	ARGO Open Lab Platform for Genome Sequencing	Licastro et al
**EPI_ISL_417419**	Laboratory of Molecular Virology International Center for Genetic Engineering and Biotechnology (ICGEB)	ARGO Open Lab Platform for Genome sequencing	Licastro D et al
**EPI_ISL_451299**	Laboratory of Virology, INMI Lazzaro Spallanzani IRCCS	Laboratory of Virology, INMI Lazzaro Spallanzani IRCCS	Martina Rueca et al
**EPI_ISL_410545**	INMI Lazzaro Spallanzani IRCCS	Laboratory of Virology, INMI Lazzaro Spallanzani IRCCS	Maria R. Capobianchi et al
**EPI_ISL_451301**	Laboratory of Virology, INMI Lazzaro Spallanzani IRCCS	Laboratory of Virology, INMI Lazzaro Spallanzani IRCCS	Martina Rueca et al
**EPI_ISL_451309**	Molecular Virology Unit, Fondazione IRCCS Policlinico San Matteo , Pavia	Laboratory of Virology, INMI Lazzaro Spallanzani IRCCS	Fausto Baldanti et al
**EPI_ISL_460091**	Molecular Virology Unit, Fondazione IRCCS Policlinico San Matteo , Pavia	Laboratory of Virology, INMI Lazzaro Spallanzani IRCCS	Antonino Di Caro et al
**EPI_ISL_460092**	Molecular Virology Unit, Fondazione IRCCS Policlinico San Matteo , Pavia	Laboratory of Virology, INMI Lazzaro Spallanzani IRCCS	Cesare E.M. Gruber et al
**EPI_ISL_451306**	Molecular Virology Unit, Fondazione IRCCS Policlinico San Matteo , Pavia	Laboratory of Virology, INMI Lazzaro Spallanzani IRCCS	Antonio Piralla et al
**EPI_ISL_451307**	Molecular Virology Unit, Fondazione IRCCS Policlinico San Matteo , Pavia	Laboratory of Virology, INMI Lazzaro Spallanzani IRCCS	Fausto Baldanti et al
**EPI_ISL_460094**	Molecular Virology Unit, Fondazione IRCCS Policlinico San Matteo , Pavia	Laboratory of Virology, INMI Lazzaro Spallanzani IRCCS	Barbara Bartolini et al
**EPI_ISL_412974**	Department of Infectious Diseases, Istituto Superiore di Sanità, Rome, Italy	Virology Laboratory, Scientific Department, Army Medical Center	Paola Stefanelli et al
**EPI_ISL_417445**	Laboratory of Infectious Diseases, Department of Biomedical and Clinical Sciences L. Sacco, University of Milan	Laboratory of Infectious Diseases, Department of Biomedical and Clinical Sciences L. Sacco, University of Milan	Gianguglielmo Zehender et al
**EPI_ISL_417491**	Virology Laboratory, Department of Biomedical Sciences and Public Health, University Politecnica delle Marche	Virology and Legal Medicine Laboratories, Department of Biomedical Sciences and Public Health, University Politecnica delle Marche	Bagnarelli et al
**EPI_ISL_416568**	Japanese Quarantine Stations	Pathogen Genomics Center, National Institute of Infectious Diseases	Tsuyoshi Sekizuka et al
**EPI_ISL_416572**	Japanese Quarantine Stations	Pathogen Genomics Center, National Institute of Infectious Diseases	Tsuyoshi Sekizuka et al
**EPI_ISL_416577**	Japanese Quarantine Stations	Pathogen Genomics Center, National Institute of Infectious Diseases	Tsuyoshi Sekizuka et al
**EPI_ISL_416627**	Japanese Quarantine Stations	Pathogen Genomics Center, National Institute of Infectious Diseases	Tsuyoshi Sekizuka et al
**EPI_ISL_410531**	Dept. of Pathology, National Institute of Infectious Diseases	Pathogen Genomics Center, National Institute of Infectious Diseases	Tsuyoshi Sekizuka et al
**EPI_ISL_479803**	Sagamihara City Public Health Research Institute	Pathogen Genomics Center, National Institute of Infectious Diseases	Tsuyoshi Sekizuka et al
**EPI_ISL_479822**	Department of Infectious Diseases, Kobe Institute of Health	Pathogen Genomics Center, National Institute of Infectious Diseases	Tsuyoshi Sekizuka et al
**EPI_ISL_479928**	Saitama Prefectural Institute of Public Health	Pathogen Genomics Center, National Institute of Infectious Diseases	Tsuyoshi Sekizuka et al
**EPI_ISL_479930**	Saitama Prefectural Institute of Public Health	Pathogen Genomics Center, National Institute of Infectious Diseases	Tsuyoshi Sekizuka et al
**EPI_ISL_480052**	Nagoya City Public Health Research Institute	Pathogen Genomics Center, National Institute of Infectious Diseases	Tsuyoshi Sekizuka et al
**EPI_ISL_408488**	National Institute for Viral Disease Control and Prevention, China CDC	National Institute for Viral Disease Control & Prevention, CCDC	Wenjie Tan et al
**EPI_ISL_411952**	NHC Key laboratory of Enteric Pathogenic Microbiology, Institute of Pathogenic Microbiology	Jiangsu Provincial Center for Disease Control & Prevention	Kangchen Zhao et al
**EPI_ISL_411953**	NHC Key laboratory of Enteric Pathogenic Microbiology, Institute of Pathogenic Microbiology	Jiangsu Provincial Center for Disease Control & Prevention	Kangchen Zhao et al
**EPI_ISL_408486**	National Institute for Viral Disease Control and Prevention, China CDC	National Institute for Viral Disease Control & Prevention, CCDC	Wenjie Tan et al

**EPI_ISL_412459**	Jingzhou Center for Disease Control and Prevention	Hubei Provincial Center for Disease Control and Prevention	Bin Fang et al
**EPI_ISL_455463**	Jiangxi Province Center for Disease Control and Prevention	Jiangxi Province Center for Disease Control and Prevention	JianXiong Li et al
**EPI_ISL_425118**	Division of Viral Diseases, Center for Laboratory Control of Infectious Diseases, Korea Centers for Diseases Control and Prevention	Division of Viral Diseases, Center for Laboratory Control of Infectious Diseases, Korea Centers for Diseases Control and Prevention	Jeong-Min Kim et al
**EPI_ISL_426171**	Division of Viral Diseases, Center for Laboratory Control of Infectious Diseases, Korea Centers for Diseases Control and Prevention	Division of Viral Diseases, Center for Laboratory Control of Infectious Diseases, Korea Centers for Diseases Control and Prevention	Jeong-Min Kim et al
**EPI_ISL_426173**	Division of Viral Diseases, Center for Laboratory Control of Infectious Diseases, Korea Centers for Diseases Control and Prevention	Division of Viral Diseases, Center for Laboratory Control of Infectious Diseases, Korea Centers for Diseases Control and Prevention	Jeong-Min Kim et al
**EPI_ISL_426180**	Division of Viral Diseases, Center for Laboratory Control of Infectious Diseases, Korea Centers for Diseases Control and Prevention	Division of Viral Diseases, Center for Laboratory Control of Infectious Diseases, Korea Centers for Diseases Control and Prevention	Jeong-Min Kim et al
**EPI_ISL_426181**	Division of Viral Diseases, Center for Laboratory Control of Infectious Diseases, Korea Centers for Diseases Control and Prevention	Division of Viral Diseases, Center for Laboratory Control of Infectious Diseases, Korea Centers for Diseases Control and Prevention	Jeong-Min Kim et al
**EPI_ISL_426182**	Division of Viral Diseases, Center for Laboratory Control of Infectious Diseases, Korea Centers for Diseases Control and Prevention	Division of Viral Diseases, Center for Laboratory Control of Infectious Diseases, Korea Centers for Diseases Control and Prevention	Jeong-Min Kim et al
**EPI_ISL_426187**	Division of Viral Diseases, Center for Laboratory Control of Infectious Diseases, Korea Centers for Diseases Control and Prevention	Division of Viral Diseases, Center for Laboratory Control of Infectious Diseases, Korea Centers for Diseases Control and Prevention	Jeong-Min Kim et al
**EPI_ISL_471426**	Division of Viral Diseases, Center for Laboratory Control of Infectious Diseases, Korea Centers for Diseases Control and Prevention	Division of Viral Diseases, Center for Laboratory Control of Infectious Diseases, Korea Centers for Diseases Control and Prevention	Jeong-Min Kim et al
**EPI_ISL_471440**	Division of Viral Diseases, Center for Laboratory Control of Infectious Diseases, Korea Centers for Diseases Control and Prevention	Division of Viral Diseases, Center for Laboratory Control of Infectious Diseases, Korea Centers for Diseases Control and Prevention	Jeong-Min Kim et al
**EPI_ISL_471444**	Division of Viral Diseases, Center for Laboratory Control of Infectious Diseases, Korea Centers for Diseases Control and Prevention	Division of Viral Diseases, Center for Laboratory Control of Infectious Diseases, Korea Centers for Diseases Control and Prevention	Jeong-Min Kim et al
**EPI_ISL_471446**	Division of Viral Diseases, Center for Laboratory Control of Infectious Diseases, Korea Centers for Diseases Control and Prevention	Division of Viral Diseases, Center for Laboratory Control of Infectious Diseases, Korea Centers for Diseases Control and Prevention	Jeong-Min Kim et al
**EPI_ISL_471447**	Division of Viral Diseases, Center for Laboratory Control of Infectious Diseases, Korea Centers for Diseases Control and Prevention	Division of Viral Diseases, Center for Laboratory Control of Infectious Diseases, Korea Centers for Diseases Control and Prevention	Jeong-Min Kim et al
**EPI_ISL_471448**	Division of Viral Diseases, Center for Laboratory Control of Infectious Diseases, Korea Centers for Diseases Control and Prevention	Division of Viral Diseases, Center for Laboratory Control of Infectious Diseases, Korea Centers for Diseases Control and Prevention	Jeong-Min Kim et al
**EPI_ISL_471451**	Division of Viral Diseases, Center for Laboratory Control of Infectious Diseases, Korea Centers for Diseases Control and Prevention	Division of Viral Diseases, Center for Laboratory Control of Infectious Diseases, Korea Centers for Diseases Control and Prevention	Jeong-Min Kim et al
**EPI_ISL_471453**	Division of Viral Diseases, Center for Laboratory Control of Infectious Diseases, Korea Centers for Diseases Control and Prevention	Division of Viral Diseases, Center for Laboratory Control of Infectious Diseases, Korea Centers for Diseases Control and Prevention	Jeong-Min Kim et al
**EPI_ISL_471454**	Division of Viral Diseases, Center for Laboratory Control of Infectious Diseases, Korea Centers for Diseases Control and Prevention	Division of Viral Diseases, Center for Laboratory Control of Infectious Diseases, Korea Centers for Diseases Control and Prevention	Jeong-Min Kim et al
**EPI_ISL_471455**	Division of Viral Diseases, Center for Laboratory Control of Infectious Diseases, Korea Centers for Diseases Control and Prevention	Division of Viral Diseases, Center for Laboratory Control of Infectious Diseases, Korea Centers for Diseases Control and Prevention	Jeong-Min Kim et al
**EPI_ISL_481370**	Division of Viral Diseases, Center for Laboratory Control of Infectious Diseases, Korea Centers for Diseases Control and Prevention	Division of Viral Diseases, Center for Laboratory Control of Infectious Diseases, Korea Centers for Diseases Control and Prevention	Jeong-Min Kim et al

**EPI_ISL_481376**	Division of Viral Diseases, Center for Laboratory Control of Infectious Diseases, Korea Centers for Diseases Control and Prevention	Division of Viral Diseases, Center for Laboratory Control of Infectious Diseases, Korea Centers for Diseases Control and Prevention	Jeong-Min Kim et al
**EPI_ISL_481377**	Division of Viral Diseases, Center for Laboratory Control of Infectious Diseases, Korea Centers for Diseases Control and Prevention	Division of Viral Diseases, Center for Laboratory Control of Infectious Diseases, Korea Centers for Diseases Control and Prevention	Jeong-Min Kim et al
**EPI_ISL_481378**	Division of Viral Diseases, Center for Laboratory Control of Infectious Diseases, Korea Centers for Diseases Control and Prevention	Division of Viral Diseases, Center for Laboratory Control of Infectious Diseases, Korea Centers for Diseases Control and Prevention	Jeong-Min Kim et al
**EPI_ISL_429853**	Centers for Disease Control and Prevention of Lishui	Department of Inspection，Centers for Disease Control and Prevention of Lishui	Wang Xiaoguang et al
**EPI_ISL_419572**	Laboratoire National de Santé, Microbiology, Virology	Laboratoire National de Santé, Microbiology, Epidemiology and Microbial Genomics	Anke Wienecke-Baldacchino et al
**EPI_ISL_412972**	Instituto Nacional de Enfermedades Respiratorias	Instituto de Diagnostico y Referencia Epidemiologicos (INDRE)	Ramirez-Gonzalez Ernesto et al
**EPI_ISL_424670**	Laboratorio Estatal de Salud Publica del Estado de Queretaro	Instituto de Diagnóstico y Referencia Epidemiologicos	Gisela Barrera Badillo et al
**EPI_ISL_421248**	Jiangxi Province Center for Disease Control and Prevention	Jiangxi Province Center for Disease Control and Prevention	JianXiong Li et al
**EPI_ISL_421245**	Jiangxi Province Center for Disease Control and Prevention	Jiangxi Province Center for Disease Control and Prevention	JianXiong Li et al
**EPI_ISL_421246**	Jiangxi Province Center for Disease Control and Prevention	Jiangxi Province Center for Disease Control and Prevention	JianXiong Li et al
**EPI_ISL_410301**	National Influenza Centre, National Public Health Laboratory, Kathmandu, Nepal	The University of Hong Kong	Ranjit Sah et al
**EPI_ISL_461101**	Dutch COVID-19 response team	Erasmus Medical Center	Bas Oude Munnink et al
**EPI_ISL_414535**	Dutch COVID-19 response team	Erasmus Medical Center	David Nieuwenhuijse et al
**EPI_ISL_414548**	Dutch COVID-19 response team	Erasmus Medical Center	David Nieuwenhuijse et al
**EPI_ISL_413490**	Auckland Hospital	Institute of Environmental Science and Research (ESR)	Matt Storey et al
**EPI_ISL_456190**	Waikato Hospital	Institute of Environmental Science and Research (ESR)	Matt Storey et al
**EPI_ISL_413550**	Centre for Human and Zoonotic Virology (CHAZVY), College of Medicine University of Lagos/Lagos University Teaching Hospital (LUTH), part of the Laboratory Network of the Nigeria Centre for Disease Control (NCDC)	African Centre of Excellence for Genomics of Infectious Diseases (ACEGID), Redeemer's University, Ede, Osun State, Nigeria	Oluniyi P.E. et al
**EPI_ISL_421252**	Jiangxi Province Center for Disease Control and Prevention	Jiangxi Province Center for Disease Control and Prevention	JianXiong Li et al
**EPI_ISL_418003**	H Braga	Instituto Nacional de Saude (INSA)	Guiomar et al et al
**EPI_ISL_418014**	CHULC - H Curry Cabral	Instituto Nacional de Saude (INSA)	Guiomar et al et al
**EPI_ISL_454004**	unknown	Instituto Nacional de Saude (INSA)	Borges et al et al
**EPI_ISL_413221**	West of Scotland Specialist Virology Centre, NHSGGC	MRC-University of Glasgow Centre for Virus Research	Emma Thomson et al
**EPI_ISL_414937**	Shandong Provincial Center for Disease Control and Prevention	Beijing Institute of Microbiology and Epidemiology	Xiao-Lin Jiang et al
**EPI_ISL_468726**	unknown	Department of Microbiology	Peng et al
**EPI_ISL_416316**	Shanghai Public Health Clinical Center, Shanghai Medical College, Fudan University	National Research Center for Translational Medicine (Shanghai), Ruijin Hospital affiliated to Shanghai Jiao Tong University School of Medicine & Shanghai Public Health Clinical Center	Shengyue Wang et al
**EPI_ISL_416318**	Shanghai Public Health Clinical Center, Shanghai Medical College, Fudan University	National Research Center for Translational Medicine (Shanghai), Ruijin Hospital affiliated to Shanghai Jiao Tong University School of Medicine & Shanghai Public Health Clinical Center	Shengyue Wang et al
**EPI_ISL_416319**	Shanghai Public Health Clinical Center, Shanghai Medical College, Fudan University	National Research Center for Translational Medicine (Shanghai), Ruijin Hospital affiliated to Shanghai Jiao Tong University School of	Shengyue Wang et al

		Medicine & Shanghai Public Health Clinical Center	
**EPI_ISL_416320**	Shanghai Public Health Clinical Center, Shanghai Medical College, Fudan University	National Research Center for Translational Medicine (Shanghai), Ruijin Hospital affiliated to Shanghai Jiao Tong University School of Medicine & Shanghai Public Health Clinical Center	Shengyue Wang et al
**EPI_ISL_416322**	Shanghai Public Health Clinical Center, Shanghai Medical College, Fudan University	National Research Center for Translational Medicine (Shanghai), Ruijin Hospital affiliated to Shanghai Jiao Tong University School of Medicine & Shanghai Public Health Clinical Center	Shengyue Wang et al
**EPI_ISL_416323**	Shanghai Public Health Clinical Center, Shanghai Medical College, Fudan University	National Research Center for Translational Medicine (Shanghai), Ruijin Hospital affiliated to Shanghai Jiao Tong University School of Medicine & Shanghai Public Health Clinical Center	Shengyue Wang et al
**EPI_ISL_416325**	Shanghai Public Health Clinical Center, Shanghai Medical College, Fudan University	National Research Center for Translational Medicine (Shanghai), Ruijin Hospital affiliated to Shanghai Jiao Tong University School of Medicine & Shanghai Public Health Clinical Center	Shengyue Wang et al
**EPI_ISL_416326**	Shanghai Public Health Clinical Center, Shanghai Medical College, Fudan University	National Research Center for Translational Medicine (Shanghai), Ruijin Hospital affiliated to Shanghai Jiao Tong University School of Medicine & Shanghai Public Health Clinical Center	Shengyue Wang et al
**EPI_ISL_416327**	Shanghai Public Health Clinical Center, Shanghai Medical College, Fudan University	National Research Center for Translational Medicine (Shanghai), Ruijin Hospital affiliated to Shanghai Jiao Tong University School of Medicine & Shanghai Public Health Clinical Center	Shengyue Wang et al
**EPI_ISL_416328**	Shanghai Public Health Clinical Center, Shanghai Medical College, Fudan University	National Research Center for Translational Medicine (Shanghai), Ruijin Hospital affiliated to Shanghai Jiao Tong University School of Medicine & Shanghai Public Health Clinical Center	Shengyue Wang et al
**EPI_ISL_416329**	Shanghai Public Health Clinical Center, Shanghai Medical College, Fudan University	National Research Center for Translational Medicine (Shanghai), Ruijin Hospital affiliated to Shanghai Jiao Tong University School of Medicine & Shanghai Public Health Clinical Center	Shengyue Wang et al
**EPI_ISL_416330**	Shanghai Public Health Clinical Center, Shanghai Medical College, Fudan University	National Research Center for Translational Medicine (Shanghai), Ruijin Hospital affiliated to Shanghai Jiao Tong University School of Medicine & Shanghai Public Health Clinical Center	Shengyue Wang et al
**EPI_ISL_416331**	Shanghai Public Health Clinical Center, Shanghai Medical College, Fudan University	National Research Center for Translational Medicine (Shanghai), Ruijin Hospital affiliated to Shanghai Jiao Tong University School of Medicine & Shanghai Public Health Clinical Center	Shengyue Wang et al
**EPI_ISL_416336**	Shanghai Public Health Clinical Center, Shanghai Medical College, Fudan University	National Research Center for Translational Medicine (Shanghai), Ruijin Hospital affiliated to Shanghai Jiao Tong University School of Medicine & Shanghai Public Health Clinical Center	Shengyue Wang et al
**EPI_ISL_416337**	Shanghai Public Health Clinical Center, Shanghai Medical College, Fudan University	National Research Center for Translational Medicine (Shanghai), Ruijin Hospital affiliated to Shanghai Jiao Tong University School of Medicine & Shanghai Public Health Clinical Center	Shengyue Wang et al
**EPI_ISL_416340**	Shanghai Public Health Clinical Center, Shanghai Medical College, Fudan University	National Research Center for Translational Medicine (Shanghai), Ruijin Hospital affiliated to Shanghai Jiao Tong University School of Medicine & Shanghai Public Health Clinical Center	Shengyue Wang et al
**EPI_ISL_416345**	Shanghai Public Health Clinical Center, Shanghai Medical College, Fudan University	National Research Center for Translational Medicine (Shanghai), Ruijin Hospital affiliated to Shanghai Jiao Tong University School of Medicine & Shanghai Public Health Clinical Center	Shengyue Wang et al
**EPI_ISL_416348**	Shanghai Public Health Clinical Center, Shanghai Medical College, Fudan University	National Research Center for Translational Medicine (Shanghai), Ruijin Hospital affiliated to Shanghai Jiao Tong University School of Medicine & Shanghai Public Health Clinical Center	Shengyue Wang et al

**EPI_ISL_416349**	Shanghai Public Health Clinical Center, Shanghai Medical College, Fudan University	National Research Center for Translational Medicine (Shanghai), Ruijin Hospital affiliated to Shanghai Jiao Tong University School of Medicine & Shanghai Public Health Clinical Center	Shengyue Wang et al
**EPI_ISL_416352**	Shanghai Public Health Clinical Center, Shanghai Medical College, Fudan University	National Research Center for Translational Medicine (Shanghai), Ruijin Hospital affiliated to Shanghai Jiao Tong University School of Medicine & Shanghai Public Health Clinical Center	Shengyue Wang et al
**EPI_ISL_416354**	Shanghai Public Health Clinical Center, Shanghai Medical College, Fudan University	National Research Center for Translational Medicine (Shanghai), Ruijin Hospital affiliated to Shanghai Jiao Tong University School of Medicine & Shanghai Public Health Clinical Center	Shengyue Wang et al
**EPI_ISL_416357**	Shanghai Public Health Clinical Center, Shanghai Medical College, Fudan University	National Research Center for Translational Medicine (Shanghai), Ruijin Hospital affiliated to Shanghai Jiao Tong University School of Medicine & Shanghai Public Health Clinical Center	Shengyue Wang et al
**EPI_ISL_416362**	Shanghai Public Health Clinical Center, Shanghai Medical College, Fudan University	National Research Center for Translational Medicine (Shanghai), Ruijin Hospital affiliated to Shanghai Jiao Tong University School of Medicine & Shanghai Public Health Clinical Center	Shengyue Wang et al
**EPI_ISL_416365**	Shanghai Public Health Clinical Center, Shanghai Medical College, Fudan University	National Research Center for Translational Medicine (Shanghai), Ruijin Hospital affiliated to Shanghai Jiao Tong University School of Medicine & Shanghai Public Health Clinical Center	Shengyue Wang et al
**EPI_ISL_416366**	Shanghai Public Health Clinical Center, Shanghai Medical College, Fudan University	National Research Center for Translational Medicine (Shanghai), Ruijin Hospital affiliated to Shanghai Jiao Tong University School of Medicine & Shanghai Public Health Clinical Center	Shengyue Wang et al
**EPI_ISL_416367**	Shanghai Public Health Clinical Center, Shanghai Medical College, Fudan University	National Research Center for Translational Medicine (Shanghai), Ruijin Hospital affiliated to Shanghai Jiao Tong University School of Medicine & Shanghai Public Health Clinical Center	Shengyue Wang et al
**EPI_ISL_416368**	Shanghai Public Health Clinical Center, Shanghai Medical College, Fudan University	National Research Center for Translational Medicine (Shanghai), Ruijin Hospital affiliated to Shanghai Jiao Tong University School of Medicine & Shanghai Public Health Clinical Center	Shengyue Wang et al
**EPI_ISL_416369**	Shanghai Public Health Clinical Center, Shanghai Medical College, Fudan University	National Research Center for Translational Medicine (Shanghai), Ruijin Hospital affiliated to Shanghai Jiao Tong University School of Medicine & Shanghai Public Health Clinical Center	Shengyue Wang et al
**EPI_ISL_416370**	Shanghai Public Health Clinical Center, Shanghai Medical College, Fudan University	National Research Center for Translational Medicine (Shanghai), Ruijin Hospital affiliated to Shanghai Jiao Tong University School of Medicine & Shanghai Public Health Clinical Center	Shengyue Wang et al
**EPI_ISL_416372**	Shanghai Public Health Clinical Center, Shanghai Medical College, Fudan University	National Research Center for Translational Medicine (Shanghai), Ruijin Hospital affiliated to Shanghai Jiao Tong University School of Medicine & Shanghai Public Health Clinical Center	Shengyue Wang et al
**EPI_ISL_416373**	Shanghai Public Health Clinical Center, Shanghai Medical College, Fudan University	National Research Center for Translational Medicine (Shanghai), Ruijin Hospital affiliated to Shanghai Jiao Tong University School of Medicine & Shanghai Public Health Clinical Center	Shengyue Wang et al
**EPI_ISL_416376**	Shanghai Public Health Clinical Center, Shanghai Medical College, Fudan University	National Research Center for Translational Medicine (Shanghai), Ruijin Hospital affiliated to Shanghai Jiao Tong University School of Medicine & Shanghai Public Health Clinical Center	Shengyue Wang et al
**EPI_ISL_416378**	Shanghai Public Health Clinical Center, Shanghai Medical College, Fudan University	National Research Center for Translational Medicine (Shanghai), Ruijin Hospital affiliated to Shanghai Jiao Tong University School of Medicine & Shanghai Public Health Clinical Center	Shengyue Wang et al

**EPI_ISL_416379**	Shanghai Public Health Clinical Center, Shanghai Medical College, Fudan University	National Research Center for Translational Medicine (Shanghai), Ruijin Hospital affiliated to Shanghai Jiao Tong University School of Medicine & Shanghai Public Health Clinical Center	Shengyue Wang et al
**EPI_ISL_416380**	Shanghai Public Health Clinical Center, Shanghai Medical College, Fudan University	National Research Center for Translational Medicine (Shanghai), Ruijin Hospital affiliated to Shanghai Jiao Tong University School of Medicine & Shanghai Public Health Clinical Center	Shengyue Wang et al
**EPI_ISL_416381**	Shanghai Public Health Clinical Center, Shanghai Medical College, Fudan University	National Research Center for Translational Medicine (Shanghai), Ruijin Hospital affiliated to Shanghai Jiao Tong University School of Medicine & Shanghai Public Health Clinical Center	Shengyue Wang et al
**EPI_ISL_416383**	Shanghai Public Health Clinical Center, Shanghai Medical College, Fudan University	National Research Center for Translational Medicine (Shanghai), Ruijin Hospital affiliated to Shanghai Jiao Tong University School of Medicine & Shanghai Public Health Clinical Center	Shengyue Wang et al
**EPI_ISL_416384**	Shanghai Public Health Clinical Center, Shanghai Medical College, Fudan University	National Research Center for Translational Medicine (Shanghai), Ruijin Hospital affiliated to Shanghai Jiao Tong University School of Medicine & Shanghai Public Health Clinical Center	Shengyue Wang et al
**EPI_ISL_416385**	Shanghai Public Health Clinical Center, Shanghai Medical College, Fudan University	National Research Center for Translational Medicine (Shanghai), Ruijin Hospital affiliated to Shanghai Jiao Tong University School of Medicine & Shanghai Public Health Clinical Center	Shengyue Wang et al
**EPI_ISL_416389**	Shanghai Public Health Clinical Center, Shanghai Medical College, Fudan University	National Research Center for Translational Medicine (Shanghai), Ruijin Hospital affiliated to Shanghai Jiao Tong University School of Medicine & Shanghai Public Health Clinical Center	Shengyue Wang et al
**EPI_ISL_416390**	Shanghai Public Health Clinical Center, Shanghai Medical College, Fudan University	National Research Center for Translational Medicine (Shanghai), Ruijin Hospital affiliated to Shanghai Jiao Tong University School of Medicine & Shanghai Public Health Clinical Center	Shengyue Wang et al
**EPI_ISL_416392**	Shanghai Public Health Clinical Center, Shanghai Medical College, Fudan University	National Research Center for Translational Medicine (Shanghai), Ruijin Hospital affiliated to Shanghai Jiao Tong University School of Medicine & Shanghai Public Health Clinical Center	Shengyue Wang et al
**EPI_ISL_416393**	Shanghai Public Health Clinical Center, Shanghai Medical College, Fudan University	National Research Center for Translational Medicine (Shanghai), Ruijin Hospital affiliated to Shanghai Jiao Tong University School of Medicine & Shanghai Public Health Clinical Center	Shengyue Wang et al
**EPI_ISL_416394**	Shanghai Public Health Clinical Center, Shanghai Medical College, Fudan University	National Research Center for Translational Medicine (Shanghai), Ruijin Hospital affiliated to Shanghai Jiao Tong University School of Medicine & Shanghai Public Health Clinical Center	Shengyue Wang et al
**EPI_ISL_416396**	Shanghai Public Health Clinical Center, Shanghai Medical College, Fudan University	National Research Center for Translational Medicine (Shanghai), Ruijin Hospital affiliated to Shanghai Jiao Tong University School of Medicine & Shanghai Public Health Clinical Center	Shengyue Wang et al
**EPI_ISL_416398**	Shanghai Public Health Clinical Center, Shanghai Medical College, Fudan University	National Research Center for Translational Medicine (Shanghai), Ruijin Hospital affiliated to Shanghai Jiao Tong University School of Medicine & Shanghai Public Health Clinical Center	Shengyue Wang et al
**EPI_ISL_416399**	Shanghai Public Health Clinical Center, Shanghai Medical College, Fudan University	National Research Center for Translational Medicine (Shanghai), Ruijin Hospital affiliated to Shanghai Jiao Tong University School of Medicine & Shanghai Public Health Clinical Center	Shengyue Wang et al
**EPI_ISL_416401**	Shanghai Public Health Clinical Center, Shanghai Medical College, Fudan University	National Research Center for Translational Medicine (Shanghai), Ruijin Hospital affiliated to Shanghai Jiao Tong University School of Medicine & Shanghai Public Health Clinical Center	Shengyue Wang et al

**EPI_ISL_416402**	Shanghai Public Health Clinical Center, Shanghai Medical College, Fudan University	National Research Center for Translational Medicine (Shanghai), Ruijin Hospital affiliated to Shanghai Jiao Tong University School of Medicine & Shanghai Public Health Clinical Center	Shengyue Wang et al
**EPI_ISL_416403**	Shanghai Public Health Clinical Center, Shanghai Medical College, Fudan University	National Research Center for Translational Medicine (Shanghai), Ruijin Hospital affiliated to Shanghai Jiao Tong University School of Medicine & Shanghai Public Health Clinical Center	Shengyue Wang et al
**EPI_ISL_416404**	Shanghai Public Health Clinical Center, Shanghai Medical College, Fudan University	National Research Center for Translational Medicine (Shanghai), Ruijin Hospital affiliated to Shanghai Jiao Tong University School of Medicine & Shanghai Public Health Clinical Center	Shengyue Wang et al
**EPI_ISL_416406**	Shanghai Public Health Clinical Center, Shanghai Medical College, Fudan University	National Research Center for Translational Medicine (Shanghai), Ruijin Hospital affiliated to Shanghai Jiao Tong University School of Medicine & Shanghai Public Health Clinical Center	Shengyue Wang et al
**EPI_ISL_416407**	Shanghai Public Health Clinical Center, Shanghai Medical College, Fudan University	National Research Center for Translational Medicine (Shanghai), Ruijin Hospital affiliated to Shanghai Jiao Tong University School of Medicine & Shanghai Public Health Clinical Center	Shengyue Wang et al
**EPI_ISL_416408**	Shanghai Public Health Clinical Center, Shanghai Medical College, Fudan University	National Research Center for Translational Medicine (Shanghai), Ruijin Hospital affiliated to Shanghai Jiao Tong University School of Medicine & Shanghai Public Health Clinical Center	Shengyue Wang et al
**EPI_ISL_416409**	Shanghai Public Health Clinical Center, Shanghai Medical College, Fudan University	National Research Center for Translational Medicine (Shanghai), Ruijin Hospital affiliated to Shanghai Jiao Tong University School of Medicine & Shanghai Public Health Clinical Center	Shengyue Wang et al
**EPI_ISL_421254**	Jiangxi Province Center for Disease Control and Prevention	Jiangxi Province Center for Disease Control and Prevention	JianXiong Li et al
**EPI_ISL_463895**	Shaoxing Center for Disease Control and Prevention	Department of Pathology and Laboratory Medicine, University of California Los Angeles	Jinkun Chen et al
**EPI_ISL_463902**	Shaoxing Center for Disease Control and Prevention	Department of Pathology and Laboratory Medicine, University of California Los Angeles	Jinkun Chen et al
**EPI_ISL_406030**	The University of Hong Kong - Shenzhen Hospital	Li Ka Shing Faculty of Medicine, The University of Hong Kong	Chan et al
**EPI_ISL_405839**	The University of Hong Kong - Shenzhen Hospital	Li Ka Shing Faculty of Medicine, The University of Hong Kong	Chan et al
**EPI_ISL_406593**	Shenzhen Key Laboratory of Pathogen and Immunity, National Clinical Research Center for Infectious Disease, Shenzhen Third People's Hospital	Shenzhen Key Laboratory of Pathogen and Immunity, National Clinical Research Center for Infectious Disease, Shenzhen Third People's Hospital	Yang Yang et al
**EPI_ISL_408484**	National Institute for Viral Disease Control and Prevention, China CDC	National Institute for Viral Disease Control & Prevention, CCDC	Wenjie Tan et al
**EPI_ISL_451318**	West China Hospital of Sichuan University	State Key Laboratory of Biotherapy of Sichuan University	Baowen Du et al
**EPI_ISL_451368**	West China Hospital of Sichuan University	State Key Laboratory of Biotherapy of Sichuan University	Baowen Du et al
**EPI_ISL_451388**	West China Hospital of Sichuan University	State Key Laboratory of Biotherapy of Sichuan University	Baowen Du et al
**EPI_ISL_406973**	Singapore General Hospital	National Public Health Laboratory	Mak et al
**EPI_ISL_462306**	National Public Health Laboratory, National Centre for Infectious Diseases	National Public Health Laboratory, National Centre for Infectious Diseases	Mak TM et al
**EPI_ISL_462373**	National Public Health Laboratory, National Centre for Infectious Diseases	National Public Health Laboratory, National Centre for Infectious Diseases	Mak TM et al
**EPI_ISL_482680**	Singapore General Hospital	Department of Microbiology	Nurdyana Abdul Rahman et al
**EPI_ISL_418994**	National Public Health Laboratory, National Centre for Infectious Diseases	National Public Health Laboratory, National Centre for Infectious Diseases	Mak TM et al
**EPI_ISL_428823**	National Public Health Laboratory, National Centre for Infectious Diseases	National Public Health Laboratory, National Centre for Infectious Diseases	Mak TM et al
**EPI_ISL_476811**	Department of Laboratory Medicine Tan Tock Seng Hospital	Department of Laboratory Medicine Tan Tock Seng Hospital	Chen YYC et al
**EPI_ISL_410537**	Singapore General Hospital, Molecular Laboratory, Division of Pathology	Programme in Emerging Infectious Diseases, Duke-NUS Medical School	Danielle E Anderson et al
**EPI_ISL_485001**	University of Ulsan College of Medicine and Asan Medical Center	University of Ulsan College of Medicine and Asan Medical Center	Kuenyoul Park et al
**EPI_ISL_407193**	Korea Centers for Disease Control & Prevention (KCDC) Center for	Korea Centers for Disease Control & Prevention (KCDC) Center for Laboratory	Jeong-Min Kim et al

	Laboratory Control of Infectious Diseases Division of Viral Diseases	Control of Infectious Diseases Division of Viral Diseases	
**EPI_ISL_412869**	Division of Viral Diseases, Center for Laboratory Control of Infectious Diseases, Korea Centers for Diseases Control and Prevention	Division of Viral Diseases, Center for Laboratory Control of Infectious Diseases, Korea Centers for Diseases Control and Prevention	Jeong-Min Kim et al
**EPI_ISL_412872**	Division of Viral Diseases, Center for Laboratory Control of Infectious Diseases, Korea Centers for Diseases Control and Prevention	Division of Viral Diseases, Center for Laboratory Control of Infectious Diseases, Korea Centers for Diseases Control and Prevention	Jeong-Min Kim et al
**EPI_ISL_412873**	Division of Viral Diseases, Center for Laboratory Control of Infectious Diseases, Korea Centers for Diseases Control and Prevention	Division of Viral Diseases, Center for Laboratory Control of Infectious Diseases, Korea Centers for Diseases Control and Prevention	Jeong-Min Kim et al
**EPI_ISL_413514**	Department of Microbiology, Institute for Viral Diseases, College of Medicine, Korea University	Department of Microbiology, Institute for Viral Diseases, College of Medicine, Korea University	Changmin Kang et al
**EPI_ISL_413515**	Division of Infectious Diseases, Department of Internal Medicine, Korea University College of Medicine	Department of Microbiology, Institute for Viral Diseases, College of Medicine, Korea University	Changmin Kang et al
**EPI_ISL_413516**	Department of Microbiology, Institute for Viral Diseases, College of Medicine, Korea University	Department of Microbiology, Institute for Viral Diseases, College of Medicine, Korea University	Changmin Kang et al
**EPI_ISL_452777**	Hospital Universitario Araba. Vitoria-Gasteiz,	SeqCOVID-SPAIN consortium/IBV(CSIC)	Silvia Hernáez Crespo et al
**EPI_ISL_452775**	Hospital Universitario Araba. Vitoria-Gasteiz,	SeqCOVID-SPAIN consortium/IBV(CSIC)	Silvia Hernáez Crespo et al
**EPI_ISL_452731**	Hospital Universitario Araba. Vitoria-Gasteiz,	SeqCOVID-SPAIN consortium/IBV(CSIC)	Silvia Hernáez Crespo et al
**EPI_ISL_452729**	Hospital Universitario Araba. Vitoria-Gasteiz,	SeqCOVID-SPAIN consortium/IBV(CSIC)	Silvia Hernáez Crespo et al
**EPI_ISL_452726**	Hospital Universitario Araba. Vitoria-Gasteiz,	SeqCOVID-SPAIN consortium/IBV(CSIC)	Silvia Hernáez Crespo et al
**EPI_ISL_452717**	Hospital Universitario Araba. Vitoria-Gasteiz,	SeqCOVID-SPAIN consortium/IBV(CSIC)	Silvia Hernáez Crespo et al
**EPI_ISL_452543**	Clínica Universidad de Navarra. Servicio de Enfermedades Infecciosas y Microbiología clínica	SeqCOVID-SPAIN consortium/IBV(CSIC)	Mirian Fernández-Alonso et al
**EPI_ISL_452471**	Hospital Universitario Puerta del Mar de Cádiz - INIBICA	SeqCOVID-SPAIN consortium/IBV(CSIC)	Salud Rodríguez-Pallares et al
**EPI_ISL_481013**	Servicio de Microbiología. Hospital Universitario Donostia. OSI Donostialdea. Área de Enfermedades Infecciosas, Grupo de Infección Respiratoria y Resistencia Antimicrobiana. Instituto de Investigación Sanitaria Biodonostia	SeqCOVID-SPAIN consortium/IBV(CSIC)	Gustavo Cilla et al
**EPI_ISL_481052**	Hospital General Universitario Gregorio Marañón	SeqCOVID-SPAIN consortium/IBV(CSIC)	Laura Pérez-Lago et al
**EPI_ISL_420118**	Servicio de Microbiología. Consorcio Hospital General Universitario de Valencia	Sequencing and Bioinformatics Service and Molecular Epidemiology Research Group. FISABIO-Public Health	Inma Galán Vendrell et al
**EPI_ISL_420121**	Servicio de Microbiología. Consorcio Hospital General Universitario de Valencia	Sequencing and Bioinformatics Service and Molecular Epidemiology Research Group. FISABIO-Public Health	Vicente Soriano Chirona et al
**EPI_ISL_425182**	Servicio de Microbiología. Consorcio Hospital General Universitario de Valencia	Sequencing and Bioinformatics Service and Molecular Epidemiology Research Group. FISABIO-Public Health	Lidia Ruiz Roldan et al
**EPI_ISL_468784**	Servicio de Microbiología, Hospital Miguel Servet, Zaragoza	SeqCOVID-SPAIN consortium/IBV(CSIC)	Antonio Rezusta López et al
**EPI_ISL_411951**	unknown	Unit for Laboratory Development and Technology Transfer, Public Health Agency of Sweden	Bengner et al
**EPI_ISL_475547**	Karolinska Universitetslaboratoriet	The Public Health Agency of Sweden	Oskar Karlsson Lindsjo et al
**EPI_ISL_454486**	Karolinska Universitetslaboratoriet	The Public Health Agency of Sweden	Anna-Malin Linde et al
**EPI_ISL_454492**	Karolinska Universitetslaboratoriet	The Public Health Agency of Sweden	Anna-Malin Linde et al
**EPI_ISL_430862**	The Public Health Agency of Sweden	The Public Health Agency of Sweden	Oskar Karlsson Lindsjo et al
**EPI_ISL_466942**	Viollier AG	Department of Biosystems Science and Engineering, ETH Zürich	Christian Beisel et al
**EPI_ISL_466957**	Viollier AG	Department of Biosystems Science and Engineering, ETH Zürich	Christian Beisel et al

**EPI_ISL_418436**	University Hospital Basel, Clinical Virology	University Hospital Basel, Clinical Virology	Hirsch et al
**EPI_ISL_411915**	Laboratory Medicine	Department of Laboratory Medicine, Lin-Kou Chang Gung Memorial Hospital, Taoyuan, Taiwan.	Kuo-Chien Tsao et al
**EPI_ISL_417521**	Laboratory Medicine	Department of Laboratory Medicine, Lin-Kou Chang Gung Memorial Hospital, Taoyuan, Taiwan	Kuo-Chien Tsao et al
**EPI_ISL_408489**	Department of Laboratory Medicine, National Taiwan University Hospital	Microbial Genomics Core Lab, National Taiwan University Centers of Genomic and Precision Medicine	Shiou-Hwei Yeh et al
**EPI_ISL_410218**	Department of Laboratory Medicine, National Taiwan University Hospital	Microbial Genomics Core Lab, National Taiwan University Centers of Genomic and Precision Medicine	Shiou-Hwei Yeh et al
**EPI_ISL_422407**	Department of Laboratory Medicine, National Taiwan University Hospital	Microbial Genomics Core Lab, National Taiwan University Centers of Genomic and Precision Medicine	Shiou-Hwei Yeh et al
**EPI_ISL_403962**	Bamrasnaradura Hospital	1. Department of Medical Sciences, Ministry of Public Health, Thailand 2. Thai Red Cross Emerging Infectious Diseases - Health Science Centre 3. Department of Disease Control, Ministry of Public Health, Thailand	Pilailuk et al
**EPI_ISL_403963**	Bamrasnaradura Hospital	1. Department of Medical Sciences, Ministry of Public Health, Thailand 2. Thai Red Cross Emerging Infectious Diseases - Health Science Centre 3. Department of Disease Control, Ministry of Public Health, Thailand	Pilailuk et al
**EPI_ISL_437623**	unknown	Faculty of Medicine	Rodpan et al
**EPI_ISL_437615**	unknown	Faculty of Medicine	Rodpan et al
**EPI_ISL_412983**	Tianmen Center for Disease Control and Prevention	Hubei Provincial Center for Disease Control and Prevention	Bin Fang et al
**EPI_ISL_406223**	Arizona Department of Health Services	Pathogen Discovery, Respiratory Viruses Branch, Division of Viral Diseases, Centers for Disease Control and Prevention	Ying Tao et al
**EPI_ISL_435585**	Santa Clara County Public Health Department	Chiu Laboratory, University of California, San Francisco	Xianding Deng et al
**EPI_ISL_435635**	Santa Clara County Public Health Department	Chiu Laboratory, University of California, San Francisco	Xianding Deng et al
**EPI_ISL_406034**	California Department of Public Health	Pathogen Discovery, Respiratory Viruses Branch, Division of Viral Diseases, Centers for Disease Control and Prevention	Anna Uehara et al
**EPI_ISL_406036**	California Department of Public Health	Pathogen Discovery, Respiratory Viruses Branch, Division of Viral Diseases, Centers for Disease Control and Prevention	Anna Uehara et al
**EPI_ISL_408008**	California Department of Health	Pathogen Discovery, Respiratory Viruses Branch, Division of Viral Diseases, Centers for Disease Control and Prevention	Krista Queen et al
**EPI_ISL_408010**	California Department of Health	Pathogen Discovery, Respiratory Viruses Branch, Division of Viral Diseases, Centers for Disease Control and Prevention	Ying Tao et al
**EPI_ISL_411954**	California Department of Public Health	Pathogen Discovery, Respiratory Viruses Branch, Division of Viral Diseases, Centers for Disease Control and Prevention	Krista Queen et al
**EPI_ISL_411955**	California Department of Public Health	Pathogen Discovery, Respiratory Viruses Branch, Division of Viral Diseases, Centers for Disease Control and Prevention	Krista Queen et al
**EPI_ISL_413606**	unknown	Pathogen Discovery, Respiratory Viruses Branch, Division of Viral Diseases, Centers for Disease Control and Prevention	Anna Uehara et al
**EPI_ISL_414479**	unknown	Pathogen Discovery, Respiratory Viruses Branch, Division of Viral Diseases, Centers for Disease Control and Prevention	Ying Tao et al
**EPI_ISL_414484**	unknown	Pathogen Discovery, Respiratory Viruses Branch, Division of Viral Diseases, Centers for Disease Control and Prevention	Krista Queen et al
**EPI_ISL_413609**	unknown	Pathogen Discovery, Respiratory Viruses Branch, Division of Viral Diseases, Centers for Disease Control and Prevention	Anna Uehara et al
**EPI_ISL_413613**	unknown	Pathogen Discovery, Respiratory Viruses Branch, Division of Viral Diseases, Centers for Disease Control and Prevention	Ying Tao et al
**EPI_ISL_447840**	DC Public Health Lab/ Dept. of Forensic Sciences	Pathogen Discovery, Respiratory Viruses Branch, Division of Viral Diseases, Centers for Disease Control and Prevention	Krista Queen et al

**EPI_ISL_409067**	Massachusetts Department of Public Health	Pathogen Discovery, Respiratory Viruses Branch, Division of Viral Diseases, Centers for Disease Control and Prevention	Clinton R. Paden et al
**EPI_ISL_424914**	MA State Public Health Laboratory	Pathogen Discovery, Respiratory Viruses Branch, Division of Viral Diseases, Centers for Disease Control and Prevention	Ying Tao et al
**EPI_ISL_454427**	Maryland Department of Health Laboratories Administration	Maryland Department of Health Laboratories Administration	MDH Laboratories Administration et al
**EPI_ISL_450114**	MSHS Clinical Microbiology Laboratories	MSHS Pathogen Surveillance Program	Ana S. Gonzalez-Reiche et al
**EPI_ISL_436062**	NYC Department of Health and Mental Hygiene	Pathogen Discovery, Respiratory Viruses Branch, Division of Viral Diseases, Centers for Disease Control and Prevention	Ying Tao et al
**EPI_ISL_426290**	Wadsworth Center, New York State Department of Health	Wadsworth Center, New York State Department.of Health	Kirsten St. George et al
**EPI_ISL_420793**	NYC Department of Health and Mental Hygiene	Pathogen Discovery, Respiratory Viruses Branch, Division of Viral Diseases, Centers for Disease Control and Prevention	Krista Queen et al
**EPI_ISL_411956**	Texas Department of State Health Services	Pathogen Discovery, Respiratory Viruses Branch, Division of Viral Diseases, Centers for Disease Control and Prevention	Krista Queen et al
**EPI_ISL_430283**	Washington State Department of Health	Seattle Flu Study	Chu et al et al
**EPI_ISL_417095**	Washington State Department of Health	Seattle Flu Study	Chu etl al et al
**EPI_ISL_417148**	Washington State Department of Health	Seattle Flu Study	Chu etl al et al
**EPI_ISL_415607**	UW Virology Lab	UW Virology Lab	Pavitra Roychoudhury et al
**EPI_ISL_404895**	Providence Regional Medical Center	Division of Viral Diseases, Centers for Disease Control and Prevention	Queen et al
**EPI_ISL_408670**	Wisconsin Department of Health Services	Pathogen Discovery, Respiratory Viruses Branch, Division of Viral Diseases, Centers for Disease Control and Prevention	Jing Zhang et al
**EPI_ISL_435312**	National Hospital of Tropical Diseases	Oxford University Clinical Research Unit, Hanoi, Vietnam	Nguyen Thi Tam et al
**EPI_ISL_455714**	National Hospital of Tropical Diseases	Oxford University Clinical Research Unit, Hanoi, Vietnam	Nguyen Thi Tam et al
**EPI_ISL_408668**	National Influenza Center - National Institute of Hygiene and Epidemiology (NIHE)	National Influenza Center - National Institute of Hygiene and Epidemiology (NIHE)	Ung Thi Hong Trang et al
**EPI_ISL_415453**	Wales Specialist Virology Centre	Public Health Wales Microbiology Cardiff	Catherine Moore et al
**EPI_ISL_455363**	Wuhan Chain Medical Labs (CMLabs)	State Key Laboratory of Biotherapy of Sichuan University	Baowen Du et al
**EPI_ISL_454905**	Wuhan Chain Medical Labs (CMLabs)	State Key Laboratory of Biotherapy of Sichuan University	Baowen Du et al
**EPI_ISL_454906**	Wuhan Chain Medical Labs (CMLabs)	State Key Laboratory of Biotherapy of Sichuan University	Baowen Du et al
**EPI_ISL_454907**	Wuhan Chain Medical Labs (CMLabs)	State Key Laboratory of Biotherapy of Sichuan University	Baowen Du et al
**EPI_ISL_454910**	Wuhan Chain Medical Labs (CMLabs)	State Key Laboratory of Biotherapy of Sichuan University	Baowen Du et al
**EPI_ISL_455365**	Wuhan Chain Medical Labs (CMLabs)	State Key Laboratory of Biotherapy of Sichuan University	Baowen Du et al
**EPI_ISL_455366**	Wuhan Chain Medical Labs (CMLabs)	State Key Laboratory of Biotherapy of Sichuan University	Baowen Du et al
**EPI_ISL_454911**	Wuhan Chain Medical Labs (CMLabs)	State Key Laboratory of Biotherapy of Sichuan University	Baowen Du et al
**EPI_ISL_454912**	Wuhan Chain Medical Labs (CMLabs)	State Key Laboratory of Biotherapy of Sichuan University	Baowen Du et al
**EPI_ISL_454914**	Wuhan Chain Medical Labs (CMLabs)	State Key Laboratory of Biotherapy of Sichuan University	Baowen Du et al
**EPI_ISL_455367**	Wuhan Chain Medical Labs (CMLabs)	State Key Laboratory of Biotherapy of Sichuan University	Baowen Du et al
**EPI_ISL_454917**	Wuhan Chain Medical Labs (CMLabs)	State Key Laboratory of Biotherapy of Sichuan University	Baowen Du et al
**EPI_ISL_454918**	Wuhan Chain Medical Labs (CMLabs)	State Key Laboratory of Biotherapy of Sichuan University	Baowen Du et al
**EPI_ISL_454919**	Wuhan Chain Medical Labs (CMLabs)	State Key Laboratory of Biotherapy of Sichuan University	Baowen Du et al
**EPI_ISL_454920**	Wuhan Chain Medical Labs (CMLabs)	State Key Laboratory of Biotherapy of Sichuan University	Baowen Du et al

**EPI_ISL_454921**	Wuhan Chain Medical Labs (CMLabs)	State Key Laboratory of Biotherapy of Sichuan University	Baowen Du et al
**EPI_ISL_454922**	Wuhan Chain Medical Labs (CMLabs)	State Key Laboratory of Biotherapy of Sichuan University	Baowen Du et al
**EPI_ISL_454923**	Wuhan Chain Medical Labs (CMLabs)	State Key Laboratory of Biotherapy of Sichuan University	Baowen Du et al
**EPI_ISL_454924**	Wuhan Chain Medical Labs (CMLabs)	State Key Laboratory of Biotherapy of Sichuan University	Baowen Du et al
**EPI_ISL_454926**	Wuhan Chain Medical Labs (CMLabs)	State Key Laboratory of Biotherapy of Sichuan University	Baowen Du et al
**EPI_ISL_454927**	Wuhan Chain Medical Labs (CMLabs)	State Key Laboratory of Biotherapy of Sichuan University	Baowen Du et al
**EPI_ISL_454929**	Wuhan Chain Medical Labs (CMLabs)	State Key Laboratory of Biotherapy of Sichuan University	Baowen Du et al
**EPI_ISL_455374**	Wuhan Chain Medical Labs (CMLabs)	State Key Laboratory of Biotherapy of Sichuan University	Baowen Du et al
**EPI_ISL_454930**	Wuhan Chain Medical Labs (CMLabs)	State Key Laboratory of Biotherapy of Sichuan University	Baowen Du et al
**EPI_ISL_454931**	Wuhan Chain Medical Labs (CMLabs)	State Key Laboratory of Biotherapy of Sichuan University	Baowen Du et al
**EPI_ISL_455375**	Wuhan Chain Medical Labs (CMLabs)	State Key Laboratory of Biotherapy of Sichuan University	Baowen Du et al
**EPI_ISL_454932**	Wuhan Chain Medical Labs (CMLabs)	State Key Laboratory of Biotherapy of Sichuan University	Baowen Du et al
**EPI_ISL_454933**	Wuhan Chain Medical Labs (CMLabs)	State Key Laboratory of Biotherapy of Sichuan University	Baowen Du et al
**EPI_ISL_454934**	Wuhan Chain Medical Labs (CMLabs)	State Key Laboratory of Biotherapy of Sichuan University	Baowen Du et al
**EPI_ISL_454935**	Wuhan Chain Medical Labs (CMLabs)	State Key Laboratory of Biotherapy of Sichuan University	Baowen Du et al
**EPI_ISL_454936**	Wuhan Chain Medical Labs (CMLabs)	State Key Laboratory of Biotherapy of Sichuan University	Baowen Du et al
**EPI_ISL_454937**	Wuhan Chain Medical Labs (CMLabs)	State Key Laboratory of Biotherapy of Sichuan University	Baowen Du et al
**EPI_ISL_454939**	Wuhan Chain Medical Labs (CMLabs)	State Key Laboratory of Biotherapy of Sichuan University	Baowen Du et al
**EPI_ISL_455376**	Wuhan Chain Medical Labs (CMLabs)	State Key Laboratory of Biotherapy of Sichuan University	Baowen Du et al
**EPI_ISL_454940**	Wuhan Chain Medical Labs (CMLabs)	State Key Laboratory of Biotherapy of Sichuan University	Baowen Du et al
**EPI_ISL_455377**	Wuhan Chain Medical Labs (CMLabs)	State Key Laboratory of Biotherapy of Sichuan University	Baowen Du et al
**EPI_ISL_454943**	Wuhan Chain Medical Labs (CMLabs)	State Key Laboratory of Biotherapy of Sichuan University	Baowen Du et al
**EPI_ISL_454947**	Wuhan Chain Medical Labs (CMLabs)	State Key Laboratory of Biotherapy of Sichuan University	Baowen Du et al
**EPI_ISL_455385**	Wuhan Chain Medical Labs (CMLabs)	State Key Laboratory of Biotherapy of Sichuan University	Baowen Du et al
**EPI_ISL_455386**	Wuhan Chain Medical Labs (CMLabs)	State Key Laboratory of Biotherapy of Sichuan University	Baowen Du et al
**EPI_ISL_454952**	Wuhan Chain Medical Labs (CMLabs)	State Key Laboratory of Biotherapy of Sichuan University	Baowen Du et al
**EPI_ISL_454955**	Wuhan Chain Medical Labs (CMLabs)	State Key Laboratory of Biotherapy of Sichuan University	Baowen Du et al
**EPI_ISL_454964**	Wuhan Chain Medical Labs (CMLabs)	State Key Laboratory of Biotherapy of Sichuan University	Baowen Du et al
**EPI_ISL_454965**	Wuhan Chain Medical Labs (CMLabs)	State Key Laboratory of Biotherapy of Sichuan University	Baowen Du et al
**EPI_ISL_454968**	Wuhan Chain Medical Labs (CMLabs)	State Key Laboratory of Biotherapy of Sichuan University	Baowen Du et al
**EPI_ISL_455391**	Wuhan Chain Medical Labs (CMLabs)	State Key Laboratory of Biotherapy of Sichuan University	Baowen Du et al
**EPI_ISL_454973**	Wuhan Chain Medical Labs (CMLabs)	State Key Laboratory of Biotherapy of Sichuan University	Baowen Du et al
**EPI_ISL_454974**	Wuhan Chain Medical Labs (CMLabs)	State Key Laboratory of Biotherapy of Sichuan University	Baowen Du et al
**EPI_ISL_454980**	Wuhan Chain Medical Labs (CMLabs)	State Key Laboratory of Biotherapy of Sichuan University	Baowen Du et al
**EPI_ISL_455401**	Wuhan Chain Medical Labs (CMLabs)	State Key Laboratory of Biotherapy of Sichuan University	Baowen Du et al
**EPI_ISL_454992**	Wuhan Chain Medical Labs (CMLabs)	State Key Laboratory of Biotherapy of Sichuan University	Baowen Du et al
**EPI_ISL_455403**	Wuhan Chain Medical Labs (CMLabs)	State Key Laboratory of Biotherapy of Sichuan University	Baowen Du et al

**EPI_ISL_454993**	Wuhan Chain Medical Labs (CMLabs)	State Key Laboratory of Biotherapy of Sichuan University	Baowen Du et al
**EPI_ISL_454995**	Wuhan Chain Medical Labs (CMLabs)	State Key Laboratory of Biotherapy of Sichuan University	Baowen Du et al
**EPI_ISL_454996**	Wuhan Chain Medical Labs (CMLabs)	State Key Laboratory of Biotherapy of Sichuan University	Baowen Du et al
**EPI_ISL_454997**	Wuhan Chain Medical Labs (CMLabs)	State Key Laboratory of Biotherapy of Sichuan University	Baowen Du et al
**EPI_ISL_454998**	Wuhan Chain Medical Labs (CMLabs)	State Key Laboratory of Biotherapy of Sichuan University	Baowen Du et al
**EPI_ISL_454999**	Wuhan Chain Medical Labs (CMLabs)	State Key Laboratory of Biotherapy of Sichuan University	Baowen Du et al
**EPI_ISL_455000**	Wuhan Chain Medical Labs (CMLabs)	State Key Laboratory of Biotherapy of Sichuan University	Baowen Du et al
**EPI_ISL_455002**	Wuhan Chain Medical Labs (CMLabs)	State Key Laboratory of Biotherapy of Sichuan University	Baowen Du et al
**EPI_ISL_455004**	Wuhan Chain Medical Labs (CMLabs)	State Key Laboratory of Biotherapy of Sichuan University	Baowen Du et al
**EPI_ISL_455005**	Wuhan Chain Medical Labs (CMLabs)	State Key Laboratory of Biotherapy of Sichuan University	Baowen Du et al
**EPI_ISL_455006**	Wuhan Chain Medical Labs (CMLabs)	State Key Laboratory of Biotherapy of Sichuan University	Baowen Du et al
**EPI_ISL_455409**	Wuhan Chain Medical Labs (CMLabs)	State Key Laboratory of Biotherapy of Sichuan University	Baowen Du et al
**EPI_ISL_455008**	Wuhan Chain Medical Labs (CMLabs)	State Key Laboratory of Biotherapy of Sichuan University	Baowen Du et al
**EPI_ISL_455410**	Wuhan Chain Medical Labs (CMLabs)	State Key Laboratory of Biotherapy of Sichuan University	Baowen Du et al
**EPI_ISL_455010**	Wuhan Chain Medical Labs (CMLabs)	State Key Laboratory of Biotherapy of Sichuan University	Baowen Du et al
**EPI_ISL_455011**	Wuhan Chain Medical Labs (CMLabs)	State Key Laboratory of Biotherapy of Sichuan University	Baowen Du et al
**EPI_ISL_455012**	Wuhan Chain Medical Labs (CMLabs)	State Key Laboratory of Biotherapy of Sichuan University	Baowen Du et al
**EPI_ISL_455013**	Wuhan Chain Medical Labs (CMLabs)	State Key Laboratory of Biotherapy of Sichuan University	Baowen Du et al
**EPI_ISL_402132**	Wuhan Jinyintan Hospital	Hubei Provincial Center for Disease Control and Prevention	Bin Fang et al
**EPI_ISL_412898**	Wuhan Jinyintan Hospital	Hubei Provincial Center for Disease Control and Prevention	Bin Fang et al
**EPI_ISL_412899**	Wuhan Jinyintan Hospital	Hubei Provincial Center for Disease Control and Prevention	Bin Fang et al
**EPI_ISL_412980**	Union Hospital of Tongji Medical College, Huazhong University of Science and Technology	Hubei Provincial Center for Disease Control and Prevention	Bin Fang et al
**EPI_ISL_412982**	Wuhan Lung Hospital	Hubei Provincial Center for Disease Control and Prevention	Bin Fang et al
**EPI_ISL_402125**	unknown	National Institute for Communicable Disease Control and Prevention (ICDC) Chinese Center for Disease Control and Prevention (China CDC)	Zhang et al
**EPI_ISL_402123**	Institute of Pathogen Biology, Chinese Academy of Medical Sciences & Peking Union Medical College	Institute of Pathogen Biology, Chinese Academy of Medical Sciences & Peking Union Medical College	Lili Ren et al
**EPI_ISL_403931**	Institute of Pathogen Biology, Chinese Academy of Medical Sciences & Peking Union Medical College	Institute of Pathogen Biology, Chinese Academy of Medical Sciences & Peking Union Medical College	Lili Ren et al
**EPI_ISL_403930**	Institute of Pathogen Biology, Chinese Academy of Medical Sciences & Peking Union Medical College	Institute of Pathogen Biology, Chinese Academy of Medical Sciences & Peking Union Medical College	Lili Ren et al
**EPI_ISL_403929**	Institute of Pathogen Biology, Chinese Academy of Medical Sciences & Peking Union Medical College	Institute of Pathogen Biology, Chinese Academy of Medical Sciences & Peking Union Medical College	Lili Ren et al
**EPI_ISL_403928**	Institute of Pathogen Biology, Chinese Academy of Medical Sciences & Peking Union Medical College	Institute of Pathogen Biology, Chinese Academy of Medical Sciences & Peking Union Medical College	Lili Ren et al
**EPI_ISL_402120**	National Institute for Viral Disease Control and Prevention, China CDC	National Institute for Viral Disease Control and Prevention, China CDC	Wenjie Tan et al
**EPI_ISL_402121**	National Institute for Viral Disease Control and Prevention, China CDC	National Institute for Viral Disease Control and Prevention, China CDC	Wenjie Tan et al
**EPI_ISL_434534**	National Institute for Viral Disease Control and Prevention, China CDC	National Institute for Viral Disease Control and Prevention, China CDC, Yunnan Provincial CDC	Wenjie Tan et al
**EPI_ISL_450500**	unknown	CAS Key Laboratory of Special Pathogens and Biosafety and Center for Emerging Infectious Diseases	Si et al

**EPI_ISL_406798**	General Hospital of Central Theater Command of People's Liberation Army of China	BGI & Institute of Microbiology, Chinese Academy of Sciences & Shandong First Medical University & Shandong Academy of Medical Sciences & General Hospital of Central Theater Command of People's Liberation Army of China	Weijun Chen et al
**EPI_ISL_406800**	General Hospital of Central Theater Command of People's Liberation Army of China	BGI & Institute of Microbiology, Chinese Academy of Sciences & Shandong First Medical University & Shandong Academy of Medical Sciences & General Hospital of Central Theater Command of People's Liberation Army of China	Weijun Chen et al
**EPI_ISL_406801**	General Hospital of Central Theater Command of People's Liberation Army of China	BGI & Institute of Microbiology, Chinese Academy of Sciences & Shandong First Medical University & Shandong Academy of Medical Sciences & General Hospital of Central Theater Command of People's Liberation Army of China	Weijun Chen et al
**EPI_ISL_408978**	Wuhan Fourth Hospital	Beijing Genomics Institute (BGI)	Weijun Chen et al
**EPI_ISL_406716**	unknown	State Key Laboratory of Virology, Wuhan University	Chen et al
**EPI_ISL_406717**	unknown	State Key Laboratory of Virology, Wuhan University	Chen et al
**EPI_ISL_449476**	unknown	Department of Respiratory and Critical Care	Wang et al
**EPI_ISL_449477**	unknown	Department of Respiratory and Critical Care	Wang et al
**EPI_ISL_449478**	unknown	Department of Respiratory and Critical Care	Wang et al
**EPI_ISL_449480**	unknown	Department of Respiratory and Critical Care	Wang et al
**EPI_ISL_449481**	unknown	Department of Respiratory and Critical Care	Wang et al
**EPI_ISL_449482**	unknown	Department of Respiratory and Critical Care	Wang et al
**EPI_ISL_449483**	unknown	Department of Respiratory and Critical Care	Wang et al
**EPI_ISL_449484**	unknown	Department of Respiratory and Critical Care	Wang et al
**EPI_ISL_449485**	unknown	Department of Respiratory and Critical Care	Wang et al
**EPI_ISL_449487**	unknown	Department of Respiratory and Critical Care	Wang et al
**EPI_ISL_402127**	Wuhan Jinyintan Hospital	Wuhan Institute of Virology, Chinese Academy of Sciences	Peng Zhou et al
**EPI_ISL_402124**	Wuhan Jinyintan Hospital	Wuhan Institute of Virology, Chinese Academy of Sciences	Peng Zhou et al
**EPI_ISL_402128**	Wuhan Jinyintan Hospital	Wuhan Institute of Virology, Chinese Academy of Sciences	Peng Zhou et al
**EPI_ISL_402129**	Wuhan Jinyintan Hospital	Wuhan Institute of Virology, Chinese Academy of Sciences	Peng Zhou et al
**EPI_ISL_402130**	Wuhan Jinyintan Hospital	Wuhan Institute of Virology, Chinese Academy of Sciences	Peng Zhou et al
**EPI_ISL_455460**	Jiangxi Province Center for Disease Control and Prevention	Jiangxi Province Center for Disease Control and Prevention	JianXiong Li et al
**EPI_ISL_455467**	Jiangxi Province Center for Disease Control and Prevention	Jiangxi Province Center for Disease Control and Prevention	JianXiong Li et al
**EPI_ISL_408480**	National Institute for Viral Disease Control and Prevention, China CDC	National Institute for Viral Disease Control & Prevention, CCDC	Wenjie Tan et al
**EPI_ISL_404227**	Zhejiang Provincial Center for Disease Control and Prevention	Department of Microbiology, Zhejiang Provincial Center for Disease Control and Prevention	Yin Chen et al
**EPI_ISL_404228**	Zhejiang Provincial Center for Disease Control and Prevention	Department of Microbiology, Zhejiang Provincial Center for Disease Control and Prevention	Yanjun Zhang et al

References

1. Baele, G., Lemey, P. & Suchard, M. A. Genealogical Working Distributions for Bayesian Model Testing with Phylogenetic Uncertainty. *Systematic Biology* vol. 65 250–264 (2016).
2. Suchard, M. A. *et al.* Bayesian phylogenetic and phylodynamic data integration using BEAST 1.10. *Virus Evol* **4**, vey016 (2018).
3. Kass, R. E. & Raftery, A. E. Bayes factors. *J. Am. Stat. Assoc.* **90**, 773–795 (1995).
4. Faria, N. R. *et al.* Zika virus in the Americas: Early epidemiological and genetic findings. *Science* **352**, 345–349 (2016).
5. Faria, N. R. *et al.* Establishment and cryptic transmission of Zika virus in Brazil and the Americas. *Nature* **546**, 406–410 (2017).
6. Metsky, H. C. *et al.* Zika virus evolution and spread in the Americas. *Nature* **546**, 411–415 (2017).
7. Lednicky, J. *et al.* Zika Virus Outbreak in Haiti in 2014: Molecular and Clinical Data. *PLoS Negl. Trop. Dis.* **10**, e0004687 (2016).
8. Dupont-Rouzeyrol, M. *et al.* Zika virus evolution on the edges of the Pacific ocean. *Emerg. Microbes Infect.* **6**, e111 (2017).

9. Delatorre, E., Fernández, J. & Bello, G. Investigating the Role of Easter Island in Migration of Zika Virus from South Pacific to Americas. *Emerging Infectious Diseases* vol. 24 2119–2121 (2018).

10. He, D., Gao, D., Lou, Y., Zhao, S. & Ruan, S. A comparison study of Zika virus outbreaks in French Polynesia, Colombia and the State of Bahia in Brazil. *Scientific Reports* vol. 7 (2017).

11. Lemey, P. *et al.* Unifying Viral Genetics and Human Transportation Data to Predict the Global Transmission Dynamics of Human Influenza H3N2. *PLoS Pathog.* **10**, e1003932 (2014).

12. Brockmann, D. & Helbing, D. The hidden geometry of complex, network-driven contagion phenomena. *Science* **342**, 1337–1342 (2013).
