# Supplementary material for: Accommodating individual travel history and unsampled diversity in Bayesian phylogeographic inference of SARS-CoV-2
Source: Nat Commun. 2020 Oct 9;11:5110. doi: 10.1038/s41467-020-18877-9 (PMC7547076; doi:10.1038/s41467-020-18877-9)
Supplement: Supplementary file 3 — Reporting Summary [file 41467_2020_18877_MOESM3_ESM.pdf]

nature research

Corresponding author(s): Philippe Lemey, Marc A. Suchard

Last updated by author(s): Sep 15, 2020

Reporting Summary

Nature Research wishes to improve the reproducibility of the work that we publish. This form provides structure for consistency and transparency in reporting. For further information on Nature Research policies, see our Editorial Policies and the Editorial Policy Checklist.

Statistics

For all statistical analyses, confirm that the following items are present in the figure legend, table legend, main text, or Methods section.

n/a	Confirmed	
[ ]	[x]	The exact sample size (*n*) for each experimental group/condition, given as a discrete number and unit of measurement
[ ]	[x]	A statement on whether measurements were taken from distinct samples or whether the same sample was measured repeatedly
[x]	[ ]	The statistical test(s) used AND whether they are one- or two-sided<br>*Only common tests should be described solely by name; describe more complex techniques in the Methods section.*
[ ]	[x]	A description of all covariates tested
[ ]	[x]	A description of any assumptions or corrections, such as tests of normality and adjustment for multiple comparisons
[ ]	[x]	A full description of the statistical parameters including central tendency (e.g. means) or other basic estimates (e.g. regression coefficient) AND variation (e.g. standard deviation) or associated estimates of uncertainty (e.g. confidence intervals)
[x]	[ ]	For null hypothesis testing, the test statistic (e.g. *F*, *t*, *r*) with confidence intervals, effect sizes, degrees of freedom and *P* value noted<br>*Give P values as exact values whenever suitable.*
[ ]	[x]	For Bayesian analysis, information on the choice of priors and Markov chain Monte Carlo settings
[x]	[ ]	For hierarchical and complex designs, identification of the appropriate level for tests and full reporting of outcomes
[x]	[ ]	Estimates of effect sizes (e.g. Cohen's *d*, Pearson's *r*), indicating how they were calculated

Our web collection on statistics for biologists contains articles on many of the points above.

Software and code

Policy information about availability of computer code

Data collection	No software as used for data collection. Genomic data was retrieved from GISAID.org and GENBANK
Data analysis	BEAST and TaxaMarkovJumpHistoryAnalyzer available at https://github.com/beast-dev/beast-mcmc (ancestral_path branch), MarkovJumpR available at https://github.com/beast-dev/MarkovJumpR, FigTree v1.4.4., Tracer v1.7.1, pangolin v1.1.14 (available at https://github.com/hCoV-2019/pangolin) using lineages data release 2020-05-19 (https://github.com/hCoV-2019/lineages), IQTREE v.2.0-rc1, TempEst v1.5.3, MAFFT v.7.453, Minimap2 v.2.17, the COG-UK pre-analysis pipeline (available at https://github.com/COG-UK/grapevine).

For manuscripts utilizing custom algorithms or software that are central to the research but not yet described in published literature, software must be made available to editors and reviewers. We strongly encourage code deposition in a community repository (e.g. GitHub). See the Nature Research guidelines for submitting code & software for further information.

Data

Policy information about availability of data

All manuscripts must include a data availability statement. This statement should provide the following information, where applicable:

- Accession codes, unique identifiers, or web links for publicly available datasets
- A list of figures that have associated raw data
- A description of any restrictions on data availability

BEAST XML input files including the GLM-diffusion model and travel history, with or without unsampled diversity, are available at https://github.com/phylogeography/travelHistory (DOI: 10.5281/zenodo.4027885). The SARS-CoV-2 genome data required for running these XMLs can be downloaded from www.GISAID.org. Supplementary Tables 2 to 5 list accession numbers for the genomes used in this study. Case count data was obtained from Our World in Data (https://ourworldindata.org/coronavirus-source-data). Air travel data was obtained from the International Air Transport Association (http://www.iata.org).

Field-specific reporting

Please select the one below that is the best fit for your research. If you are not sure, read the appropriate sections before making your selection.

☐ Life sciences ☐ Behavioural & social sciences ☒ Ecological, evolutionary & environmental sciences

For a reference copy of the document with all sections, see nature.com/documents/nr-reporting-summary-flat.pdf

Ecological, evolutionary & environmental sciences study design

All studies must disclose on these points even when the disclosure is negative.

Study description	phylogeographic reconstruction of the spread of SARS-CoV-2
Research sample	Genomic data from www.GISAID.org. Case count data for the relevant locations obtained from Our World in Data (https://ourworldindata.org/coronavirus-source-data). Air travel data, average daily symmetric fluxes, for the relevant locations obtained from the International Air Transport Association (http://www.iata.org)
Sampling strategy	No sampling strategy can be provided because GISAID collects data from numerous submitting labs with their own undocumented sampling strategy
Data collection	Genome data in www.GISAID.org is collected through submitting labs (Supplementary Tables 4 and 5) that use various sequencing technologies. Data in "Our world in Data" is collected by the "Our World in Data" team (https://ourworldindata.org/team). Air travel data in www.iata.org is collected by the International Air Transport Association.
Timing and spatial scale	All SARS-CoV-2 genomes available from December 2019 up to March 10, 2020. Air travel data consists of average daily symmetric fluxes from January and February, 2013. January and February are the two months that are most relevant for the time period of early global SARS-CoV-2 spread. Case count data from January 2020 to March 10, 2020. Spatial scale for all data: 44 global locations.
Data exclusions	Genomes with obvious sequencing errors, more than 1 genome from the same patients, genomes without adequate metadata, and outliers in a plot that regresses root-to-tip divergence against sampling time. Exclusion criteria were predetermined except for root-to-tip outliers (based on ad hoc residual determination).
Reproducibility	Analyses are reproducible through the xml files complemented with the genomic data from GISAID
Randomization	No randomization is applied or needed in phylogenetics
Blinding	No randomization is applied or needed in phylogenetics

Did the study involve field work? ☐ Yes ☒ No

Reporting for specific materials, systems and methods

We require information from authors about some types of materials, experimental systems and methods used in many studies. Here, indicate whether each material, system or method listed is relevant to your study. If you are not sure if a list item applies to your research, read the appropriate section before selecting a response.

Materials & experimental systems

n/a	Involved in the study
☒	☐ Antibodies
☒	☐ Eukaryotic cell lines
☒	☐ Palaeontology and archaeology
☒	☐ Animals and other organisms
☒	☐ Human research participants
☒	☐ Clinical data
☒	☐ Dual use research of concern

Methods

n/a	Involved in the study
☒	☐ ChIP-seq
☒	☐ Flow cytometry
☒	☐ MRI-based neuroimaging
